# Integrative metabolic analysis of orbital adipose/connective tissue in patients with thyroid-associated ophthalmopathy

**DOI:** 10.3389/fendo.2022.1001349

**Published:** 2022-11-18

**Authors:** Jiancheng Huang, Meng Chen, Yu Liang, Yuxiang Hu, Weiyi Xia, Yihan Zhang, Chen Zhao, Lianqun Wu

**Affiliations:** ^1^ Eye Institute, Eye and Ear, Nose & Throat (ENT) Hospital, Shanghai Medical College, Fudan University, Shanghai, China; ^2^ National Healthcare (NHC) Key Laboratory of Myopia, Fudan University, Shanghai, China; ^3^ Key Laboratory of Myopia, Chinese Academy of Medical Sciences, Shanghai, China; ^4^ Shanghai Key Laboratory of Visual Impairment and Restoration, Fudan University, Shanghai, China

**Keywords:** thyroid-associated ophthalmopathy, orbital adipose/connective tissue, LC-MS, metabolic profile, cholesterol metabolism

## Abstract

**Objective:**

Thyroid-associated ophthalmopathy (TAO) is a disfiguring autoimmune disease, which destroys the structure of orbital tissues and even threatens vision. Metabolic reprograming is critical in autoimmune diseases; however, the metabolic basis of TAO remains to be clarified. Our study aimed to reveal the metabolic profile of TAO.

**Methods:**

Orbital adipose/connective tissues from eleven TAO patients and twelve control subjects were collected during surgeries and analyzed with liquid chromatograph-mass spectrometer. Orthogonal partial least-squares discrimination analysis (OPLS-DA), variable importance in projection (VIP), heat map, and volcano plot were used to reveal metabolic profile in TAO. Pathway analysis and metabolites-gene analysis were utilized to explore potential metabolic metabolism in TAO.

**Results:**

3038 metabolites were detected in samples from the TAO patients and the controls. OPLS-DA analysis of the metabolomics results showed two distinguished groups, demonstrating that TAO has a unique metabolome. Univariate tests identified 593 dysregulated metabolites (*P* < 0.05), including 367 increased metabolites and 226 decreased metabolites. Pathway analysis showed that changed metabolites were enriched in cholesterol metabolism, choline metabolism in cancer, fat digestion and absorption, regulation of lipolysis in adipocytes, and insulin resistance. In addition, metabolites-gene analysis illustrated that cholesterol metabolism was involved in the pathogenesis of TAO. Endoplasmic reticulum stress-related genes (ATF6, PERK, and IRE1α) expressions were higher in TAO orbital tissues than in control orbital tissues verified by western blot. Additionally, the expression level of diacylglycerol acyltransferase 1 (DGAT1), a key metabolic protein for triacylglycerol synthesis, was increased in orbital tissues of TAO detected by qRT-PCR, indicating disrupted cholesterol metabolism in TAO.

**Conclusion:**

The present study demonstrated different metabolite profiles and potential metabolic mechanisms in TAO.

## Introduction

Thyroid-associated ophthalmopathy (TAO) is an autoimmune ocular condition frequently associated with thyroid dysfunction (1). Its annual incidence in the population is about 0.1% (2), and it is mainly characterized by proptosis, eyelid retraction, and lagophthalmos (redness of the eyelid, periorbital tissues, and conjunctiva). It is a potentially blinding disease that can severely diminish life quality (3). The fundamental pathological characteristic of TAO is the enlargement of the intraorbital contents, including the connective/adipose tissues and the extraocular muscle (4). However, the molecular mechanisms of TAO remain unclear.

Proptosis in TAO is primarily caused by an increase in the volume of extraocular muscles and orbital fat tissue. It has been reported that the enlargement of orbital fatty tissue contributes more toward the proptosis observed in TAO patients, than increased extraocular muscles volume (5). The inflamed and remodeled soft tissues of TAO around the eye lead to the dysfunction of adjacent structures (6). The primary autoantigen in TAO—thyroid-stimulating hormone receptor (TSHR)—was first validated through the identification of TSHR level in orbital specimens from TAO (7). Moreover, the adipogenesis of orbital adipose/connective tissue is correlated with increased TSHR (8). Increased fibrosis of orbital soft tissue is found in patients with TAO (9, 10). Taken together, biological changes of orbital tissue are critical in pathogenesis of TAO. However, the mechanism involved in the dysfunction of orbital fatty tissue in patients with TAO remains unclear.

Metabolic reprograming has been shown to be a hallmark of TAO. Metabolism has been recognized as a target for modulation in autoimmune diseases (11). Additionally, differentially expressed genes related to glucose metabolism might be important to the development of TAO (12). Cholesterol metabolism has been shown to have a positive association with thyroid-stimulating hormone (TSH) (13), and TSH leads to an increased amount of a rate-limiting enzyme involved in cholesterol biosynthesis: HMG-CoA reductase (14). Nicotinamide phosphoribosyltransferase, which is essential for ATP synthesis, is induced in TAO (15). However, how TAO alters its metabolism remains to be revealed. Thus, integrative analysis of metabolism is necessary to provide novel insights into its exact mechanisms in the development of TAO.

The metabolic condition of cell is reflected directly by the cell’s metabolites abundance. Metabolomics using a liquid chromatograph-mass spectrometer (LC-MS) is a quantitative and sensitive assessment for detecting metabolites (16–18). Herein, we aimed to reveal metabolic changes in orbital adipose/connective samples of patients with TAO utilizing LC-MS. Totally, 3038 metabolites were measured between the TAO patients and the control subjects. We found 593 dysregulated metabolites (*P* < 0.05), including 367 increased metabolites and 226 decreased metabolites. According to the pathway analysis and metabolites-gene analysis, cholesterol metabolism might be involved in the pathogenesis of TAO.

## Materials and methods

### Participants and sample collection

The study participants were TAO patients who had undergone orbital decompression surgery and normal subjects who had undergone blepharoplasty at the Eye, Ear, Nose and Throat (ENT) Hospital, Fudan University. TAO was diagnosed based on the Bartley criteria (19), and TAO activity was evaluated using Clinical Activity Score (CAS). A CAS ≥ 3 is indicative of active TAO, and a CAS ≤ 2 is indicative of inactive TAO (20). None of the TAO patients had any other inflammatory diseases, had undergone thyroidectomy or radioactive iodine therapy, or had received steroids or immunosuppressive agents in the previous six months. They had normal thyroid function after antithyroid treatment. We obtained orbital adipose/connective specimens removed during orbital decompression surgery for TAO patients. Normal orbital adipose/connective samples were collected during plastic surgeries. Fifteen TAO patients were selected for either metabolites analysis or western blot and quantitative real-time polymerase chain reaction (qRT-PCR) analysis. Clinical information of TAO patients and normal subjects enrolled for LC-MS was shown in **Table 1**. No significant distinction in gender or BMI was found between the two groups (**Table 1** **)**. Detailed characteristics of all TAO patients included in this study were summarized in **Table S3**. These samples were frozen in liquid nitrogen and stored at -80°C for analysis.

**Table 1:** Information of human subjects included in this study for metabolite analysis.

Characteristics	TAO(n = 11)	Control(n = 12)
Ages (years; Median, Range)	54.636 ± 14.493 (60, 30~70)	36.583 ± 13.048 (30, 23~66)
Gender		
Male	3	2
Female	8	10
Race/Ethnicity		
Chinese Han	11/11	12/12
BMI (Kg/m^2^; Median, Range)	23.157 ± 2.919 (22.432,18.043~27.942)	20.417 ± 2.157 (22.656, 17.715~23.529)
Serum cholesterol level (mmol/l; Median, Range)	4.695 ± 0.816 (4.78, 2.88~6.31)	4.591 ± 0.552 (4.635, 3.22~5.45)
CAS (Median, Range)	2.455 ± 1.499 (4, 0~5)	N/A

BMI, Body mass index; FBG, Fasting bloodglucose; CAS, Clinical activity score; N/A, Not applicable.

The study was approved by the Ethics Committee of Eye & ENT Hospital, Fudan University (identification number: 2021082), and conducted in compliance with the Helsinki Declaration.

### Sample preparation

Internal standard (2-chloro-l-phenylalanine in methanol, 0.3 mg/mL) (Shanghai Hengchuang Bio, Shanghai, China) and extraction solution with pre-chilled methanol in HPLC water (4/1, v/v) were added to each sample. Steel balls were added to the sample for homogenization. Then, sample was stored at -20°C for 30 min. The supernatant was transferred to a new tube after centrifugation and was then dried in a freeze concentration centrifugal dryer. Samples was reconstituted with methanol in HPLC-water (1/4, v/v) and then transferred to LC vials. Samples were stored at -80°C before LC-MS.

### Metabolite analysis of orbital adipose/connective tissue

LC-MS was performed as previously described in detail (21, 22). A DionexUltimate 3000 RS UHPLC system coupled with mass spectrometer equipped with electrospray ionization (ESI) source (Thermo Fisher Scientific, Waltham, MA, USA) was utilized to detect the metabolic profiling. The ACQUITY UPLC HSS T3 column (100 × 2.1 mm, 1.8 μm) was applied in both positive and negative modes. The gradient elution system was consisted of (A) (water with 0.1% formic acid, v/v) and (B) (acetonitrile containing 0.1% formic acid, v/v). The gradient elution was 5% B in 0.01min, 5% B in 2min, 30% B in 4min, 50% B in 8min, 80% B in 10min, 100% B in 14min, 100% B in 15min, 5% B in 15.1min and 5% B in 14min. Progenesis QI was applied to analyze LC-MS raw data (Waters Corporation, Milford, USA).

### qRT-PCR and western blot

qRT-PCR was done as previously described (23). Total RNAs were isolated and purified from orbital adipose/connective sample using the TRIzol reagent (Thermo Fisher Scientific, Waltham, MA, USA). Complementary DNA was synthesized utilizing the PrimeScript RT Reagent Kit (Takara, Otsu, Shiga, Japan) following the manufacturer’s instructions. Gene expression level was quantified by qRT-PCR using SYBR Premix Taq (Takara, Otsu, Shiga, Japan) and normalized to β-actin. All primer sequence information is listed in **Table 2**.

**Table 2:** Primer information.

Name	Forward (5’-3’)	Reverse (5’-3’)
human β-actin	gcagaaggagatcactgccct	gctgatccacatctgctggaa
human DGAT1	cccaccatccagaactccat	ttcaggcaggagtggaagag

The tissue was lysed by RIPA buffer containing proteinase inhibitors and phosphatase inhibitors (Roche, Basel, Switzerland). Protein concentration was evaluated by BCA protein assay kit (Vayme Biotechnology, Nanjing, China). The proteins were separated on 4-16% gradient SDS-PAGE, and then transferred to PVDF membrane (Millipore, Bedford, MA, USA). The detection of chemiluminescence was performed as previously described (24).Western blot results were quantified using ImageJ (http://rsb.info.nih.gov/ij/index.html). Primary antibodies involved in IRE1α (CST, cat.No: 3294, 1:1000), PERK (CST, cat.No: 5683, 1:1000), ATF6 (CST, cat.No: 65880, 1:1000), and β-Actin (CST, cat.No: 4967, 1:1000) were used for western blot.

### Statistics

Metabolomics data were analyzed using orthogonal partial least-squares discrimination analysis (OPLS-DA), variable importance in projection (VIP), heat map, and volcano plot (25). Pathway enrichment for the changed metabolites was analyzed using MBRole 2.0 (http://csbg.cnb.csic.es/mbrole2/) based on the Kyoto Encyclopedia of Genes and Genomes (KEGG). Interactions between metabolites and genes were analyzed using the MetScape plugin (v3.1.3) (26) in Cytoscape (3.7.1) (27)and networks generated based on known gene-metabolite interactions. Statistical analyses were conducted by prism (GraphPad, San Diego, CA, USA). The difference was determined by two-tailed Student’s *t* test. Data were shown as mean ± SEM. *P* < 0.05 was considered statistically significant.

## Results

### TAO alters metabolic profiles in orbital adipose/connective tissue

**Figure 1:**
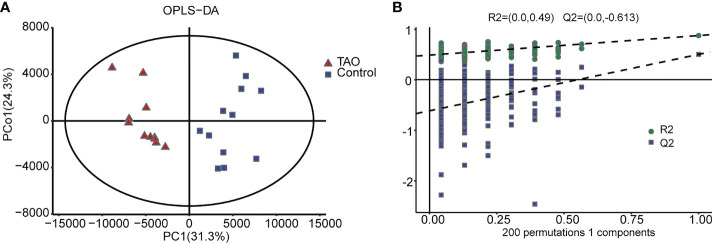
Orthogonal partial least-squares discrimination analysis (OPLS-DA). **(A)** The OPLS-DA score plot clearly discriminates thyroid-associated ophthalmopathy (TAO) group from the control group. **(B)** Validation plots of 200 permutation tests of the OPLS-DA model for TAO group and normal group.

We measured 3038 metabolites in samples from the TAO patients and the controls. OPLS-DA revealed a prominent distinction between the two groups in score plots, indicating that the metabolomes of orbital tissue from the patients with TAO were significantly changed**(** **Figure 1A** **)**. Then, cross validation test was performed to validate the OPLS-DA model. The Q2 of -0.613 is less than 0.05, indicating high predictive relevance of the OPLS-DA model**(** **Figure 1B** **)**.

**Figure 2:**
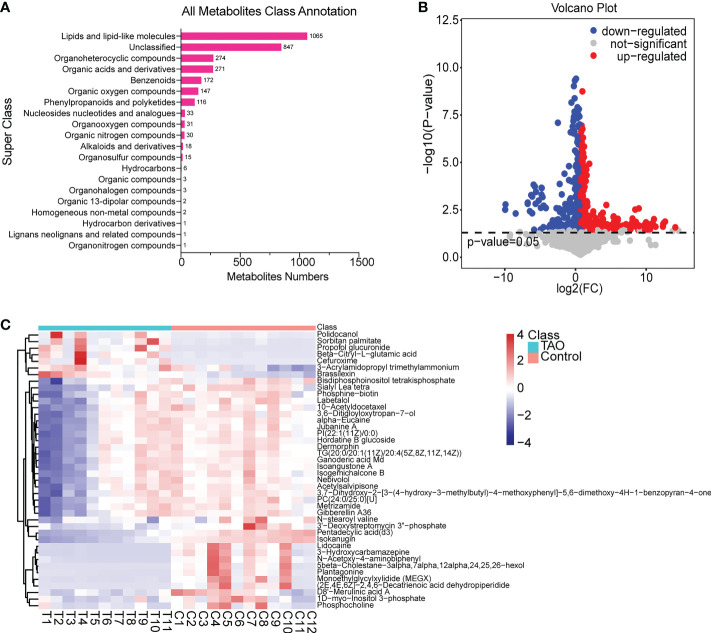
Overview of changed metabolites. **(A)** Class annotation of the changed metabolites. **(B)** Volcano plot of changed metabolites. **(C)** Heat map of 42 changed metabolites (VIP scores > 1, *P* < 0.05).

All metabolite class annotation was analyzed, about 1/3 changed metabolites occurred in lipids and lipid-like molecules (1065) **(** **Figure 2A** **)**. To differentiate the altered metabolites in TAO, we performed volcano plot analysis with a threshold of *P* < 0.05. Among the 3038 metabolites, 593 metabolites (367 increased metabolites and 226 decreased metabolites) were significantly changed in the TAO samples **(** **Figure 2B** **)**. The changed metabolites were evaluated by calculating VIP score. High VIP score indicates significant group discrimination and 1 was selected as a cutoff for VIP score. Forty-two metabolites (VIP score > 1) were found. Notably, lidocaine, 5β-cholestane, monoethylglycylxylidide, triglyceride, pentadecylic acid, and 3’-deoxystreptomycin were the top altered metabolites (VIP score > 1.8) (**Table S1**). To visualize the changed pattern of metabolites, we chose the 42 changed metabolites (VIP scores > 1, P < 0.05) for heat map analysis **(** **Figure 2C**, **Table S1** **)**.

**Figure 3:**
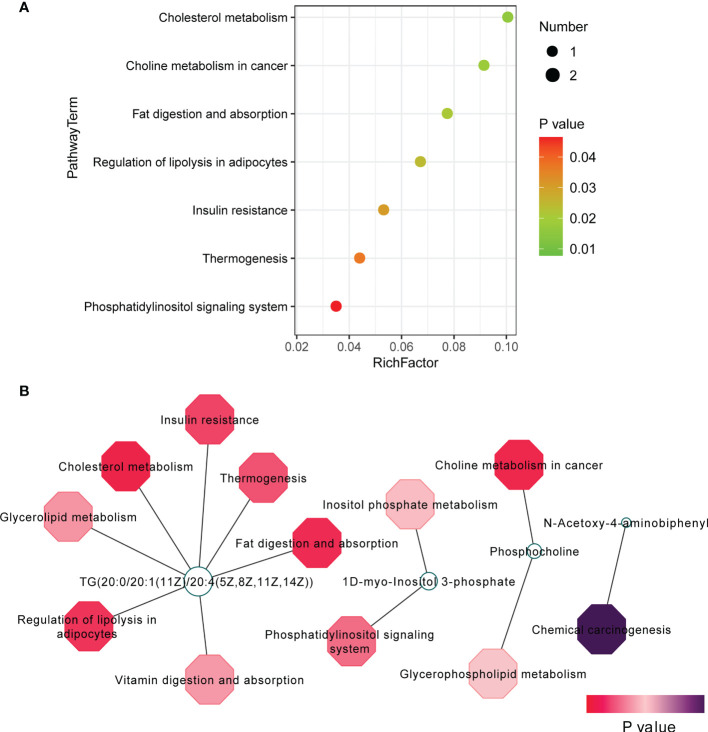
Pathway analysis of 42 changed metabolites (VIP scores > 1, *P* < 0.05). **(A)** Pathway analysis was visualized in bubble diagrams of TAO group and control group. **(B)** Interaction of metabolites and pathways was shown.

To explore the potential mechanism of the pathogenesis of TAO, we identified the enriched pathways by analyzing 42 changed metabolites (VIP scores > 1, *P* < 0.05). The significantly altered metabolic pathways were cholesterol metabolism, choline metabolism in cancer, fat digestion and absorption, regulation of lipolysis in adipocytes, and insulin resistance (**Figure 3A**). Metabolites-pathway associated analysis was shown in **Figure 3B**. TG(20:0/20:1(11Z)/20:4(5Z,8Z,11Z,14Z)), 1D-myo-Inositol 3-phosphate, and phosphocholine were involved in the changed metabolic pathway **(** **Figure 3B** **)**. These metabolomic findings demonstrated that orbital adipose/connective tissue in TAO has a unique metabolic characteristic.

### Biological significance of metabolites

**Figure 4:**
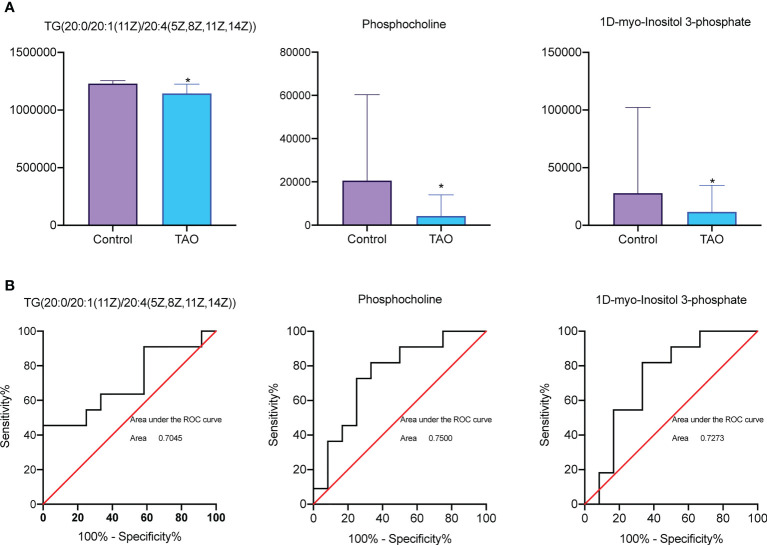
Receiver operating characteristic (ROC) curve analysis and bar chart of differential metabolites. **(A)** Comparison of TG (20:0/20:1(11Z)/20:4(5Z,8Z,11Z,14Z)), 1D-myo-Inositol 3-phosphate, and phosphocholine level between TAO group and control group (* *P* < 0.05). **(B)** ROC curve analysis of TG(20:0/20:1(11Z)/20:4(5Z,8Z,11Z,14Z)), 1D-myo-Inositol 3-phosphate, and phosphocholine in c TAO group and control group.

TG(20:0/20:1(11Z)/20:4(5Z,8Z,11Z,14Z)), 1D-myo-Inositol 3-phosphate, and phosphocholine (VIP scores > 1, *P* < 0.05) were involved in predicted pathway (**Figure 4A**). To further elucidate the importance of these metabolites, receiver operating characteristic (ROC) curve was utilized to analyze these metabolites for sensitivity and specificity as biomarkers. High area under the curve (AUC) indicates good sensitivity and specificity. These metabolites had high AUC > 0.7 (TG(20:0/20:1(11Z)/20:4(5Z,8Z,11Z,14Z)): 0.7045; 1D-myo-Inositol 3-phosphate: 0.7273; phosphocholine: 0.7500) (**Figure 4B**).

### Cholesterol metabolism is the potential metabolic mechanism in TAO

**Figure 5:**
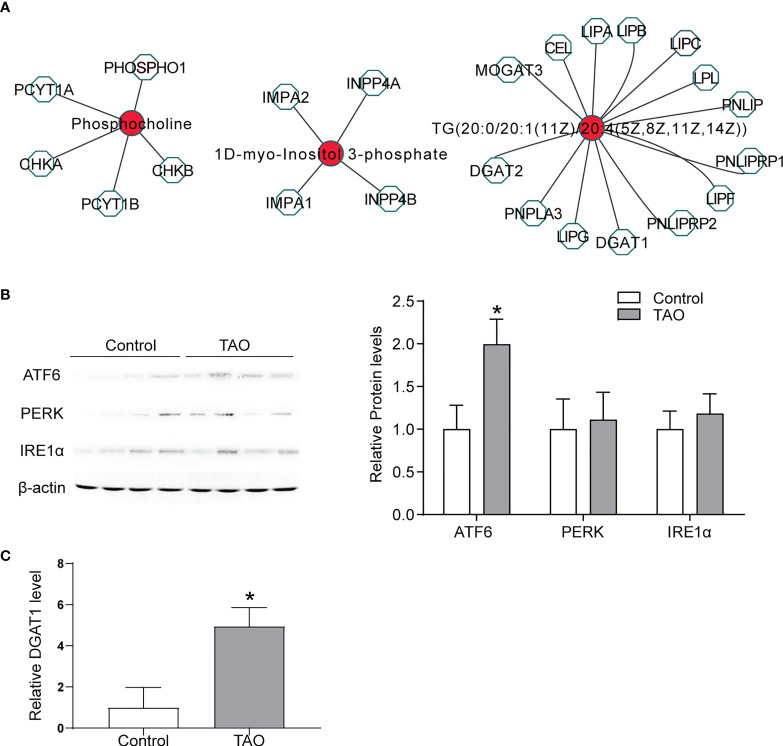
Biological significance of metabolites. **(A)** Networks of the metabolites and genes (fold change > 2) were visualized utilizing Metscape 3. **(B)** ATF6, PERK, and IRE1α expressions were detected by western blot in orbital adipose/connective tissue from TAO patients (n = 4) and control donors (n = 4) (* *P* < 0.05). **(C)** DGAT1 expression was detected by qRT-PCR in orbital adipose/connective tissue from TAO patients (n = 3) and control donors (n = 3) (* *P* < 0.05).

Significantly changed expression profiles of mRNAs in TAO were found in our previous study (23). To determine the mechanism of the changed metabolites, we analyzed the differentially abundant metabolites and genes(> 2 fold change)based on known gene-metabolite interactions. Metscape network analysis were subsequently performed to integrate the transcriptomics and metabolomics data of TAO (**Figure 5A**). Cholesterol-related gene diacylglycerol acyltransferase 1 (DGAT1) was associated with TG (20:0/20:1(11Z)/20:4(5Z,8Z,11Z,14Z)) (**Figure 5A**). DGAT1 is a critical rate-limiting enzyme in the biosynthesis of triacylglycerol (TG) (28). TG synthesis by DGAT1 protects adipocyte from endoplasmic reticulum (ER) stress and adipose tissue inflammation, which is correlated with the etiology of TAO (29, 30). ER stress induces the response of unfolded protein, involving transmembrane receptors activation (ATF6, PERK and IRE1α) (31). Herein, an increased expression level of ATF6, PERK and IRE1α was found in orbital tissues of TAO examined by western blot indicating ER stress in TAO (**Figure 5B**). Additionally, statistically increased expression level of ATF6 was found in TAO sample (**Figure 5B**). qRT-PCR showed that DGAT1 was highly expressed in orbital tissues of TAO patients (**Figure 5C**). Taken together, these results indicated that dysfunction of cholesterol metabolism might be involved in TAO pathogenesis.

## Discussion

TAO is a complex autoimmune disease, which causes orbital disfigurement, diplopia and even irreversible vision loss. Orbital soft tissue undergoes inflammation and remodeling in TAO (32). Nevertheless, the mechanism of TAO has not been completely clarified. Recently, cell metabolism has become highly involved in the mechanisms of autoimmune diseases pathogenesis. In TAO, TSAb induced the process of autoimmune stimulating the increased production of thyroid hormones (33). Thyroid hormones play an important role in regulating cholesterol metabolism, glucose metabolism, and lipid metabolism (34, 35). Consequently, the dysfunction of cell metabolism is supposed to be associated with the pathogenesis of TAO. It is urged to explore metabolic profile in TAO. In this study, the metabolic signatures of orbital adipose/connective specimens obtained from the TAO patients and normal participants were explored. Totally, 593 significantly changed metabolites (367 upregulated and 226 downregulated) were found in the orbital adipose/connective samples of TAO patients. Pathway analysis illustrated that 42 significantly changed metabolites (VIP scores > 1, *P* < 0.05) are strongly related to cholesterol metabolism, choline metabolism in cancer, fat digestion and absorption, regulation of lipolysis in adipocytes, and insulin resistance. The important differentially expressed mRNAs involved in the pathogenesis of TAO have been clarified in our previous study (23). Herein, a powerful tool, Metscape was utilized to conduct integrative metabolomics and transcriptomics analysis to explore molecular pathways for metabolites and identify the key metabolites involved in TAO. In conclusion, metabolites-pathways analysis and Metscape network analysis indicated that disruption of cholesterol metabolism is a potential metabolic mechanism in TAO. The results of this study might provide an avenue for understanding the pathogenesis of TAO in the perspective of metabolism.

### TAO influences cholesterol metabolism

Herein, a significant change of triglyceride was found (*P* < 0.05; VIP > 2) in orbital fat tissue samples from the patients with TAO. Pathway analysis and metabolite-genes analysis showed cholesterol metabolism was potentially involved in TAO pathogenesis. Similarly, in our previous KEGG analysis of different circular RNAs of TAO orbital adipose/connective tissue indicates cholesterol metabolism pathway is changed (36). Disrupted cholesterol metabolism altered immunity in TAO. Cholesterol metabolism dysfunction promotes inflammatory responses, including the activation of Toll like receptor signaling (37), inflammasome (38), release of pro-inflammatory cytokines (39), and phagocytes production (40). In the other hand, impaired proinflammatory response in adipose cause abnormal lipid metabolism, which causes adipose remodeling and expansion (41). These changes lead to serious complications of TAO such as proptosis and vision loss (optic nerve compression) (42). The mechanism contributing to proinflammatory response of orbital adipose tissues in TAO patients is not entirely clarified. In our study, ER stress and increased DGAT1 expression were found in the orbital adipose/connective tissue of TAO. Adipocyte is protected from adipose inflammation and ER stress by DGAT1-mediated TG synthesis (29, 30). We speculate that disrupted cholesterol metabolism in TAO leads to ER stress. To protect from ER stress and adipose tissue inflammation, DGAT1 was upregulated to promote TG synthesis in TAO. Taken together, it is indicated that that TAO potentially reprograms cholesterol metabolism for its pro-inflammatory process.

### TAO and insulin resistance

According to the pathway analysis, insulin resistance is involved in TAO. In our previous study, insulin resistance is the top15 enriched hypomethylated differential methylated probes (DMPs) KEGG pathway (43). Insulin resistance is associated with the insulin like growth factor 1 receptor (IGF-1R) (44). IGF-1R is a member of insulin receptor (IR) family regulating development, growth, and cell transformation (45). IGF-1R expression level is elevated in TAO patients (46). The immunoglobulins in TAO patients that stimulate orbital fibroblasts might act through IGF-1R (47, 48). IGF-1R combines with TSH receptor (TSHR), the main autoantigen of TAO, to form a functional complex (49). Blocking the activity of IGF-1R suppresses the inflammatory reaction caused by IgG in TAO (50). Furthermore, IGF-1/IGF-1R regulates adipocyte differentiation (adipogenesis) (51, 52). Normal orbital adipose tissue maintains local physiological homeostasis. This homeostasis is disrupted by increased adipogenesis in TAO patients (8). Induced adipogenesis in adipocyte cause insulin resistance (53). Taken together, enhanced adipogenesis activated by IGF-1R leading to insulin resistance could be the potential molecular mechanism in TAO.

Considering that many factors affect metabolite changes in TAO, studies with larger sample sizes and various clinical stages are necessary to clarify the metabolite profiles in TAO. In addition, tracing these changed pathways with stable isotopes and exploring the role of the important enzymes are necessary to reveal the interactions of metabolic pathways.

In summary, A total of 593 metabolites differed significantly between TAO and control participates. Our study indicates a significant difference in the metabolic profiles of TAO, showing that TAO alters cellular metabolome, especially cholesterol metabolism. This study should shed light on the molecular pathogenesis of TAO and provide therapeutic benefits of targeting the changed metabolic pathways

## Data availability statement

The original contributions presented in the study are included in the article/**Supplementary Material**. Further inquiries can be directed to the corresponding authors.

## Ethics statement

The studies involving human participants were reviewed and approved by Ethics Committee of Eye & ENT Hospital, Fudan University. The patients/participants provided their written informed consent to participate in this study.

## Author contributions

JH, LW, and CZ designed research. JH, LW, MC, YL, WX, YH, YZ, and CZ performed research. JH, LW, MC, YL, WX, YH, YZ, and CZ analyzed data. JH, LW, and CZ wrote the paper. All authors contributed to the article and approved the submitted version.

## Funding

This study was supported by the Shanghai Natural Science Foundation (20ZR1409800 to LW), the National Natural Science Foundation of China (82271126 and 81600765 to LW.; 8201001029 and 81730025 to CZ), Shanghai Outstanding Academic Leaders (2017BR013 to CZ), and Excellent Academic Leaders of Shanghai (18XD1401000 to CZ).

## Acknowledgments

We thank Professor Jiang Qian (Eye Institute, Eye and ENT Hospital, Shanghai Medical College, Fudan University) for providing orbital adipose/connective tissues.

## Conflict of interest

The authors declare that the research was conducted in the absence of any commercial or financial relationships that could be construed as a potential conflict of interest.

## Publisher’s note

All claims expressed in this article are solely those of the authors and do not necessarily represent those of their affiliated organizations, or those of the publisher, the editors and the reviewers. Any product that may be evaluated in this article, or claim that may be made by its manufacturer, is not guaranteed or endorsed by the publisher.
