# Corrigendum: Integrative metabolic analysis of orbital adipose/connective tissue in patients with thyroid-associated ophthalmopathy

**DOI:** 10.3389/fendo.2023.1296678

**Published:** 2023-12-14

**Authors:** Jiancheng Huang, Meng Chen, Yu Liang, Yuxiang Hu, Weiyi Xia, Yihan Zhang, Chen Zhao, Lianqun Wu

**Affiliations:** ^1^ Eye Institute, Eye and Ear, Nose & Throat (ENT) Hospital, Shanghai Medical College, Fudan University, Shanghai, China; ^2^ National Healthcare (NHC) Key Laboratory of Myopia, Fudan University, Shanghai, China; ^3^ Key Laboratory of Myopia, Chinese Academy of Medical Sciences, Shanghai, China; ^4^ Shanghai Key Laboratory of Visual Impairment and Restoration, Fudan University, Shanghai, China

**Keywords:** thyroid-associated ophthalmopathy, orbital adipose/connective tissue, LC-MS, metabolic profile, cholesterol metabolism

In the published article, there was an error in the Funding statement. One of the numbers for the funding received by Lianqun Wu from the National Natural Science Foundation of China was incorrectly displayed as “***8227040531***”, when it should have been “82271126”. The correct Funding statement appears below.

**FUNDING**

This study was supported by the Shanghai Natural Science Foundation (20ZR1409800 to LW), the National Natural Science Foundation of China (82271126 and 81600765 to LW.; 8201001029 and 81730025 to CZ), Shanghai Outstanding Academic Leaders (2017BR013 to CZ), and Excellent Academic Leaders of Shanghai (18XD1401000 to CZ).

The authors apologize for this error and state that this does not change the scientific conclusions of the article in any way. The original article has been updated.

