# Supplementary material for: Integrative metabolic analysis of orbital adipose/connective tissue in patients with thyroid-associated ophthalmopathy
Source: Front Endocrinol (Lausanne). 2022 Nov 18;13:1001349. doi: 10.3389/fendo.2022.1001349 (PMC9718489; doi:10.3389/fendo.2022.1001349)
Supplement: Supplementary file 1 [file DataSheet_1.pdf]

Supporting Information

Integrative metabolic analysis of orbital adipose/connective tissue in patients with thyroid-associated ophthalmopathy

Jiancheng Huang [1,2,3,4#], Meng Chen [1,2,3,4#], Yu Liang[1,2,3,4], Yuxiang Hu[1,2,3,4], Weiyi Xia[1,2,3,4], Yihan Zhang [1,2,3,4], Chen Zhao[1,2,3,4*], Lianqun Wu[1,2,3,4*]

[1]Eye Institute, Eye and ENT Hospital, Shanghai Medical College, Fudan University, Shanghai, China,

[2]NHC Key Laboratory of Myopia (Fudan University), Shanghai, China,

[3]Key Laboratory of Myopia, Chinese Academy of Medical Sciences, Shanghai, China,

[4]Shanghai Key Laboratory of Visual Impairment and Restoration (Fudan University), Shanghai, China

#These authors contributed equally to this work.

[*]Corresponding authors: Chen Zhao, MD, PhD, 83 Fenyang Road, Eye Institute, Eye and ENT Hospital, Shanghai Medical College, Fudan University Shanghai 200031, China; E-mail: dr_zhaochen@163.com; Lianqun Wu, MD, 83 Fenyang Road, Eye Institute, Eye and ENT Hospital, Shanghai Medical College, Fudan University Shanghai 200031, China; E-mail: lianqun.wu@fdeent.org.

Table S1: The 42 Changed metabolites (VIP scores >1, $P < 0.05$)

Metabolites	VIP	P-value
Lidocaine	71.62792	0.0002962
5beta-Cholestane-3alpha,7alpha,12alpha,24,25,26-hexol	8.3694819	0.0042164
Monoethylglycylxylidide (MEGX)	3.1870714	0.0026089
TG(20:0/20:1(11Z)/20:4(5Z,8Z,11Z,14Z))	2.0351934	0.0320583
Pentadecylic acid(d3)	1.9862886	1.708E-08
3'-Deoxystreptomycin 3''-phosphate	1.8308487	0.0061883
1D-myo-Inositol 3-phosphate	1.7483533	0.0299901
N-stearoyl valine	1.7377459	0.0214619
PI(22:1(11Z)/0:0)	1.6995924	0.0248961
3-Hydroxycarbamazepine	1.6955651	0.0002036
alpha-Eucaine	1.64557	0.0258134
Dermorphin	1.581996	0.0265596
PC(24:0/25:0)[U]	1.5702667	0.0119852
Hordatine B glucoside	1.4783728	0.0206325
Polidocanol	1.4505317	0.0360032
Plantagonine	1.4003793	0.0034554
Phosphocholine	1.3983132	0.0371031
Ganoderic acid Md	1.3735078	0.0238119
(2E,4E,6Z)-2,4,6-Decatrienoic acid dehydropiperidide	1.3699492	0.0051033
Nebivolol	1.3641309	0.0392149
Isokanugin	1.3521873	5.296E-10
Beta-Citryl-L-glutamic acid	1.3439076	0.0095874
Acetylsalvipisone	1.3362991	0.0277448
Bisdiphosphoinositol tetrakisphosphate	1.3340017	0.0151964
Propofol glucuronide	1.3299771	0.0036384
Isoangustone A	1.313381	0.0291971
N-Acetoxy-4-aminobiphenyl	1.3129126	0.0009121
Cefuroxime	1.2567258	0.0179145
Jubanine A	1.2195809	0.038945
3-Acrylamidopropyl trimethylammonium	1.1967105	0.0192912
Isogemichalcone B	1.1828619	0.0345664
Metrizamide	1.1719736	0.0457379
3,6-Ditigloyloxytropan-7-ol	1.170505	0.0256981
3,7-Dihydroxy-2-[3-(4-hydroxy-3-methylbutyl)-4-methoxyphe	1.1456985	0.0250489
Sorbitan palmitate	1.0913451	0.008069
Sialyl Lea tetra	1.0737411	0.0017822
10-Acetyldocetaxel	1.0710594	0.0106691
D8'-Merulinic acid A	1.0458743	0.0079761
Phosphine-biotin	1.0174519	0.0341307
Gibberellin A36	1.0133272	0.0255695
Brassilexin	1.012522	0.0004533
Labetalol	1.0019624	0.0136805

Table S2: List of metabolites and their parameters detected by LC-MS

Metabolites	Compound ID	m/z	Retention time (min)	Ion mode
(-)-11-hydroxy-9,10-dihydrojasmonic acid	45942	229.143958	7.188033333	pos
(-)-12-hydroxy-9,10-dihydrojasmonic acid	45945	273.135136	5.643033333	neg
(-)-7-epi--9,10-dihydrojasmonic acid	36074	257.139935	7.622216667	neg
(-)-alpha-Bisabolol	LMPR0103060001	223.206076	12.50038333	pos
(-)-Blebbistatin	45431	337.119505	9.16185	neg
(-)-Euphomine	LMPR0104300003	579.29373	0.6755	pos
(-)-Fusicoplagin A	53690	437.254019	10.63578333	neg
(-)-Jasmonic acid	3345	211.133295	0.566216667	pos
(-)-Menthol	LMPR0102090001	157.159019	0.505683333	pos
(-)-Nopol	86655	149.132735	0.463416667	pos
(-)-Salvisyriacolide	53744	481.281672	9.81545	neg
(-)-trans-C75	96552	255.158231	9.5911	pos
({8-[2-(2,4-dihydroxyphenyl)-7-hydroxy-4-oxo-4H-chı	HMDB0129622	503.135924	8.641916667	pos
({8-[2-(2,4-dihydroxyphenyl)-7-hydroxy-4-oxo-4H-chı	HMDB0129630	503.136042	8.41665	pos
(+)-12-hydroxy-9Z-hexadecenoic acid	LMFA01050270	315.218296	9.794333333	neg
(+)-5-trans Cloprostenol	43458	847.323719	14.3394	neg
(+)-7-epi--9,10-dihydrojasmonic acid	36073	423.276017	10.61491667	neg
(+)-Camphor	41065	135.117176	0.484533333	pos
(+)-Comphene	41088	137.132823	1.907233333	pos
(+)-Gallocatechin	47219	611.138187	2.593133333	neg
(+)-Neomenthol	HMDB0035763	195.114899	7.147033333	pos
(+)-Pinoresinol 4-O-[beta-D-Glucopyranosyl-(1->2)-[	HMDB0040669	889.300915	14.54056667	neg
(+)-trans-C75	96547	255.159409	7.16755	pos
(+)-trans-Carveol glucoside	86544	315.180479	7.618183333	pos
(+)-Vulgraon B	53475	217.195575	0.4423	pos
(+/-)-[R-(E)]-5-Isopropyl-8-methylnona-6,8-dien-2-on	88390	177.164179	11.53246667	pos
(+/-)11,12-DiHETrE	LMFA03050008	361.235494	11.47181667	pos
(+/-)14,15-DiHETrE	LMFA03050010	361.233846	10.73525	pos
(+/-)8,9-DiHETrE	LMFA03050006	361.235674	11.09911667	pos
(+/-)-Ethyl 2-hydroxy-2-methylbutyrate	LMFA07010693	129.091404	0.02505	pos
(±)-(Z)-2-(5-Tetradecenyl)cyclobutanone	HMDB0037543	282.27963	13.05981667	pos
(±)-1,4-Nonanediol diacetate	HMDB0037184	245.175249	8.96225	pos
(±)-2-Methyl-3-(2-methylphenyl)propanal	HMDB0040245	207.102504	7.455083333	neg
(±)-Propionylcarnitine	85176	218.139163	2.214583333	pos
(±)-Rollipyrrole	92411	311.137188	9.836716667	pos
(1(10)E,4a,5E)-1(10),5-Germacradiene-12-acetoxy-4	HMDB0038798	341.197454	9.77325	neg
(10S)-Juvenile hormone III acid diol	64602	271.190838	9.7543	pos
(10S)-Juvenile hormone III diol	64605	285.206219	10.20713333	pos
(10S,11S)-Pterosin C	HMDB0030763	252.159757	2.503183333	pos
(12S,15S)-15-O-Demethyl-10,29-dideoxy-11,12-dihy	88922	435.273029	10.59398333	pos
(1alpha,3beta,20S,22R,24S,25S)-Pubescenin	86662	601.339567	12.2697	neg
(1R,2R)-3-oxo-2-pentyl-cyclopentanebutanoic acid	LMFA02010017	285.171443	7.938566667	neg
(1R,2R)-3-oxo-2-pentyl-cyclopentanehexanoic acid	74959	313.202938	9.857533333	neg
(1S,2S)-3-oxo-2-pentyl-cyclopentanebutanoic acid	74961	285.171408	7.49725	neg
(1S,2S)-3-oxo-2-pentyl-cyclopentanehexanoic acid	74960	313.202922	9.541616667	neg
(1S,2S,4S,5S)-2,4,7-Thujanetriol 4-glucoside	HMDB0033645	349.184024	15.43553333	pos
(1S,Z)-3-((S)-3-hydroxy-2-methylpropylidene)-1-met	263740	257.222725	7.003433333	pos
(21-Methyl-8Z-pentatriacontene	LMFA11000079	522.598462	15.29111667	pos
(22E)-3beta-Hydroxy-5alpha-chola-16,22-dien-24-oi	LMST04010340	390.298625	12.01588333	pos
(23R)-3alpha,7alpha,12alpha,23-Tetrahydroxy-5beta	42837	447.270655	4.65325	pos

(24E)-3alpha,15alpha-Diacetoxy-23-oxo-7,9(11),24-	HMDB0035388	567.333724	12.43053333	neg
(24S)-1alpha,24-dihydroxy-22-oxa-20-epivitamin D3	LMST03020059	436.343184	0.92605	pos
(25R)-3alpha,7alpha-dihydroxy-5beta-cholestan-27-	57994	542.350896	10.16615	pos
(25S)-3-oxo-12beta-acetoxy-cholest-1,4-dien-26-oic	84902	502.352035	13.02021667	pos
(25S)-5alpha-cholestan-3beta,4beta,6alpha,7alpha,8	83923	501.342994	9.7543	pos
(25S)-5alpha-cholestan-3beta,6alpha,7beta,8beta,1	LMST01010327	502.375047	12.0361	pos
(2E,4E,6Z)-2,4,6-Decatrienoic acid dehydropiperidid	HMDB0033530	249.196566	5.363416667	pos
(2E,4Z,7Z)-2,4,7-Tridecatrienal	89355	210.185704	8.150466667	pos
(2-Naphthyl)methanol	HMDB0060303	176.107368	8.921266667	pos
(2-phenyl-7H-chromen-7-ylidene)oxidanium	HMDB0133410	445.146668	9.857533333	neg
(2R*,3R*)-1,2,3-Butanetriol	HMDB0034778	129.052697	15.92241667	pos
(2R,6R,7S,8S)-7-ethyl-2-propyl-1-azaspiro[5.5]unde	263668	284.223653	11.43826667	neg
(2S)-2-amino-3-[4-hydroxy-3-(sulfooxy)phenyl]-2-me	HMDB0142153	272.024168	5.116983333	neg
(2S)-OMPT	44913	447.235412	10.92508333	neg
(2S,3R)-2-[(1R)-1-[3,5-bis(trifluoromethyl)phenyl]eth	HMDB0061012	418.105378	4.295183333	neg
(2S,3S)-2-hydroxytridecane-1,2,3-tricarboxylic acid	45917	331.177096	7.096583333	neg
(2S,4R)-4-(9H-Pyrido[3,4-b]indol-1-yl)-1,2,4-butanet	HMDB0035191	543.224678	10.44721667	neg
(2S,4R,5S)-Muscarine	86601	213.112694	5.548633333	pos
(2S,4S)-Monatin	95722	331.07053	0.712516667	pos
(2S,4S)-Pinnatanine	86268	243.09898	1.112466667	neg
(2S,5R)-5-(hex-5-en-1-yl)-2-propyldecahydroquinolir	263793	281.2956	10.6551	pos
(2S,5R)-5-methyl-2-propyldecahydroquinoline	263763	234.161973	0.357716667	pos
(2S,5R,8aS)-5-methyl-2-propyldecahydroquinoline	263764	234.16252	10.34803333	pos
(2S,5S)-5-methyl-2-propyldecahydroquinolin-6-ol	263768	256.192314	8.339283333	neg
(2-trans,6-trans)-farnesal	LMPR0103010012	221.190157	0.02505	pos
(2xi,6xi)-7-Methyl-3-methylene-1,2,6,7-octanetetrol	89084	187.133143	1.844116667	pos
(2Z)-2-[(sulfooxy)methyl]but-2-enoic acid	HMDB0129117	218.994214	0.7678	pos
(3-aminopropoxy)sulfonic acid	HMDB0140100	138.022689	4.219483333	pos
(3b,16a,21b,22a)-12-Oleanene-3,16,21,23,28-pento	88753	631.384061	0.6532	neg
(3b,4b,5b)-4,5-Epoxy-p-menth-1-en-3-ol 3-glucoside	93587	329.161373	7.769866667	neg
(3beta,6beta)-Furanoeremophilane-3,6-diol 6-acetat	HMDB0034930	310.201849	1.5132	pos
(3beta,8beta)-3-Hydroxy-7(11)-eremophilen-12,8-oli	HMDB0040754	251.164603	0.548016667	pos
(3beta,9beta)-7-Drimene-3,11,12-triol	HMDB0036053	299.1871	8.465833333	neg
(3-Methylphenyl)methyl acetate	HMDB0041473	209.082036	9.393866667	neg
(3R)-3-isopropenyl-6-oxoheptanoic acid	45862	229.108311	5.326966667	neg
(3R,2'S)-Myxol 2'-(2,4-di-O-methyl-alpha-L-fucoside)	64117	776.548298	15.3633	pos
(3R,5Z)-5-Octene-1,3-diol	HMDB0030368	183.080844	0.39995	pos
(3R,6S)-3-Hydroxy-1,7-dioxaspiro[5.5]undecane	LMPK09000005	343.213361	10.08896667	neg
(3R,7R)-1,3,7-Octanetriol	HMDB0033625	163.13319	5.898866667	pos
(3S)-3-isopropenyl-6-oxoheptanoic acid	45863	229.108297	6.00115	neg
(3S,4S)-3-hydroxytetradecane-1,3,4-tricarboxylic aci	45918	369.189095	9.774883333	pos
(3S,5R,6R,7E)-3,5,6-Trihydroxy-7-megastigmen-9-o	HMDB0038736	225.148952	7.331416667	pos
(3S,5S)-Carbapenam-3-carboxylic acid	71641	309.109949	3.263033333	neg
(3S,6E)-Nerolidol	HMDB0041629	195.174814	0.02505	pos
(3S,6S)-3-Hydroxy-1,7-dioxaspiro[5.5]undecane	LMPK09000006	217.10815	5.158833333	neg
(3Z,6Z)-3,6-Nonadienal	HMDB0031152	156.138607	0.37885	pos
(3Z,6Z,9Z)-dodecatrienol	LMFA05000097	225.149749	7.559183333	neg
(4E,6E,d14:2) sphingosine	53907	242.212016	9.570466667	pos
(4E,8E,10E-d18:3)sphingosine	53913	318.241083	11.13923333	pos
(4E,8E,d18:2) sphingosine	LMSP01080010	280.264141	10.695	pos
(4OH,8Z,t18:1) sphingosine	53909	338.267059	9.878016667	pos
(4'R,6R,6'S,7S,8R)-6'-(2-hydroxypentan-2-yl)-4',8-di	263722	364.244178	5.837283333	pos
(4R,6S)-4-Hydroxy-1,7-dioxaspiro[5.5]undecane	LMPK09000007	217.10816	5.36915	neg
(5alpha,8beta,9beta)-5,9-Epoxy-3,6-megastigmadie	HMDB0034672	209.154182	10.6551	pos
(5-butyl-6-methyloctahydroindolizin-8-yl)methanol	263914	270.208174	9.18295	neg

(5-D-ribonyl)hopane	LMPR04000021	581.39764	10.36856667	pos
(5E)-(24R)-24,25-dihydroxy-[6,19,19-trideutrio]vitam	42065	442.338296	7.986766667	pos
(5E,10E)-19-(3-carboxylpropyl)vitamin D3	42423	509.340277	8.762433333	pos
(5-hydroxy-7-methoxy-2,2-dimethyl-3,4-dihydro-2H-1	HMDB0125817	327.052076	0.641016667	pos
(5R)-5-methyl-6-((Z)-1,2,3,8a-tetrahydroindolizin-6(5	263736	258.18188	3.951716667	pos
(5R,6S)-5,6-Epoxy-7-megastigmen-9-one	HMDB0034984	209.154154	10.24565	pos
(6E,8E)-4,6,8-Megastigmatriene	HMDB0035180	221.154646	9.62565	neg
(6R)-3,6-dimethyl-7-((8R,Z)-8-methylhexahydroindol	263732	278.248526	10.4506	pos
(6R)-vitamin D2 6,19-sulfur dioxide adduct	41911	461.309925	15.98305	pos
(6R,8Z)-6-Hydroxy-3-oxotetradecenoic acid	HMDB0062363	301.166366	7.81205	neg
(6S)-dehydrovomifoliol	53371	223.133297	8.702383333	pos
(6S,8Z)-6-Hydroxy-3-oxotetradecenoic acid	HMDB0062364	301.166331	5.600916667	neg
(7R)-8-((8S,Z)-8-hydroxy-8-methylhexahydroindolizi	263694	343.296203	8.921266667	pos
(7R,8R,E)-6-((2R)-6-hydroxy-2,5-dimethyloctylidene)	263715	649.515113	14.3394	neg
(7Z)-14-hydroxy-10,13-dioxoheptadec-7-enoic acid	LMFA01050450	313.200157	8.4781	pos
(8S,Z)-6-((2R)-5-hydroxy-2-methylhexylidene)-8-met	263689	290.20966	10.26573333	pos
(8S,Z)-6-((2R,E)-6-hydroxy-2,5-dimethylhept-4-en-1-	263700	316.225506	10.85683333	pos
(9R,13R)-1a,1b-dihomo-jasmonic acid	LMFA02010010	221.154035	0.002433333	pos
(9R,13R)-1a,1b-dinor-10,11-dihydro-12-oxo-15-phyt	36060	311.187178	9.499383333	neg
(9S,10S)-10-hydroxy-9-(phosphonooxy)octadecanoi	LMFA00000008	377.21058	9.878583333	neg
(9xi,10xi,12xi)-9,10-Dihydroxy-12-octadecenoic acid	HMDB0031679	297.243077	11.13923333	pos
(9Z,12Z,15Z)-3-hydroxyoctadecatrienoylcarnitine	LMFA07070027	455.346454	13.07931667	pos
(all-E)-3,5,7-Tridecatriene-9,11-diyn-1-ol	93201	231.102958	7.159866667	neg
(E)-10-Oxo-8-decenoic acid	HMDB0040883	229.108307	5.537733333	neg
(E)-1-Propenyl 2-propenyl disulfide	HMDB0041393	184.985991	0.548016667	pos
(E)-2-O-Cinnamoyl-beta-D-glucopyranose	HMDB0035880	328.137839	8.96225	pos
(E)-3-Nonen-1-ol	HMDB0041601	143.14338	0.484533333	pos
(E,E)-2,4-Decadienoic isobutylamide	87312	241.22776	5.6311	pos
(E,E)-Di-1-propenyl disulfide	HMDB0041390	184.985964	11.67358333	pos
(R) 2,3-Dihydroxy-3-methylvalerate	LMFA01050452	131.070687	1.048966667	pos
(R)-(+)-2-Pyrrolidone-5-carboxylic acid	63632	147.076789	0.712516667	pos
(R)-1,3-Octanediol	LMFA05000564	129.127789	0.4423	pos
(R)-1-O-[b-D-Apiofuranosyl-(1->2)-b-D-glucopyranos	HMDB0032798	419.266117	1.7812	pos
(R)-1-O-[b-D-Glucopyranosyl-(1->6)-b-D-glucopyran	HMDB0032799	488.271304	5.918766667	pos
(R)-2,3-Dihydroxy-isovalerate	LMFA01050453	173.021351	0.7301	pos
(R)-2-Hydroxysterculic acid	87380	355.249913	10.94575	neg
(R)-2-Methylimino-1-phenylpropan-1-ol	63538	208.097843	10.46825	neg
(R)-3-((R)-3-Hydroxybutanoyloxy)butanoate	63260	189.076499	5.263766667	neg
(R)-3-Hydroxy-5-phenylpentanoic acid	HMDB0031517	239.092845	6.717266667	neg
(R)-3-hydroxybutyrylcarnitine	HMDB0062735	248.14979	1.090316667	pos
(R)-Bitalin A	HMDB0040358	236.128636	2.441183333	pos
(R)-mevalonate	HMDB0059629	131.070674	1.9283	pos
(R)-Shinanolone	HMDB0030580	237.077059	8.212733333	neg
(R)-S-Lactoylglutathione	3511	360.086389	4.1905	neg
(S)-2,3,4,5-Tetrahydropyridine-2-carboxylate	63463	145.097475	2.052666667	pos
(S)-2-Acetolactate	58568	177.039914	2.196783333	neg
(S)-3-Methyl-2-oxopentanoic acid	5478	259.119275	5.032616667	neg
(S)-3-Octanol glucoside	HMDB0032958	315.178537	7.023933333	pos
(S)-5-Diphosphomevalonic acid	HMDB0001090	309.014648	0.69355	pos
(S)-9-Hydroxy-10-undecenoic acid	LMFA01050436	245.139803	6.14865	neg
(S)-alpha-Terpinyl glucoside	HMDB0029856	317.19644	8.859766667	pos
(S)C(S)S-S-Methylcysteine sulfoxide	HMDB0029432	134.027416	0.8856	pos
(S)-N-(4,5-Dihydro-1-methyl-4-oxo-1H-imidazol-2-yl)	HMDB0034912	168.077222	15.98305	pos
(S)-Pterosin P	HMDB0036607	252.159846	0.2524	pos
(S)-Reticuline	6967	312.15983	10.67518333	pos

(S)-Spinacine	86559	333.130302	9.246216667	neg
(S)-Spirobrassinin	HMDB0035974	231.006352	7.054383333	neg
(S)-Verimol F	HMDB0036558	317.140086	10.7592	neg
(S,E)-Lyratol propanoate	HMDB0031869	209.154014	0.002433333	pos
(Z)-15-Oxo-11-eicosenoic acid	HMDB0029797	347.256078	4.65325	pos
(Z)-2-Nonen-1-ol	LMFA05000570	187.133591	9.246216667	neg
(Z)-3-(1-Formyl-1-propenyl)pentanedioic acid	88983	239.031108	9.125	pos
(Z)-3-Hexenal	HMDB0031498	195.138759	9.857533333	neg
(Z)-3-Oxo-2-(2-pentenyl)-1-cyclopenteneacetic acid	HMDB0030197	253.108594	7.2653	neg
(Z)-4-Dodecenal	LMFA06000256	179.179747	0.02505	pos
(Z)-4-Hydroxy-6-dodecenoic acid lactone	HMDB0032331	219.135773	13.9131	pos
(Z)-7-(5-((1E,3E,6Z,9Z)-dodeca-1,3,6,9-tetraen-1-yl)	LMFA04060001	410.254509	1.698516667	pos
(Z)-8-Decene-4,6-diyn-1-yl 3-methylbutanoate	HMDB0031000	277.145063	10.88373333	neg
(Z)-9-Cycloheptadecen-1-one	HMDB0031336	233.227012	0.463416667	pos
(Z,Z)-3,6-Dodecadien-1-ol	LMFA05000573	200.201323	10.7758	pos
[4-(3-oxopentyl)phenyl]oxidanesulfonic acid	HMDB0133002	257.049213	10.1101	neg
[5-(2-{[(2E)-3-(3,4-dihydroxyphenyl)prop-2-enoyl]oxy	HMDB0124949	435.040225	9.646666667	neg
[6-(2H-chromen-2-ylidene)cyclohexa-2,4-dien-1-ylid	HMDB0133408	445.144346	8.52915	neg
[6-(5,7-dihydroxy-4-oxo-2-phenyl-4H-chromen-8-yl)-	HMDB0127267	465.048465	4.211566667	neg
[Nitrilotris(methylene)]trisphosphonic acid	HMDB0029807	281.968537	1.090316667	pos
{[(2Z)-2-{2-[2-(2,4-dihydroxyphenyl)-7-hydroxy-4-oxo	HMDB0129618	503.135995	8.048083333	pos
{[(6E)-3-oxo-1,7-diphenylhepta-4,6-dien-1-yl]oxy}sul	HMDB0133921	403.084646	5.7905	neg
{2-hydroxy-5-[(2E)-3-phenylprop-2-enoyl]phenyl}oxic	HMDB0135412	358.997494	0.69355	pos
{2-hydroxy-5-[2-hydroxy-3-(2,4,6-trihydroxyphenyl)pr	HMDB0127736	395.039691	0.641016667	pos
{3-[(1E)-5-oxo-7-phenylhepta-1,3-dien-1-yl]phenyl}o:	HMDB0133923	403.084652	6.085383333	neg
{4-[(E)-2-(2,3,5-trihydroxyphenyl)ethenyl]phenyl}oxic	HMDB0128516	342.06258	5.528016667	pos
{5-[(1E)-3-{[3-(3,4-dihydroxyphenyl)-1-methoxy-1-ox	HMDB0124948	435.040143	4.948316667	neg
{5-[5,7-dihydroxy-3-(3,4,5-trihydroxybenzoyloxy)-3,4	HMDB0128193	545.036405	10.38908333	pos
1-(2,3-Dihydro-1H-pyrrolizin-5-yl)-2-propen-1-one	HMDB0040028	200.047657	5.507416667	pos
1-(2,6,6-Trimethyl-2-cyclohexen-1-yl)-1,6-heptadien-	HMDB0029704	271.146478	9.1661	pos
1-(2H-1,3-Benzodioxol-5-yl)-2-[2,6-dimethoxy-4-(pro	93839	475.176052	15.90565	neg
1-(2-Hydroxyphenylamino)-1-deoxy-beta-D-gentiobi	92408	504.137198	5.348033333	neg
1-(2-methoxy-tetradecanyl)-sn-glycero-3-phosphoetl	LMGP02060009	424.28306	13.17741667	pos
1-(2-Pyrimidyl)piperazine	1450	165.113835	15.43553333	pos
1-(2-Thienyl)-1-butanone	HMDB0032935	199.043271	5.57985	neg
1(3)-glyceryl-PGD2	LMFA03010184	409.25783	10.08378333	pos
1(3)-glyceryl-PGF2alpha	LMFA03010181	446.313274	10.04253333	pos
1-(3-Furanyl)-6,7-dihydroxy-4,8-dimethyl-1-nonanon	92894	313.166483	8.360383333	neg
1-(4-Fluorobenzyl) piperazine	96603	212.156342	0.37885	pos
1-(4-Methoxyphenyl)-1-penten-3-one	HMDB0030905	191.1071	9.473266667	pos
1-(5-Acetyl-2-hydroxyphenyl)-3-methyl-1-butanone	88547	221.117743	10.71513333	pos
1-(5-Hydroxy-2-pyrimidinyl)piperazine	1453	163.098198	15.98305	pos
1-(9H-Pyrido[3,4-b]indol-1-yl)-1,4-butanediol	90513	257.129058	9.391516667	pos
1-(gamma-Glutamylamino)cyclopropanecarboxylic a	HMDB0031701	275.089232	1.09225	neg
1-(Methylsulfinyl)propyl 1-propenyl disulfide	88950	255.019234	5.495583333	neg
1-(Methylsulfinyl)propyl propyl disulfide	88953	257.034975	5.263766667	neg
1-(sn-Glycero-3-phospho)-1D-myo-inositol	62398	333.059918	0.710516667	neg
1,1'-(Tetrahydro-6a-hydroxy-2,3a,5-trimethylfuro[2,3	HMDB0032527	257.103636	5.179716667	neg
1,1,2-Triphenylpropane	69935	273.163556	6.7366	pos
1,11-Undecanedicarboxylic acid	6622	243.160505	8.972016667	neg
1,17-Heptadecanediol	95424	255.268476	0.357716667	pos
1,2,10-Trihydroxydihydro-trans-linalyl oxide 7-O-beta	HMDB0033237	400.218511	5.672333333	pos
1,2,3-Tris(1-ethoxyethoxy)propane	92130	309.22772	6.24675	pos
1,2,4,5,7-Pentathiocane	HMDB0039431	246.905741	8.022933333	neg
1,2-Benzisothiazol-3(2H)-one	89917	152.016837	6.30855	pos

1,2-Bis(1-ethoxyethoxy)propane	HMDB0037163	238.201662	6.165383333	pos
1,2-Bis(4-hydroxyphenyl)-2-propanol	69606	262.144348	9.632383333	pos
1,2-Diamino-4-nitrobenzene	73087	136.0509	15.94263333	pos
1,2-Dihydro-1,1,6-trimethylnaphthalene	94809	190.159397	5.115883333	pos
1',2'-Dihydro-1,1'-dimethyl-2'-oxo-4,4'-bipyridinium(1	90497	240.065974	0.566216667	pos
1,2-Dihydronaphthalene-1,2-diol	HMDB0060335	163.075684	7.741033333	pos
1,2-Dihydroxy-3,4-epoxy-1,2,3,4-tetrahydronaphthal	70318	196.097264	5.055316667	pos
1,2-Dihydroxy-3-keto-5-methylthiopentene	HMDB0012134	185.023803	0.584866667	pos
1,2-Dihydroxynaphthalene-6-sulfonate	71140	285.008077	4.211566667	neg
1,3,5-TRIMETHOXYBENZENE	43830	149.060029	5.853733333	neg
1,3,7-Trimethyluric acid	HMDB0002123	255.073094	7.0755	neg
1,4-Benzenediol, 2,6-bis(1-methylethyl)-, 4-(hydroge	2160	273.081003	6.211833333	neg
1,4-Dihydroxynaphthalene	70319	161.060028	0.37885	pos
1,4-dimethylcyclohexane	98013	130.159371	15.3633	pos
1,4-Ipomeadiol	87001	215.092465	5.7905	neg
1,5-Naphthalenediamine	73157	176.118614	0.08705	pos
1,6-Dihydroxy-5-methylcyclohexa-2,4-dienecarboxyl	66502	171.065555	1.970383333	pos
1,8-Cineol	LMPR0102090019	155.143342	2.255616667	pos
10,11-dihydro-20-trihydroxy-leukotriene B4	HMDB0012503	385.224021	8.803283333	neg
10,11-epoxy-3,7,11-trimethyl-2E,6E-tridecadienoic a	34693	267.196013	9.71345	pos
10,11-Epoxy-7Z-hexadecenoic acid	LMFA01070026	313.202932	9.267316667	neg
10,20-Dihydroxyeicosanoic acid	HMDB0031923	362.327035	8.96225	pos
10-Acetyldocetaxel	73551	830.335427	13.85283333	neg
10-Deoxymethymycin	LMPK04000035	471.341615	11.25935	pos
10-Deoxymethynolide	40998	319.188536	10.34803333	pos
10-dodecen-1-ol	LMFA05000025	202.216968	7.270016667	pos
10E,12Z-Hexadecadienal	LMFA06000200	254.248329	12.01588333	pos
10E,12Z-Octadecadienoic acid	HMDB0005048	298.274727	9.672883333	pos
10-F2-dihomo-IsoP	96931	414.322176	11.17941667	pos
10-fluoro-capric acid	96778	208.169954	13.50863333	pos
10-hydroxy-2E-decenoic acid	LMFA01050157	231.124013	5.57985	neg
10-Hydroxycarbazepine	66662	253.098132	8.5924	neg
10-Hydroxygeraniol	64042	339.25476	11.58091667	neg
10-Isopropyl-2,7-dimethyl-1-oxaspiro[4.5]deca-3,6-d	91202	189.16413	0.02505	pos
10-keto tridecanoic acid	LMFA01060042	273.171504	7.580016667	neg
10-oxo-docosanoic acid	74777	399.31229	11.19298333	neg
10-oxo-nonadecanoic acid	LMFA01060128	357.265582	10.92508333	neg
10-propyl-5,9-tridecadien-1-ol	36472	256.264049	12.33913333	pos
10-tridecynoic acid	LMFA01030603	228.196234	8.662066667	pos
10-Undecen-2-one	LMFA12000157	213.149588	7.875283333	neg
10-undecenal	LMFA06000067	186.185655	0.548016667	pos
10Z-Heneicosene	LMFA11000067	312.363108	11.57286667	pos
10Z-heptadecenoic acid	LMFA01030283	286.274485	10.10433333	pos
10Z-Nonadecenoic acid	HMDB0013622	314.305859	10.6551	pos
11,12,14-Trihydroxy-7-methoxy-8,11,13-abietatrien-:	HMDB0031932	399.178751	11.65356667	pos
11,12,15S-trihydroxy-5Z,8Z,13E-eicosatrienoic acid	36275	372.275029	10.32751667	pos
11,12-DiHETrE	HMDB0002314	339.253568	10.75555	pos
11,14,15-trihydroxy-5Z,8Z,12E-eicosatrienoic acid	36276	355.248619	10.14556667	pos
11-amino-undecanoic acid	LMFA01100004	184.169999	0.39995	pos
11-dehydro-2,3-dinor-TXB2	74988	341.196148	7.392883333	pos
11-Dehydrocorticosterone	57880	343.192035	12.18908333	neg
11-deoxy-PGF1alpha	36139	358.295835	10.16615	pos
11-dodecen-1-ol	LMFA05000021	202.216957	6.287966667	pos
11E,13Z-Hexadecadienal	46491	219.211186	0.002433333	pos
11-hydroperoxy-12,13-epoxy-9-octadecenoic acid	LMFA02000106	346.259523	9.632383333	pos

11-Hydroxy-9-tridecenoic acid	LMFA01050437	273.171491	7.854216667	neg
11-nitro-1-undecene	HMDB0062669	200.164962	6.798166667	pos
11S-HpODE	LMFA02000115	313.237925	10.9175	pos
11Z-Eicosenal	LMFA06000248	312.326611	15.1473	pos
12(13)-EpOME	63352	297.242765	0.484533333	pos
12,13-DHOME	HMDB0004705	297.243023	11.75366667	pos
12,13-DiHOME	LMFA02000230	337.236249	10.4506	pos
12,13-dihydroxy-11-methoxy-9-octadecenoic acid	LMFA01080006	345.264324	10.59398333	pos
12a-Hydroxydolineone	48044	333.041211	4.169433333	neg
12alpha-Hydroxy-3-oxo-5beta-cholan-24-oic Acid	LMST04010168	408.309299	15.98305	pos
12-amino-dodecanoic acid	35924	216.196295	6.962383333	pos
12-hydroxy-5,8,10-heptadecatrienoic acid	74573	325.202615	10.55208333	neg
12-Hydroxydodecanoic acid	HMDB0002059	234.206899	5.938783333	pos
12-hydroxyjasmonic acid	45941	209.117628	6.103533333	pos
12-keto-10,11,14,15-tetrahydro-LTB4	LMFA03020004	356.280145	10.695	pos
12-methyl-10-oxo-tridecanoic acid	LMFA01060096	287.187135	8.52915	neg
12-Methyltridecanal	LMFA06000257	213.221682	0.002433333	pos
12-oxo-10Z-dodecenoic acid	74732	257.139981	8.065116667	neg
12-Oxo-2,3-dinor-10,15-phytodienoic acid	88174	309.171475	9.857533333	neg
12-oxo-9(Z)-dodecenoic acid	35852	257.139995	6.422383333	neg
12-oxo-9Z-dodecenoic acid	LMFA01060167	257.139964	6.211833333	neg
12-oxo-ETE	36285	363.216214	10.1101	neg
12S-HHTrE	LMFA03050002	325.202713	9.920666667	neg
12S-hydroxy-16-heptadecynoic acid	74526	300.254047	10.28636667	pos
13,14-dihydro-15-keto PGF2alpha isopropyl ester	45685	397.295846	11.17941667	pos
13,14-dihydro-15-keto-PGF1alpha	36215	355.249914	11.3567	neg
13,14-dihydro-15-keto-tetranor PGF1alpha	2294	318.228086	6.941883333	pos
13,14-dihydro-15-keto-tetranor PGF1beta	45724	318.228062	8.27325	pos
13,14-dihydro-16,16-difluoro Prostaglandin D2	45569	391.228274	4.014216667	pos
13,14-dihydro-PGE1	LMFA03010144	401.255316	9.2884	neg
13-beta-D-Glucosyloxydocosanoate	66113	501.377367	12.94041667	pos
13-chloro-5,14-dihydroxy-7,12-dimethyl-6-(3-methyll	HMDB0139187	401.078724	6.085383333	neg
13-hexadecenoic acid	73938	272.259089	11.79395	pos
13-Hydroxy-9-methoxy-10-oxo-11-octadecenoic acic	95353	387.239765	8.86655	neg
13-hydroxy-9-methoxy-10-oxooctadec-11-enoic acid	LMFA02000288	343.248515	9.7543	pos
13-Hydroxyabscisic acid	91261	261.114036	9.098566667	neg
13-Hydroxylupanine	64452	303.14783	15.92241667	pos
13-Hydroxypergolide glucuronide	1794	487.191416	10.23661667	neg
13-Oxo-9,11-tridecadienoic acid	HMDB0034564	269.139718	8.318183333	neg
13-OxoODE	HMDB0004668	339.218213	10.29983333	neg
13R-HODE	LMFA02000035	341.233984	10.46825	neg
13S-HpOTrE	LMFA02000114	309.207934	10.61491667	neg
13Z-hexadecenoic acid	73939	255.232401	1.028233333	pos
14,14,14-Trifluoro-11E-tetradecenyl acetate	46326	307.188281	8.655633333	neg
14,14,14-Trifluoro-11Z-tetradecenyl acetate	46327	307.189814	10.57303333	neg
14,15-DiHETrE	HMDB0002265	339.25363	10.4711	pos
14:0(5Me[R],9Me[R],13Me)	LMPR0103010014	288.290225	9.227683333	pos
14-F2-dihomo-IsoP	96932	383.277466	13.2908	pos
14-fluoro-myristic acid	96779	264.23255	7.392883333	pos
14-hydroxy-5Z-tetradecenoic acid	LMFA01050181	287.187137	7.138766667	neg
14-keto pentadecanoic acid	LMFA01060049	301.202719	8.613483333	neg
14R,15S-EpETrE	LMFA03080001	319.228596	11.37706667	neg
14R-hydroxy-11E-eicosenoic acid	LMFA01050256	371.281106	10.57303333	neg
14S-Methyl-1-octadecene	LMFA11000019	284.331663	11.01845	pos
15(S)-HETE-biotin	45652	578.375172	5.569266667	pos

15(S)-HpEDE	35361	323.258767	10.93775	pos
15-Deacetylcalonectrin	HMDB0035724	353.159741	7.096583333	neg
15-Hydroxynorandrostene-3,17-dione glucuronide	HMDB0010353	445.188714	11.80461667	neg
15-oxo-hexadecanoic acid	LMFA01060105	271.227427	11.23941667	pos
16,16-dimethyl PGF2beta	36153	400.306488	11.47181667	pos
16,16-dimethyl-PGD2	36123	425.255603	10.08896667	neg
16,16-dimethyl-PGE1	36128	383.279812	10.30701667	pos
16,16-dimethyl-PGE2	36136	403.245706	10.93775	pos
16,17S-DHA-epoxide	75212	387.216341	11.37706667	neg
16:0 Campesteryl ester	103360	637.590053	14.1585	neg
16beta-Fluoroandrost-4-ene-3,17-dione	70389	349.1817	11.0077	neg
16-Hydroxy hexadecanoic acid	HMDB0006294	290.269523	8.880233333	pos
16-hydroxy-6-hexadecenoic acid	LMFA01050272	315.218299	8.571316667	neg
16-phenyl-tetranor-PGE2	LMFA03010066	353.17693	11.0077	neg
17,20-dimethyl Prostaglandin F1alpha	45618	402.322287	10.36856667	pos
17,21-Epoxy-9-fluoro-11beta-hydroxypregn-4-ene-3,	70782	407.18844	15.96818333	neg
17-F2-dihomo-IsoP	96930	400.306527	10.61448333	pos
17-hydroxyandrostane-3-glucuronide	LMST05010059	449.254967	10.73845	neg
17-Hydroxylinolenic acid	HMDB0011108	339.218261	9.667733333	neg
17-Hydroxypregnenolone sulfate	LMST05020021	393.172354	10.67736667	neg
17-Hydroxyprogesterone	HMDB0000374	311.202388	11.80461667	neg
17-O-Acetylajmaline	71120	386.243676	11.99565	pos
18-carboxy dinor Leukotriene B4	45639	361.162856	8.41665	pos
18-Hydroxycorticosterone	LMST02030091	363.215075	10.10433333	pos
18-Hydroxycortisol	57881	396.2389	1.090316667	pos
18-Oxocortisol	5321	394.223253	0.846116667	pos
18-oxo-nonadecanoic acid	LMFA01060129	357.2656	10.2577	neg
18Z-Heptacosen-10-one	98217	415.390279	13.82548333	pos
19-(3-methyl-butanoyloxy)-villanovane-13alpha,17-d	53639	467.302203	10.6567	neg
1-Acetoxy-2-hydroxy-16-heptadecen-4-one	LMFA05000594	327.253743	10.59398333	pos
1alpha,25-Dihydroxy-16-ene-19-nor-24-oxovitamin [	42535	434.325312	14.43878333	pos
1alpha,25-dihydroxy-21-nor-20-oxavitamin D3	41970	422.327337	1.167366667	pos
1alpha,25-dihydroxy-24-oxo-22-oxavitamin D3	LMST03020039	471.249843	9.473266667	pos
1alpha,25-dihydroxy-2beta-(4-hydroxybutoxy)vitamir	42437	505.390827	15.07588333	pos
1-Benzylimidazole	63058	181.072431	4.160016667	pos
1beta,25-dihydroxy-22-oxavitamin D3	LMST03020062	436.343176	0.584866667	pos
1-Dehydro-9-fluoro-11-oxotestololactone	70857	377.140306	10.55208333	neg
1-dimethylarsinoyl-(3Z,6Z,9Z,12Z,15Z,18Z)-docosal	LMFA11000717	436.255167	9.391516667	pos
1D-myo-Inositol 3-phosphate	HMDB0006814	259.022918	0.710516667	neg
1-Docosene	LMFA11000312	326.378781	10.97813333	pos
1-Dodecene	HMDB0059874	186.22203	6.185533333	pos
1-Guanidino-1-deoxy-scyllo-inositol 4-phosphate	64554	601.13024	0.884666667	neg
1-Hydro-1,1a-dihydroxy-9-fluorenone	71163	215.070134	0.712516667	pos
1-Hydroxy-3-methyl-9H-carbazole	89307	242.082654	10.2577	neg
1'-Hydroxy-4-keto-gamma-carotene glucoside/ 1'-OH	41426	747.522119	15.3633	pos
1-Hydroxyepiacorone	87286	297.171353	9.056366667	neg
1-Isothiocyanato-2-(methylthio)ethane	HMDB0038442	151.035576	3.102033333	pos
1-Isothiocyanato-3-phenylpropane	93135	353.114365	8.276	neg
1-Methoxy-1-(2,4,5-trimethoxyphenyl)-2-propanol	87929	257.138851	5.672333333	pos
1-Methoxy-1H-indole-3-acetonitrile	HMDB0040973	225.043372	0.4423	pos
1-Methoxy-3-(4-hydroxyphenyl)-2E-propenal 4'-gluc	HMDB0034755	307.117115	10.0258	neg
1-Methoxyspirobrassinin	89833	325.03273	2.155183333	neg
1-Methyl-2-oxopropyl butyrate	91376	203.092307	4.737533333	neg
1-Methyl-3-(2-thiazolyl)-1H-indole	90335	213.049227	0.6902	neg
1-methyl-4-(oct-7-en-1-yl)octahydro-2H-quinolizine	263951	286.249286	8.662066667	pos

1-Methylguanine	3778	166.072754	1.090316667	pos
1-methylhistidine	3741	337.164201	8.86655	neg
1-Methylnicotinamide	HMDB0000699	273.135127	7.20205	neg
1-Methylpyrrolo[1,2-a]pyrazine	89048	150.102859	2.012516667	pos
1-Methyluric acid	HMDB0003099	181.036308	1.112466667	neg
1-Naphthaleneacetic acid sodium salt	72340	415.094364	6.885783333	neg
1-Naphthalenesulfonic acid	71143	207.012048	5.221616667	neg
1-Naphthylacetylspermine	69627	393.262036	10.93775	pos
1-Nitrohexane	HMDB0013812	150.136034	0.002433333	pos
1-O-(2-methoxy-hexadecyl)-sn-glycerol	LMGL01020037	364.342745	12.43988333	pos
1-O-(2R-hydroxy-docosanyl)-sn-glycerol	LMGL01020068	831.76262	15.35605	neg
1-O-(2R-hydroxy-hexadecyl)-sn-glycerol	LMGL01020063	371.2566	12.41971667	pos
1-O-(2R-hydroxy-pentadecyl)-sn-glycerol	LMGL01020062	341.266872	12.43988333	pos
1-O-(2R-hydroxy-tetradecyl)-sn-glycerol	LMGL01020061	305.269376	11.99565	pos
1-O-(8R-hydroxy-8-methyl-3Z,9-decadienoyl)-beta-D	98324	399.139946	7.618183333	pos
1-O-Caffeoyl-(b-D-glucose 6-O-sulfate)	HMDB0041173	403.032988	8.5924	neg
1-Octen-3-yl glucoside	88858	308.207158	7.331416667	pos
1-Octen-3-yl primeveroside	88859	405.210116	1.090316667	pos
1-Octene	88470	130.159353	14.8265	pos
1-Octylglycerol	46620	243.136037	15.98305	pos
1-O-E-Cinnamoyl-(6-arabinosylglucose)	HMDB0030294	441.141202	5.348033333	neg
1-O-Galloylglycerol	HMDB0039177	487.110407	9.62565	neg
1-Oleoyl-2-acetyl-sn-glycerol	424	397.296661	11.15198333	neg
1-O-tricosanoyl-Cer(d18:1/16:0)	LMSP02040010	854.831889	0.6357	neg
1-Pentacosene	97734	368.425846	13.13813333	pos
1-Pentenyl glucosinolate	91498	386.058438	4.927216667	neg
1-Phenyl-1-pentanone	HMDB0031208	145.101481	0.526866667	pos
1-Phenyl-2-(diethylamino)-1-propanol	2014	225.19661	8.539533333	pos
1-PHENYLBIGUANIDE	44226	178.109149	15.98305	pos
1-Phosphatidyl-D-myo-inositol	HMDB0006953	408.090981	0.749333333	pos
1-Propenyl 1-(1-propenylthio)propyl disulfide	HMDB0038967	238.074183	16.00321667	pos
1-Thiocyanato-4-(methylthio)butane	HMDB0038446	142.015811	4.927216667	neg
1-Tridecene	HMDB0030930	200.237701	6.20565	pos
2-(1,2-Diamino-1-propenyl)phenol	90288	327.181963	7.875283333	neg
2-(14,15-Epoxyeicosatrienoyl) Glycerol	44863	417.262095	10.99823333	pos
2-(2-Thienylmethylene)-1,6-dioxaspiro[4.4]non-3-en	HMDB0034857	201.037806	1.09225	neg
2-(3,4-dihydroxy-5-methoxyphenyl)-3,5,7-trihydroxy-	HMDB0131422	397.032075	2.155183333	neg
2-(3-hydroxyphytanyl)-3-phytanyl-sn-glycerol	LMGL02030034	691.657195	14.35173333	pos
2-(3'-Methylthio)propylmalic acid	64493	223.064116	1.907233333	pos
2-(3-Phenylpropyl)pyridine	91322	198.128193	5.301516667	pos
2-(3-pyridyl)-4-methyl-Thiazole-5-Carboxylic Acid	45436	439.052449	4.927216667	neg
2-(3-pyridyl)-Benzimidazole	45439	213.113234	4.257983333	pos
2-(4-Methyl-5-thiazolyl)ethyl formate	88444	341.062517	8.5924	neg
2-(5,8-Tetradecadienyl)cyclobutanone	HMDB0037519	280.264035	12.33913333	pos
2-(9R-(15Z-docosenoyloxy)-3-methyl-2Z-decenoylo	97367	646.473934	10.89728333	pos
2-(Arabinosylamino)-3-(glucosylamino)propanenitrile	HMDB0039505	378.151803	7.518333333	neg
2-(Fluoromethoxy)-1,1,3,3,3-pentafluoro-1-propene	2424	224.999451	15.92651667	neg
2-(Formamido)-N1-(5-phospho-D-ribosyl)acetamidin	HMDB0006211	266.018444	5.411316667	neg
2-(Methylthiomethyl)-3-phenyl-2-propenal	92134	383.115281	1.09225	neg
2,12alpha-Dihydroxy-3-oxo-5beta-chol-1-en-24-oic A	43008	427.246457	12.86038333	pos
2,15,16-trihydroxy palmitic acid	35476	327.21479	11.71338333	pos
2,2,4,4,6,6-Hexamethyl-1,3,5-trithiane	94774	223.064064	0.463416667	pos
2,2,4,4,-Tetramethyl-6-(1-oxopropyl)-1,3,5-cyclohex	89065	261.110352	8.109516667	pos
2,2,4,4-Tetramethyl-6-(1-oxobutyl)-1,3,5-cyclohexan	HMDB0033197	235.133444	10.12496667	pos
2,2,6,6-Tetramethyl-4-piperidinone	HMDB0031179	200.129033	5.516666667	neg

2,2,6,7-Tetramethylbicyclo[4.3.0]nona-1(9),4-diene-7	HMDB0036686	209.154207	11.21971667	pos
2,2,7,7-Tetramethyl-1,6-dioxaspiro[4.4]nona-3,8-die	86659	181.122619	0.548016667	pos
2,2-Dimethyl-3,4-bis(4-methoxyphenyl)-2H-1-benzop	70521	475.1758	15.35605	neg
2,3,4,5-Tetrahydroxypentanoic acid	HMDB0059750	147.029131	2.05105	neg
2,3,5,7,9-Pentathiadecane 2,2-dioxide	HMDB0039734	262.936363	1.799533333	neg
2,3,5-Trimethyl-6-[4-(methylthio)butyl]pyrazine	HMDB0040005	269.131921	9.16185	neg
2,3,6-Trimethylphenol	HMDB0029667	154.122943	11.17941667	pos
2,3-Butanediol apiosylglucoside	88955	383.154333	6.12755	neg
2,3-di-0-hexanoyl-alpha-glucopyranose	53893	357.192647	8.022933333	neg
2,3-Diacetoxypropyl stearate	HMDB0059931	443.335414	12.52048333	pos
2,3-Dihydro-6-methoxy-2,2-dimethyl-4H-1-benzopyra	HMDB0041410	251.092913	8.5924	neg
2,3-dihydroxy stearic acid	35462	299.258766	10.73525	pos
2,3-Dihydroxy-2'-carboxybiphenyl	71164	231.065025	0.712516667	pos
2,3-Dihydroxytoluene	65862	247.098033	8.697816667	neg
2,3-dinor Fluprostenol	45699	859.309618	0.6532	neg
2,3-dinor Thromboxane B1	44923	327.217122	10.08378333	pos
2,3-dinor, 6-keto-PGF1alpha	36156	341.197575	7.790966667	neg
2,3-Dinor-8-iso-PGF2alpha	LMFA03110010	325.202853	9.73105	neg
2,3-Dinor-TXB1	45969	360.275083	10.10433333	pos
2,3-Dinor-TXB2	36258	341.197562	9.204033333	neg
2,3-Di-O-methylellagic acid	95796	311.020525	6.590883333	neg
2,3-Epoxymenaquinone	HMDB0060359	369.169719	14.01428333	neg
2,4,6,8,10-dodecapentaenal	75350	219.102735	8.52915	neg
2,4,6-Triaminotoluene	71188	155.129405	14.29941667	pos
2,4,6-trimethyl-3,5-dinitrobenzonitrile	34473	469.110967	11.56056667	neg
2,4-Diamino-6-nitrotoluene	HMDB0060362	333.13026	8.950933333	neg
2,4-Dichlorobenzoate	66483	188.95123	7.833116667	neg
2,4-dihydroxy-2-heptenedioc acid	LMFA01170062	189.040202	1.318283333	neg
2,4-dihydroxy-3-(sulfooxy)benzoic acid	HMDB0130471	294.97766	4.779683333	neg
2,4-Dihydroxy-6,7-dimethoxy-2H-1,4-benzoxazin-3(4	HMDB0037548	481.107927	10.88373333	neg
2',4'-Dimethylacetophenone	HMDB0032140	193.086695	8.697816667	neg
2,4-DINITROPHENOL	43994	183.004306	7.054383333	neg
2,4-Nonadien-1-ol	LMFA05000608	158.154263	8.820583333	pos
2,4-octadienal	36554	169.086486	7.980733333	neg
2,5-Diamino-6-(5-phospho-D-ribitylamino)pyrimidin-4	72671	336.070997	8.001833333	neg
2,5-Diethyl-3,6-dimethylpyrazine	94520	327.254927	11.0283	neg
2,5-Diethylpyrazine	HMDB0036808	271.192198	9.62565	neg
2,5-Dihydroxybenzaldehyde	44699	139.03932	3.827083333	pos
2,5-Dimethyl-3-propylpyrazine	87963	299.223357	11.31588333	neg
2,5-Dimethylbenzaldehyde	265044	135.080811	8.921266667	pos
2,6-Diamino-4-hydroxy-5-N-methylformamidopyrimid	HMDB0011657	182.067843	13.7265	neg
2,6-Diethylpyrazine	91824	271.192211	8.318183333	neg
2',6'-Dihydroxy-4'-methoxy-3'-(2-hydroxybenzyl)dihyd	LMPK12120480	417.109035	7.4543	pos
2,6-Dimethoxy-4-propylphenol	91372	197.117667	1.167366667	pos
2,6-Dimethoxyphenol	68578	307.119444	7.054383333	neg
2,6-Dimethyl-6-O-beta-D-quinovopyranosyl-7-octade	LMFA13010036	331.177082	6.14865	neg
2,6-Dimethyl-7-octene-1,6-diol 8-O-glucoside	89085	357.189015	8.191433333	pos
2,6-Dimethyl-7-octene-2,3,6-triol	LMFA05000610	169.122864	9.520516667	neg
2,8-Dihydroxyquinoline-beta-D-glucuronide	62403	336.07097	8.5924	neg
2,8-Di-O-methylellagic acid	95797	311.020596	15.98875	neg
2-[3-Carboxy-3-(methylammonio)propyl]-L-histidine	3400	271.139145	5.65175	pos
2-{[hydroxy({3,4,8,9,10-pentahydroxy-6-oxo-6H-benz	HMDB0126665	422.035396	4.927216667	neg
2-{[hydroxy(2,3,4-trimethoxyphenyl)methylidene]ami	HMDB0142117	314.089026	8.613483333	neg
2-{[hydroxy(4-methoxy-1-benzofuran-5-yl)methylider	HMDB0129408	294.062867	6.696183333	neg
2-{[hydroxy(6-hydroxy-7-methoxy-2H-1,3-benzodioxo	HMDB0128732	537.098815	11.58091667	neg

20:0-Glc-Sitosterol	LMST01040208	888.765038	4.179616667	pos
20-carboxy-LTB4	36247	365.195368	10.08896667	neg
20-hydroxy LTB4	36249	353.231597	10.22716667	pos
20-hydroxy-PGF2a	3821	371.24388	9.878016667	pos
20-oxo-heneicosanoic acid	74774	358.332176	10.75555	pos
21-Fluoro-11beta-hydroxypregn-4-ene-3,20-dione	70591	393.20805	11.3567	neg
21-Hydroxy-5b-pregnane-3,11,20-trione	57883	385.179085	15.32708333	pos
22:1-Glc-Stigmasterol	LMST01040234	912.765153	0.658466667	pos
24,24-difluoro-1alpha,25-dihydroxy-24a-homovitami	42251	489.315494	5.796033333	pos
24-Nor-5beta-cholane-3alpha,7alpha,12alpha,22,23	84906	419.277661	12.0361	pos
24-Nor-5beta-cholane-3alpha,7alpha,12alpha,23-tet	LMST04060003	403.28294	12.33913333	pos
25-hydroxyvitamin D3	LMST03020246	423.324558	10.02156667	pos
26,26,26-trifluoro-25-hydroxyvitamin D3	LMST03020131	437.302273	11.33671667	pos
27-Nor-5b-cholestane-3a,7a,12a,24,25-pentol	HMDB0002126	439.340433	15.05798333	pos
27-Norcholestanehexol	HMDB0002157	455.335384	13.94848333	pos
2-Acetyl-3,6-dimethylpyrazine	94539	299.150662	6.401333333	neg
2-Acetyl-3-ethylpyrazine	86250	299.150672	6.085383333	neg
2-Acetyl-3-methylpyrazine	86654	271.119415	6.211833333	neg
2-Acetyl-4,5-dihydrothiazole	HMDB0033561	174.022525	2.09275	neg
2-Acetylpyrazine	HMDB0031861	243.08776	4.674333333	neg
2alpha-(3-Hydroxypropyl)-1alpha,25-dihydroxy-19-n	42553	485.361324	13.72018333	pos
2-Amino-1,7,9-trimethylimidazo[4,5-g]quinoxaline	HMDB0040300	228.12483	4.160016667	pos
2-Amino-1-methyl-6-phenylimidazo[4,5-b]pyridine	71126	247.09448	15.94263333	pos
2-Amino-3,4,8-trimethyl-3H-imidazo[4,5-f]quinoxalin	HMDB0032928	228.124847	4.484716667	pos
2-Amino-3-methylimidazo[4,5-f]quinoline	72911	199.096978	0.548016667	pos
2-amino-4-({2-[(1-carboxy-2-hydroxy-1-methyl-2-phe	HMDB0133670	969.282716	14.48523333	neg
2-Amino-4-nitrotoluene	71186	135.055658	15.67405	pos
2-Aminomuconate 6-semialdehyde	3248	142.0502	1.718916667	pos
2-Aminomuconic acid semialdehyde	HMDB0001280	142.050201	1.007533333	pos
2-Benzimidazolylguanidine	68668	176.093542	15.98305	pos
2-Benzothiazolesulfonamide	69856	212.97912	5.2427	neg
2-Butanone, 4-[6-(sulfooxy)-2-naphthalenyl]-	1378	293.049614	0.6902	neg
2-Carboxy-4-dodecanolide	87331	260.186167	5.260266667	pos
2-Chlorophenylhydrazine hydrochloride	69110	199.988324	0.566216667	pos
2-C-Methyl-D-erythritol 2,4-cyclodiphosphate	64016	322.994434	9.9417	neg
2-C-Methyl-D-erythritol 4-phosphate	64013	215.03262	0.74595	neg
2-decene-4,6,8-triyn-1-al	75341	125.038397	0.584866667	pos
2-Dehydro-D-xylonate	65865	145.01346	1.09225	neg
2'-Deoxy-5-hydroxymethylcytidine-5'-triphosphate	63416	541.995885	4.211566667	neg
2'-Deoxymugineic acid	70941	349.125307	7.32855	neg
2-Dimethylamino-5,6-dimethylpyrimidin-4-ol	68685	333.20545	10.9664	neg
2-dimethylarsinoyl-acetic acid	LMFA00000035	224.975107	4.716466667	neg
2-Dodecenal	HMDB0031020	165.164143	0.42115	pos
2-Dodecylbenzenesulfonic acid	HMDB0031031	325.184923	14.44851667	neg
2-DPMP	85086	269.201957	6.633866667	pos
2E,4E-undecadienoic acid	34888	205.120168	15.98305	pos
2E,6E,8E,10E-dodecatetraenoic acid	34906	193.122829	10.43013333	pos
2E,6Z,8Z,12E-hexadecatetraenoic acid	LMFA01030277	249.18539	11.25935	pos
2E,6Z-nonadienoic acid	74105	137.096456	0.548016667	pos
2E,7E-decadienoic acid	34868	335.221114	11.23385	neg
2E,7Z-decadienoic acid	LMFA01030203	213.113238	8.2549	neg
2E,8E-dodecadienoic acid	LMFA01030232	214.180598	0.548016667	pos
2E,8Z-dodecadienoic acid	LMFA01030233	241.144866	9.583833333	neg
2E-Decenedioic acid	74917	245.103395	4.842916667	neg
2-ene-Valproic acid	HMDB0013902	283.192204	10.1945	neg

2-Ethoxy-1-methyl-4-(1-methylethyl)benzene	92224	161.132759	0.002433333	pos
2-Ethoxy-3-methylpyrazine	HMDB0031850	275.15085	6.590883333	neg
2-Ethoxy-5-(1-propenyl)phenol	95265	179.107061	9.71345	pos
2-Fluorobenzoate	65752	178.991107	11.81406667	pos
2-Furoic acid	HMDB0000617	223.024992	5.263766667	neg
2-glyceryl-PGD2	36230	425.255657	10.32093333	neg
2-Hexylglycerol	LMGL01020029	194.175577	5.898866667	pos
2-hydroxy behenic	LMFA01050077	379.318789	9.0643	pos
2-hydroxy pelargonic acid	35406	155.107045	7.49725	neg
2-Hydroxy-2H-benzo[h]chromene-2-carboxylate	69029	241.051013	7.138766667	neg
2-Hydroxy-3-methoxyestrone	HMDB0011195	480.309442	10.55303333	pos
2-hydroxy-4- (methylthio) butyric acid Calcium salt	44706	321.014896	0.69355	pos
2-Hydroxy-6-keto-2,4-heptadienoate	66356	139.039322	1.949316667	pos
2-Hydroxyadenine	5392	152.056974	2.11315	pos
2-Hydroxycinnamic acid	306	182.081615	1.2291	pos
2'-Hydroxygenistein	47834	285.041226	4.927216667	neg
2-Hydroxyhexadecanoic acid	HMDB0031057	290.269556	5.7548	pos
2-hydroxy-iminostilbene	LMPK13090031	192.08034	0.641016667	pos
2-Hydroxymuconate semialdehyde	65525	180.990205	0.584866667	pos
2-Hydroxymyristic acid	LMFA01050484	245.211596	0.002433333	pos
2-Hydroxypropylphosphonate	63585	185.021656	0.823166667	neg
2-Isopropyl-5-methoxypyrazine	94593	170.129178	10.89728333	pos
2-Isopropyl-5-methylpyrazine	HMDB0037151	271.192186	9.14075	neg
2-Isopropylmalic acid	HMDB0000402	159.065531	1.907233333	pos
2-keto tridecanoic acid	LMFA01060041	273.171551	7.054383333	neg
2-Keto-3-deoxy-D-gluconic acid	LMFA01050486	159.029189	2.09275	neg
2-keto-n-caprylic acid	LMFA01060016	203.092308	4.948316667	neg
2-Keto-n-heptylic acid	LMFA01060011	287.15073	6.61195	neg
2-Methoxy-(3 or 5 or 6)-isopropylpyrazine	88408	303.182056	6.927933333	neg
2-Methoxy-3-(1-methylpropyl)pyrazine	88836	331.213393	8.044016667	neg
2-Methoxy-3,5-dimethylpyrimidine	95361	275.150845	6.380283333	neg
2-Methoxy-3-methyl-9H-carbazole	89287	210.092421	7.7066	neg
2-methoxy-6Z-hexadecenoic acid	35881	285.242938	11.61323333	pos
2-Methoxyacetaminophen sulfate	HMDB0062550	260.023954	1.09225	neg
2-Methoxy-estradiol-17b 3-glucuronide	HMDB0006765	477.214577	11.8654	neg
2-Methoxy-estradiol-17beta 3-glucuronide	LMST05010009	477.213959	11.41786667	neg
2-Methoxyestradiol-17beta 3-sulfate	LMST05020007	421.106336	10.73525	pos
2-Methoxyestrone 3-sulfate	LMST05020006	398.161441	8.900733333	pos
2-Methyl-3-hydroxy-5-formylpyridine-4-carboxylate	58601	164.03453	2.032866667	pos
2-methyl-4-oxo-pentadecanoic acid	LMFA01060104	271.227555	1.949316667	pos
2-Methyl-5-(2-propenyl)pyrazine	HMDB0039993	267.160906	9.857533333	neg
2-Methyl-5-vinylpyrazine	HMDB0037282	138.102981	15.98305	pos
2-Methylacetophenone	HMDB0032386	421.227602	14.50361667	neg
2-Methylaminoadenosine	65933	295.115295	4.211566667	neg
2-Methylbenzoic acid	6632	137.06003	0.002433333	pos
2-Methylbenzyl alcohol acetate	HMDB0041472	209.081925	7.601116667	neg
2-methyl-dodecanedioic acid	74902	289.166432	6.5277	neg
2-Methylpropyl glucosinolate	HMDB0038421	356.04789	4.211566667	neg
2-Methylpropyl hexanoate	LMFA07010729	173.153952	0.505683333	pos
2-N,6-N-Bis(2,3-dihydroxybenzoyl)-L-lysine amide	66404	833.303783	0.6532	neg
2-Naphthalenepropanol, 6-methoxy-a-methyl-, hydro	1379	309.079899	0.67025	neg
2-n-Propyl-4-oxopentanoic acid	HMDB0060683	315.181919	8.845466667	neg
2-O-(6-Phospho-alpha-mannosyl)-D-glycerate	HMDB0012152	366.080261	0.712516667	pos
2-O-beta-D-Glucopyranuronosyl-D-mannose	HMDB0039722	379.08441	1.970383333	pos
2'-O-Methyladenosine	58235	282.120251	1.090316667	pos

2'-O-Methylcajanone	LMPK12050497	437.194349	15.25505	pos
2'-O-Methylphaseollinisoflavan	HMDB0034412	377.113888	4.90555	pos
2-oxo capric acid	35701	371.244584	10.8423	neg
2-Oxo-4-methylthiobutanoic acid	6319	166.053425	0.749333333	pos
2-oxo-5-amino-pentanoic acid	35853	132.065884	0.7301	pos
2-oxo-docosanoic acid	LMFA01060140	399.312217	11.96663333	neg
2-oxo-heneicosanoic acid	74773	358.332147	10.63491667	pos
2-oxo-nonadecanoic acid	74768	357.265614	9.9417	neg
2-Oxosuberate	71247	206.102775	2.052666667	pos
2-Oxoticlopidine	HMDB0013923	297.082815	1.907233333	pos
2-Pentadecanone	87393	244.263919	9.509383333	pos
2-Pentadecylfuran	89411	261.258532	0.42115	pos
2-Phenyl-1,3-propanediol monocarbamate	HMDB0060351	176.071203	5.053716667	neg
2-Phenyl-4-pentenal	HMDB0035207	205.086907	8.613483333	neg
2-Phenylacetamide	HMDB0010715	180.06616	1.235383333	neg
2-Phenylethanol glucuronide	HMDB0010350	281.102435	9.491233333	pos
2-Phenylethyl 3-phenyl-2-propenoate	HMDB0035018	251.107989	11.72336667	neg
2-Phenylethyl beta-D-glucopyranoside	86524	267.123224	9.473266667	pos
2-Phenylpropionaldehyde dimethyl acetal	88484	163.112073	1.886166667	pos
2-Piperidinecarboxamide, N-(3-hydroxy-2,6-dimethy	2384	249.160307	5.23965	pos
2-Polyprenyl-6-methoxyphenol	HMDB0060355	259.170866	11.45865	neg
2-Propenyl 2,4-hexadienoate	HMDB0036227	153.091318	0.548016667	pos
2-Propenyl cyclohexanebutanoate	LMFA07010743	211.169684	0.548016667	pos
2-Propenyl cyclohexanehexanoate	92162	256.227598	14.6315	pos
2-Propenyl cyclohexanepentanoate	92625	269.176579	9.77325	neg
2-Propenyl heptanoate	HMDB0040208	169.12285	8.381466667	neg
2-Propenyl hexanoate	HMDB0040210	157.122658	0.002433333	pos
2-Propylglutaric acid	2998	157.086328	1.048966667	pos
2R-hydroxy-10-undecenoic acid	35681	245.139801	5.57985	neg
2S-amino-3-oxo-butanoic acid	LMFA01060172	233.07801	0.802716667	neg
2-Stearyl citrate	HMDB0039225	427.306321	10.87708333	pos
2-Undecenal	HMDB0040247	186.185685	9.7543	pos
2Z,6Z,8Z,12E-hexadecatetraenoic acid	34943	287.141006	8.27325	pos
2Z,8E-dodecadienoic acid	73909	214.18049	13.52618333	pos
2Z,8Z-dodecadienoic acid	34900	241.144839	8.086216667	neg
2Z-Dodecenedioic acid	LMFA01170033	273.135146	5.411316667	neg
3-((3R,5S)-3-butyloctahydroindolizin-5-yl)propan-1-c	263837	284.223807	9.836516667	neg
3-(1,1-Dimethyl-2-propenyl)-8-(3-methyl-2-butenyl)x	87156	727.401271	4.021883333	neg
3-(1-Hydroxymethyl-1-propenyl)pentanedioic acid	88984	403.158986	13.0037	neg
3-(2-Methylpropylidene)-3alpha,4-dihydro-1(3H)-isol	HMDB0032027	173.096505	0.002433333	pos
3-(3,4-Dihydroxy-5-methoxy)-2-propenoic acid	HMDB0035484	209.045406	5.13805	neg
3-(3,5-Diiodo-4-hydroxyphenyl)lactate	HMDB0059636	456.839602	14.66688333	pos
3-(3-hydroxyphenyl)-2-phenyl-4-[(E)-2-phenyletheny	HMDB0129161	445.120964	1.9283	pos
3-(3-Hydroxyphenyl)propanoic acid	HMDB0000375	167.070652	1.028233333	pos
3-(but-3-en-1-yn-1-yl)-6-methyl-1,2-dithiine	LMFA12000365	181.01481	11.51225	pos
3-(Hydrohydroxyphosphoryl)pyruvate	63597	174.976975	0.905683333	pos
3,11,12-Trihydroxy-1(10)-spirovetiven-2-one	92895	313.166527	6.717266667	neg
3,12-dihydroxy palmitic acid	35460	311.220034	12.3592	pos
3,3-dichloro-propionic acid	LMFA01090067	164.948461	1.048966667	pos
3,3'-Diiodothyronine	HMDB0005869	542.928888	13.63223333	pos
3,4,5-trihydroxy-6-[(3-methoxy-3-oxopropanoyl)oxy]c	HMDB0130026	339.057601	4.169433333	neg
3,4,5-trihydroxy-6-[(4,5,6-trihydroxy-2-{[(3-methoxy-3	HMDB0130022	455.102975	0.823166667	neg
3,4,5-trihydroxy-6-[(4-hydroxy-7-methoxy-2,2-dimeth	HMDB0125816	399.13044	4.82185	neg
3,4,5-trihydroxy-6-[4-hydroxy-5-(methoxymethyl)-2-(	HMDB0127854	555.134334	5.348033333	neg
3,4,5-trihydroxy-6-{[3,5,7-trihydroxy-4-oxo-2-(3,4,5-tr	HMDB0125240	491.048352	5.7905	neg

3,4,5-trihydroxy-6-{[5-hydroxy-2-(4-hydroxyphenyl)-8	HMDB0127397	567.090247	4.118683333	pos
3,4-Dihydro-4-[(5-methyl-2-furanyl)methylene]-2H-py	HMDB0040048	200.047711	5.7548	pos
3,4-Dihydro-6-methoxy-2,2-dimethyl-2H-1-benzopyra	95810	253.108576	8.5924	neg
3,4-dihydroxy-5-(sulfooxy)cyclohex-1-ene-1-carboxy	HMDB0130162	507.009949	11.3567	neg
3,4-Dihydroxymandelaldehyde	HMDB0006242	149.023639	5.348033333	neg
3,4-Methyleneazelaic acid	HMDB0059744	257.103645	4.990483333	neg
3,5,7,9,11-dodecapentaenoic acid	34908	235.097833	8.360383333	neg
3,5,7-Trimethyl-2E,4E,6E,8E-decatetraene	97469	221.154726	11.17246667	neg
3,5-decadienoic acid	LMFA01030206	213.113209	6.927933333	neg
3,5-Diiodothyronine	HMDB0000582	542.928759	13.9834	pos
3,5-DINITROCATECHOL (OR-486)	44188	180.988648	6.0222	neg
3,5-Dinitro-L-tyrosine	65936	316.041925	1.297583333	neg
3,5-Dinitrosalicylic acid	68986	210.998268	4.7158	pos
3,5-Di-O-methyl-8-prenylafzelechin-4beta-ol	47432	409.163008	9.473266667	pos
3,6,7-Trihydroxy-4'-methoxyflavone 7-rhamnoside	50004	491.120693	7.833116667	neg
3,6,8-dodecatrien-1-ol	36515	181.15902	0.002433333	pos
3,6-dioxo-decanoic acid	74717	245.10338	5.36915	neg
3,6-Ditigloyloxytropan-7-ol	86189	673.368234	13.7265	neg
3,7,11-Trimethyldodeca-2E,4E-diene	LMFA11000045	226.25334	7.925333333	pos
3,7,8,15-Scirpenetetrol	92417	281.138862	10.22716667	pos
3,7-Dihydroxy-2-[3-(4-hydroxy-3-methylbutyl)-4-meth	51270	859.314057	14.0863	neg
3,7-Dimethyl-2E,6E-decadien-1,10-dioic acid	96875	227.128096	4.337966667	pos
3,7-Dimethyl-8,11-dioxo-2E,6E,9E-dodecatrienal	46446	235.133293	9.632383333	pos
3-[(5-Methyl-2-furanyl)methyl]-1H-pyrrole	94583	200.047616	5.363416667	pos
3-[4-Hydroxy-3-(3-methyl-2-butenyl)phenyl]-2-proper	HMDB0040833	261.11391	9.60495	neg
3a,11b,21-Trihydroxy-20-oxo-5b-pregnan-18-al	57884	363.216177	10.46825	neg
3a,21-Dihydroxy-5b-pregnane-11,20-dione	HMDB0006755	349.236994	9.91855	pos
3-Acrylamidopropyl trimethylammonium	34500	171.149523	6.348883333	pos
3alpha,6alpha,12alpha-Trihydroxy-7-oxo-5beta-chol	LMST04010274	423.273422	10.6551	pos
3alpha,6alpha,7alpha,12alpha-Tetrahydroxy-5beta-c	42710	425.289932	10.59398333	pos
3alpha,6beta,7beta,12beta-Tetrahydroxy-5beta-chol	LMST04010123	469.281684	10.1312	neg
3Alpha,7Alpha,12Alpha-trihydroxy-5Beta-cholestan-	LMST04030001	495.333775	10.98706667	neg
3alpha,7alpha,12beta-Trihydroxy-11-oxo-5beta-chol	LMST04010383	423.275896	5.589866667	pos
3alpha,7alpha-Dihydroxy-22-oxo-5beta-cholan-24-oi	LMST04010439	451.268978	11.3567	neg
3alpha,7beta,12alpha-Trihydroxy-6-oxo-5alpha-chol	42845	405.262578	11.47181667	pos
3-alpha-hydroxy-5-alpha-androstane-17-one 3-D-glu	LMST05010061	447.236535	10.27876667	neg
3alpha-Hydroxy-6-oxo-5beta-cholan-24-oic Acid	42735	429.241363	16.00321667	pos
3-amino-2-naphthoic acid	34509	205.097459	4.160016667	pos
3beta-Acetoxy-19alpha-hydroxy-12-ursene	91546	523.356387	9.733716667	pos
3beta-Acetoxy-4-oplopanone	53436	341.197458	8.697816667	neg
3beta-Hydroxy-6-oxo-5beta-cholan-24-oic Acid	42736	413.267063	0.641016667	pos
3b-Hydroxy-6b-methoxy-7(11)-eremophilen-12,8a-ol	95220	325.166295	8.5924	neg
3-butyrl propionic acid	LMFA01060013	287.150721	5.874783333	neg
3-caproyl propionic acid	35718	343.213344	9.478266667	neg
3-Carboxy-4-methyl-2-oxopentanoate	3301	173.04621	13.54503333	neg
3-carboxy-4-methyl-5-pentyl-2-furanpropanoic acid	LMFA01150047	251.128306	9.672883333	pos
3-Chlorobenzoic acid	6311	154.989836	7.2653	neg
3'-CMP	63414	322.045347	1.09225	neg
3-Deazaneplanocin A	64841	261.098605	4.6322	neg
3-Dehydrosphinganine	HMDB0001480	282.279588	11.1996	pos
3-Dehydroteasterone	64147	447.347719	0.045716667	pos
3'-Deoxydryopteric acid	47250	377.086393	0.802716667	neg
3'-Deoxymaysin	48680	541.136449	0.884666667	neg
3'-Deoxystreptomycin 3''-phosphate	2512	684.202763	15.49035	pos
3E,5E-tridecadienoic acid	LMFA01030244	193.159227	10.95795	pos

3E-hexadecenoic acid	34930	237.221601	0.505683333	pos
3-Ethyl-1,2-benzenediol	HMDB0040177	275.129647	9.60495	neg
3-Ethyl-5-methylphenol	HMDB0038985	181.086627	6.548766667	neg
3-Ethylcatechol	66508	139.075745	0.548016667	pos
3-Ethyltridecan-2-one	98249	244.263983	9.268416667	pos
3-Hexadecanoyloleanolic acid	91955	695.597323	4.337966667	pos
3-hexanoyl-NBD Cholesterol	64806	663.450903	13.23493333	pos
3-Hexaprenyl-4-hydroxy-5-methoxybenzoic acid	5915	599.409519	15.98305	pos
3-hexenal	75293	195.138775	10.0258	neg
3-Hydroxy-2,8-dimethyl-1,7-dioxaspiro[5.5]undecane	LMPK09000029	245.139816	6.5277	neg
3-Hydroxy-4-aminopyridine sulfate	HMDB0061120	228.967304	1.028233333	pos
3-Hydroxy-6,8-dimethoxy-7(11)-eremophilen-12,8-ol	HMDB0040756	309.17133	9.393866667	neg
3-Hydroxy-7-acetamidoclonazepam	1888	326.067407	5.280883333	pos
3-Hydroxy-beta-ionone	HMDB0036821	191.143439	10.14556667	pos
3-Hydroxycarbamazepine	HMDB0060653	235.085838	4.942016667	pos
3-hydroxydecanoyl carnitine	HMDB0061636	332.24371	8.982766667	pos
3-Hydroxydodecanedioic acid	5402	229.1439	1.007533333	pos
3-hydroxy-hexadecanoic acid	LMFA01050188	290.269593	7.351916667	pos
3-Hydroxylidocaine	HMDB0060655	251.175809	5.301516667	pos
3-Hydroxymonoethylglycinexylidide	HMDB0060678	443.266071	10.0258	neg
3-Hydroxymugineic acid	HMDB0033927	381.114068	11.2953	neg
3-Hydroxynonyl acetate	HMDB0032443	183.138624	8.613483333	neg
3-hydroxyoctanoyl carnitine	HMDB0061634	304.212283	7.495216667	pos
3-Hydroxyphenylacetic acid	4150	135.044404	0.002433333	pos
3-hydroxy-sebacic acid	45926	241.105015	5.589866667	pos
3-hydroxy-tetradecanoic acid	35541	262.238298	5.918766667	pos
3-hydroxytridecanoyl carnitine	HMDB0061639	374.290895	9.7543	pos
3-Hydroxyvalproic acid	HMDB0013899	143.106992	0.002433333	pos
3-iodo-hexadecanoic acid	LMFA01090038	363.12073	4.737533333	neg
3-Isopropylbut-3-enoyl-CoA	LMFA07050231	895.220394	4.7158	pos
3-keto-n-caprylic acid	35712	203.092325	6.14865	neg
3-ketosphinganine	3428	300.290229	12.39953333	pos
3-ketosphingosine	43208	320.256631	11.57286667	pos
3-methoxy Limaprost	45111	413.291809	4.65325	pos
3-Methoxy-4-hydroxyphenylglycolaldehyde	HMDB0004061	163.039433	5.621966667	neg
3'-Methoxyfukiic acid	86308	267.051738	5.453416667	neg
3-Methoxymorphinan	85336	296.140159	5.280883333	pos
3-Methoxytyramine	HMDB0000022	185.128867	4.014216667	pos
3-methyl-1-heptene	97970	130.159414	4.673916667	pos
3-Methyl-5-pentyl-2-furanundecanoic acid	87343	337.27411	10.6551	pos
3-Methylbutyl dodecanoate	92294	271.263685	15.84153333	pos
3-Methylbutyl nonanoate	HMDB0037495	267.172608	11.09911667	pos
3-Methylcyclohexanethiol	87746	259.155676	6.106483333	neg
3-Methyldioxyindole	7024	164.070905	0.946233333	pos
3-Methylhistamine	HMDB0001861	126.103004	14.40406667	pos
3-Methylhistidine	HMDB0000479	214.083374	11.43826667	neg
3-Methylindolepyruvate	63554	200.071044	5.507416667	pos
3-Methyl-L-histidine	3293	337.164366	9.794333333	neg
3-methyl-tetradecanedioic acid	LMFA01170019	255.19595	8.314233333	pos
3-Methylxanthine	HMDB0001886	167.055846	1.991466667	pos
3-Nonanon-1-yl acetate	HMDB0037179	399.276102	10.6567	neg
3-O-Acetylepisamarcandin	HMDB0031958	460.270509	9.85735	pos
3-O-Benzyl-4,5-O-(1-methylethyldiene)-b-D-fructopy	85019	333.131542	6.839233333	pos
3'-O-Methyl-(-)-epicatechin-7-O-sulphate	86065	385.08255	9.1045	pos
3-O-Methylrimiterol glucuronide	2337	458.168065	9.457166667	neg

3-O-Methylrimiterol sulfate	2339	340.08124	4.632166667	pos
3-O-trans-Feruloyleuscaphic acid	HMDB0031837	709.397229	13.78076667	neg
3-oxo capric acid	LMFA01060028	231.123996	6.885783333	neg
3-oxo-pentadecanoic acid	74741	301.202748	8.2549	neg
3-Oxovalproic acid	2990	315.181921	7.622216667	neg
3-Piperidinemethanol, 4-(4-fluorophenyl)-, (3S,4R)-	1718	227.15468	5.979883333	pos
3-propylmalic acid	LMFA01170064	159.065519	1.048966667	pos
3R-hydroxy-5Z-dodecenoic acid	35609	259.155686	6.822583333	neg
3R-hydroxy-octadecanoic acid	74648	318.300962	9.939183333	pos
3R-hydroxy-pentadecanoic acid	35672	259.227469	1.907233333	pos
3S-methyl-2-oxo-pentanoic acid	LMFA01020275	259.119283	6.970066667	neg
3-Succinoylpyridine	HMDB0000992	224.056599	5.032616667	neg
3Z,5E-tetradecadienoic acid	73929	247.167267	15.92241667	pos
3Z,5E-tridecadienoic acid	73920	209.154646	9.077466667	neg
3Z,6Z,9Z,12Z,15Z-Tricosapentaene	LMFA11000180	332.331874	10.79605	pos
3Z-Hexenyl 2R-hydroxy-3-methylbutyrate	LMFA07010641	201.148922	1.949316667	pos
4-(3,4-Dihydroxyphenyl)-2-hydroxy-1H-phenalen-1-c	93043	287.070738	0.658466667	pos
4-(3-Pyridyl)-3-butenoic acid	6236	146.060402	0.548016667	pos
4-(beta-Acetylaminoethyl)imidazole	66292	305.172456	7.53855	neg
4-(Cytidine 5'-diphospho)-2-C-methyl-D-erythritol	64014	560.045546	10.36856667	pos
4-(Dimethylamino)azobenzene	HMDB0032141	243.15951	6.818716667	pos
4-(dimethylamino)azobenzene n-oxide	4085	259.154458	11.65356667	pos
4'-(DIMETHYLAMINO)AZOXYBENZENE N-OXIDE	24046	243.159626	8.068566667	pos
4-(Hydroxymethyl)benzenediazonium(1+)	88549	269.103751	6.633	neg
4-(Trimethylammonio)but-2-enoate	66118	161.128695	0.641016667	pos
4,11,13,15-Tetrahydroridentin B	91305	286.201639	5.796033333	pos
4,4alpha,5,6-Tetrahydro-7-methyl-2(3H)-naphthalen	HMDB0039803	207.102559	15.94736667	neg
4,4'-Sulfonyldiphenol	69883	251.037108	0.712516667	pos
4,4'-Thiobis-2-butanone	HMDB0037155	347.135524	5.326966667	neg
4,5-Dihydroniveusin A	HMDB0032844	435.141179	12.48021667	pos
4,5-Dihydrovomifoliol	95095	271.155861	7.580016667	neg
4,5-Dihydroxypyrene	72094	215.051403	8.297083333	neg
4,5-Di-O-methyl-8-prenylafzelechin-4beta-ol	LMPK12020217	425.136846	8.880233333	pos
4,7,10-hexadecatrienoic acid	LMFA01030134	273.182811	14.96886667	pos
4,8 dimethylnonanoyl carnitine	58362	374.25564	6.738333333	neg
4-[[5-(acetylamino)-1-(hydroxymethyl)-1H-indol-3-yl]	3060	566.15848	0.884666667	neg
4-[2-(Propylamino)ethyl]-1,3-dihydro-2H-indol-2-one	2366	219.14969	5.404333333	pos
4-Acetamido-2-aminobutanoic acid	HMDB0031411	143.081817	15.92241667	pos
4-Acetoxyscirpene-3,15-diol	91064	369.156205	5.853733333	neg
4-Acetyl-2-methylpyrimidine	92630	271.119417	5.621966667	neg
4-Acetylimidazo[4,5-c]pyridine	HMDB0034888	179.093089	0.002433333	pos
4alpha-carboxy-4beta-methyl-5alpha-cholesta-8-en-	HMDB0062384	445.368542	14.87983333	pos
4alpha-formyl-4beta-methyl-5alpha-cholesta-8,24-di	LMST01010229	427.35794	13.72018333	pos
4-Amino-2-hydroxylamino-6-nitrotoluene	71191	164.045838	12.78818333	neg
4-Aminobiphenyl	HMDB0013195	170.096742	0.002433333	pos
4-Butyl-2,5-dimethylthiazole	HMDB0040076	187.126752	7.904866667	pos
4-Chloro-3,5-dimethoxybenzaldehyde	95381	181.005732	4.211566667	neg
4-Chloro-5-sulfamoylanthranilic acid	HMDB0060761	248.973328	15.20393333	neg
4-chloro-alpha-Cyanocinnamic Acid	96557	206.00135	0.74595	neg
4-Chlorobiphenyl	66440	233.037527	5.7905	neg
4-Deacetylneosolaniol	91309	321.132714	9.077466667	neg
4E,14Z-Sphingadiene	53902	280.264089	10.89728333	pos
4-ene-Valproic acid	LMFA01030982	187.097195	5.7694	neg
4-Fluorobenzoate	65758	178.991107	5.692966667	pos
4-Fluorocatechol	71214	166.991142	7.10605	pos

4-fumarylacetoacetic acid	LMFA01170066	181.013807	4.337333333	neg
4-Guanidinobutanamide	3269	167.089889	2.895883333	pos
4-Guanidinobutanoic acid	HMDB0003464	128.082319	15.92241667	pos
4-hydroxy Nonenal Mercapturic Acid-d3	96541	303.145597	5.263766667	neg
4-hydroxy Nonenal-d3	96566	317.260688	10.73845	neg
4-Hydroxy-1-(3-pyridinyl)-1-butanone	HMDB0062402	329.151384	10.21556667	neg
4-Hydroxy-3-nitrosobenzamide	69313	149.034713	0.566216667	pos
4-Hydroxy-4-methyl-7-decenoic acid gamma-lactone	91340	183.138409	9.652616667	pos
4-Hydroxy-4-methylglutamate	63271	158.046485	12.84735	neg
4-Hydroxy-5-(3'-hydroxyphenyl)-valeric acid-3'-O-sul	HMDB0059975	313.03639	0.641016667	pos
4-Hydroxy-5-(dihydroxyphenyl)-valeric acid-O-methy	HMDB0059977	381.050932	8.5924	neg
4-Hydroxy-5-(dihydroxyphenyl)-valeric acid-O-sulpha	HMDB0059978	345.002662	1.127133333	pos
4-Hydroxybenzenesulfonic acid	34512	192.032937	1.090316667	pos
4-Hydroxycinnamyl aldehyde	44643	193.050332	7.307466667	neg
4-Hydroxydebrisoquine	58417	209.140217	15.98305	pos
4-hydroxyestradiol	41825	333.171083	10.3631	neg
4'-Hydroxyflurbiprofen	2924	305.082513	8.465833333	neg
4-hydroxynonenal	75330	311.223627	10.9664	neg
4-Hydroxyphenylpyruvic acid	3315	179.034505	5.36915	neg
4-Hydroxyphthalate	66491	163.00306	0.573266667	neg
4-Imidazolone-5-propionic acid	319	155.045457	13.7265	neg
4-keto lauric acid	LMFA01060039	427.307418	10.82155	neg
4'-Methoxymucidin	86667	271.13357	10.43013333	pos
4-Methyl-1-phenyl-2-pentanone	HMDB0031569	159.117133	0.526866667	pos
4-Methylcatechol	HMDB0000873	247.097985	8.065116667	neg
4-Methyldibenzothiophene	94701	197.042812	4.842916667	neg
4-methyl-dodecanedioic acid	LMFA01170012	227.164503	7.577283333	pos
4-Methylene-L-glutamine	63274	176.103345	0.749333333	pos
4-Methylpyrrolo[1,2-a]pyrazine	89050	150.102942	15.98305	pos
4-Methylumbelliferone	65904	177.055005	6.000516667	pos
4-Methylumbelliferyl sulfate	4083	274.038494	5.115883333	pos
4-n-valeryl butyric acid	35719	343.213373	9.857533333	neg
4-O-alpha-D-Glucopyranuronosyl-D-galactose	HMDB0039724	379.084426	0.566216667	pos
4'-O-Methylglabridin	48261	377.113912	5.3837	pos
4-O-Methylmelleolide	HMDB0037039	437.194335	9.85735	pos
4-oxo 2-Nonenal-d3	96377	158.126024	1.991466667	pos
4-oxo capric acid	LMFA01060029	371.244649	8.824366667	neg
4-Oxo-1-(3-pyridyl)-1-butanone	HMDB0062406	146.060394	4.160016667	pos
4-oxo-9Z,11Z,13E,15E-octadecatetraenoic acid	LMFA02000270	335.184997	9.541616667	neg
4-Oxo-norfloxacin	1600	665.215797	1.071433333	neg
4-Oxovalproic acid	2995	315.181923	8.022933333	neg
4-Phospho-N-pantothenoylcysteine	6014	420.120443	4.297333333	pos
4-Phosphopantothenoylcysteine	HMDB0001117	403.09327	6.08295	pos
4-Prenylresveratrol	53242	314.175604	5.157166667	pos
4-propionyl butyric acid	LMFA01060014	287.150712	6.190783333	neg
4-Trimethylammoniobutanal	6179	130.123074	0.548016667	pos
4-Trimethylammoniobutanoic acid	966	146.117896	3.951716667	pos
5-(1-Propynyl)-5'-vinyl-2,2'-bithiophene	HMDB0038430	459.037489	5.7905	neg
5-(2'-Carboxyethyl)-4,6-Dihydroxypicolinate	58545	245.076519	7.495216667	pos
5-(2-Hydroxyethyl)-4-methylthiazole	HMDB0032985	166.029808	2.895883333	pos
5-(2-methoxyethyl)isolongifol-5-ene	53490	245.226751	0.4423	pos
5-(2-Methylpropyl)tetrahydro-2-oxo-3-furancarboxylic	87332	187.096886	0.966566667	pos
5-(4-Acetoxy-1-butynyl)-2,2'-bithiophene	HMDB0034454	551.048847	4.927216667	neg
5-(4-Chloro-3-hydroxy-1-butynyl)-2,2'-bithiophene	HMDB0033269	248.960775	0.6532	neg
5-(but-3-en-1-yn-1-yl)-5'-methyl-2,2'-bithiophene	LMFA12000352	459.037652	6.106483333	neg

5(S),6(R)-7-trihydroxymethyl Heptanoate	44893	383.19036	14.80295	neg
5,5-Dimethyl-2(5H)-furanone	265073	223.097733	7.4129	neg
5,6,7,8-Tetrahydro-2,4-dimethylquinoline	HMDB0029708	200.083582	0.566216667	pos
5,6,7,8-Tetrahydroquinoxaline	HMDB0033154	152.118531	15.47191667	pos
5,6-Dihydrouridine	HMDB0000497	291.083343	7.833116667	neg
5,6-dihydroxy stearic acid	35463	334.295653	8.880233333	pos
5,6-Dimethoxysterigmatocystin	HMDB0030592	423.045928	0.658466667	pos
5,6-Indolequinone-2-carboxylic acid	63501	381.038299	8.170533333	neg
5,7-Dihydroxy-3',4'-dimethoxy-5'-prenylflavanone	52935	365.140106	9.857533333	neg
5,7-nonadienal	LMFA06000048	156.138615	2.276166667	pos
5,8,11-Dodecatriynoic acid	74122	233.082158	7.4129	neg
5,8-tetradecadienoic acid	73931	247.167135	15.12948333	pos
5-[2H-Pyrrol-4-(3H)-ylidenemethyl]-2-furanmethanol	94586	178.086608	0.566216667	pos
5a-Androstan-3b-ol	57827	259.242661	0.505683333	pos
5-Acetamidopentanoate	63464	160.097138	0.7678	pos
5-Acetamidovalerate	HMDB0012175	142.086614	1.090316667	pos
5-aceto valeric acid	LMFA01060015	189.076486	4.379483333	neg
5-Acetylamino-6-amino-3-methyluracil	HMDB0004400	181.07249	15.92241667	pos
5a-Dihydrotestosterone sulfate	57975	369.174903	7.854216667	neg
5alpha-Androstan-3alpha,17beta-diol disulfate	HMDB0094682	475.14114	7.884433333	pos
5alpha-androstane-3alpha-ol-17-one sulfate	LMST05020001	369.174942	8.212733333	neg
5alpha-Dihydrodeoxycorticosterone	HMDB0060407	377.231874	10.594	neg
5alpha-Ethoxy-6beta-hydroxy-5,6-dihydrophysalin B	HMDB0029626	573.233805	11.55266667	pos
5-amino-1-(5-phospho-D-ribosyl)imidazole-4-carbox	HMDB0006273	677.085156	10.17338333	neg
5-Amino-6-(5'-phosphoribosylamino)uracil	3497	335.039349	5.453416667	neg
5a-Tetrahydrocortisol	LMST02030200	367.24649	10.9175	pos
5beta-cholestane	LMST01010085	395.364042	10.57348333	pos
5beta-Cholestane-3alpha,7alpha,12alpha,24,25,26-	LMST04030030	491.33602	4.942016667	pos
5beta-Cholestane-3alpha,7alpha,12alpha,24,26,27-	84786	491.33406	11.33671667	pos
5beta-Pregnane-3alpha,17alpha,20alpha-triol	70255	359.256071	5.404333333	pos
5-butyl-8-methyl-1,2,3,5-tetrahydroindolizine	263960	209.20168	6.7366	pos
5'-Carboxy meloxicam	HMDB0060779	380.001411	4.211566667	neg
5-Chloro-3-methylcatechol	66006	141.010345	4.219483333	pos
5-chloropentanoic acid	LMFA01090154	174.992518	9.878016667	pos
5-Deoxykievitol	47964	401.123545	11.56056667	neg
5E,8E,11E-hexadecatrienoic acid	LMFA01030135	273.182939	15.92241667	pos
5-Ethyl-2-(1-pyrrolidinyl)-2-cyclopenten-1-one	HMDB0039676	162.128085	4.8634	pos
5-FLUOROINDOLE-2-CARBOXYLIC ACID	44300	224.036987	4.948316667	neg
5H-Cyclopentapyrazine	94581	235.09783	8.845466667	neg
5-Heptyltetrahydro-2-oxo-3-furancarboxylic acid	HMDB0030994	211.133267	8.601116667	pos
5-Hexyltetrahydro-2-oxo-3-furancarboxylic acid	87328	215.128267	1.028233333	pos
5-hydroperoxy-7-[3,5-epidioxy-2-(2-octenyl)-cyclope	74460	335.184814	9.098566667	neg
5-hydroxy-2-oxo-4-ureido-2,5-dihydro-1H-imidazole-	HMDB0059663	240.996012	8.232333333	pos
5-Hydroxyconiferaldehyde	64178	212.092124	5.424933333	pos
5-Hydroxyconiferyl alcohol	HMDB0060398	197.081216	0.02505	pos
5-Hydroxyectoine	63420	203.067097	0.823166667	neg
5-Hydroxyemedastine	HMDB0060777	301.201241	8.27325	pos
5-Hydroxyindoleacetic acid	2975	209.092539	2.751633333	pos
5-Hydroxykynurenamine	58203	359.171991	7.6855	neg
5-hydroxymethyl-2-furanoate	86075	140.011003	0.6902	neg
5-Hydroxymethyluracil	HMDB0000469	283.069037	2.44705	neg
5'-Hydroxysulfapyridine glucuronide	2557	424.082284	0.98685	pos
5-Methoxytryptophol	58062	236.093068	10.17338333	neg
5-Methyl-2-thiophenecarboxaldehyde	HMDB0032431	171.01158	4.232083333	neg
5-Methylcytosine	HMDB0002894	126.066572	0.08705	pos

5-methyl-octadecanoic acid	LMFA01020216	316.321729	10.12496667	pos
5-methyl-tetradecanedioic acid	LMFA01170020	255.195916	8.007216667	pos
5-Methylthiopentanaldoxime	64513	146.064378	0.710516667	neg
5-Nonyltetrahydro-2-oxo-3-furancarboxylic acid	87337	301.166343	8.360383333	neg
5-O-Feruloylnigrumin	93388	901.291082	0.6532	neg
5-O-Methylembelin	68286	353.19754	9.794333333	neg
5-Oxododecanoic acid	45865	259.155682	6.359216667	neg
5-oxo-pentanoic acid	35847	231.08761	4.779683333	neg
5-Ribosylparomamine	71998	478.198467	9.632383333	pos
5S,6S-epoxy-15R-hydroxy-ETE	HMDB0062236	209.138706	4.297333333	pos
5S-HETE di-endoperoxide	74966	425.215485	12.2583	pos
5-Tetrazolyl-glycine	69671	285.080577	11.0283	neg
5-trans-PGA2	36164	352.248829	0.905683333	pos
6-(2,4-dihydroxyphenyl)-2-{3-[3-(2,4-dihydroxypheny	HMDB0126387	519.165373	14.8218	neg
6-(2-Chloroallylthio)purine	70866	225.001567	1.1528	neg
6-(3,4-Dihydroxyphenyl)-6a,12b-dihydro-3,10,11,12-	47226	469.076656	7.7066	neg
6-(Allylthio)purine	70821	210.079915	0.866166667	pos
6,6'-(1,2-phenylene)bis(1,3,5-triazine-2,4-diamine)	96304	295.116951	8.613483333	neg
6,7-Dihydro-4-(hydroxymethyl)-2-(p-hydroxypheneth	89304	265.14847	11.56056667	neg
6,7-dihydroxy stearic acid	35464	299.258808	11.99565	pos
6,7-Epoxy-9Z-octadecene	98276	284.295484	16.00321667	pos
6,8-Dihydroxypurine	58027	135.030762	0.8856	pos
6,9,12-hexadecatrienoic acid	34811	233.190526	10.695	pos
6,9-dioxo-decanoic acid	74718	245.103413	5.57985	neg
6,9-hexadecadienoic acid	34936	253.216743	9.733716667	pos
6-[2,3-dihydroxy-5-(3,5,6,7-tetrahydroxy-4-oxo-4H-cl	HMDB0125241	491.048414	6.085383333	neg
6-[5-({5-[(3-carboxy-2,5,6-trihydroxyphenoxy)carbon	HMDB0128336	667.076241	4.277283333	pos
6-{[3,7-dihydroxy-2-(1-hydroxy-3-methoxy-4-oxocycl	HMDB0128859	587.126663	2.363616667	neg
6-{6-carboxy-2-[3,4-dihydroxy-5-(3,4,5-trihydroxyber	HMDB0128335	667.077584	4.179616667	pos
6alpha-Fluoro-11beta,17-dihydroxypregn-4-ene-3,2(	70662	365.211979	9.5911	pos
6alpha-Glucuronosylhyodeoxycholate	LMST05010016	569.333755	14.282	pos
6-Chlorobenzene-1,2,4-triol	66367	318.978465	0.7282	neg
6-Demethylsterigmatocystin	41023	291.02923	0.614716667	neg
6-Deoxy-5-ketofructose 1-phosphate	63568	243.027068	0.712516667	pos
6-deoxyerythronolide B	4986	387.272563	10.89728333	pos
6-Dimethylaminopurine	5460	325.164664	9.77325	neg
6E,11Z-hexadecadien-1-ol	36510	256.264065	10.28636667	pos
6-Epi-7-isocucurbic acid glucoside	86489	392.22848	9.391516667	pos
6-hydroxy-4-tridecanolide	97399	273.171517	6.759416667	neg
6-hydroxy-4-Undecanolide	LMFA07040031	245.139813	6.991116667	neg
6-Hydroxy-5-methoxyindole glucuronide	61676	320.075949	0.710516667	neg
6-Hydroxyfluvastatin	HMDB0014037	408.161809	13.15671667	neg
6-Hydroxymelatonin	7013	293.115116	4.484816667	neg
6-Hydroxypentadecanedioic acid	HMDB0031885	311.183794	8.539533333	pos
6-hydroxysphingosine	53903	338.267187	10.04253333	pos
6-keto PGE1	LMFA03010012	351.215028	9.652616667	pos
6-Ketoestriol	LMST02010053	303.159524	8.4576	pos
6-keto-PGF1alpha	LMFA03010001	371.243085	9.4326	pos
6-Keto-prostaglandin F1a	HMDB0002886	371.243187	9.61175	pos
6-Lactoyltetrahydropterin	HMDB0002065	284.099355	0.6902	neg
6-Methoxy-3-(2-thiazolyl)-1H-indole	HMDB0038632	459.094457	8.022933333	neg
6-Methoxyluteolin 7-glucuronide	LMPK12111211	473.073599	6.991116667	neg
6-Methoxymusizin 8-O-[b-D-glucopyranosyl-(1->6)-b	HMDB0034614	893.303591	0.6357	neg
6-methyl-dodecanedioic acid	LMFA01170013	245.175176	13.68475	pos
6-methylnicotinamide	85256	299.150564	6.864716667	neg

6-methyltetrahydropterin	6572	182.104136	1.108216667	pos
6-Methylthiohexanaldoxime	64516	321.169009	8.782183333	neg
6-Nitrobenzo[a]pyrene	73166	315.113368	9.71345	pos
6-pentadecyl Salicylic Acid	45469	349.274988	4.821233333	pos
6-Phosphonoglucono-D-lactone	HMDB0001127	241.011325	0.866166667	pos
6-Thioguanosine monophosphate	HMDB0060415	424.032319	4.927216667	neg
6-Thioxanthine	724	190.999069	0.7678	pos
6Z,11Z-hexadecadien-1-ol	36511	256.264083	15.90231667	pos
6Z,9Z-hexadecadienoic acid	LMFA01030273	253.216871	0.02505	pos
7(14)-Isodaucen-10-one	53501	238.21699	10.12496667	pos
7,11-Bisdeacetylvaltrate 7-(3-methylpentanoate) 11-	89454	539.25086	6.14865	neg
7,11-hexadecadien-1-ol	36480	256.264114	9.3094	pos
7,7'-Dihydroxy-6,8'-bicoumarin	89369	340.081332	4.8634	pos
7,8-Diaminononanoate	3324	189.16014	0.658466667	pos
7,8-Didehydroastaxanthin	LMPR01070051	577.369172	10.06316667	pos
7,8-Dihydro-3-methylpyrrolo[1,2-a]pyrimidin-2(6H)-o	93190	299.150661	7.580016667	neg
7,8-Dihydropteroic acid	HMDB0001412	315.120819	10.14556667	pos
7,8-dihydroxy stearic acid	35465	299.258811	9.980383333	pos
7a-Hydroxydehydroepiandrosterone	HMDB0004611	349.200535	10.29983333	neg
7a-Hydroxytestosterone	2790	349.200578	9.646666667	neg
7alpha-Hydroxy-3-oxo-4-cholestenoate	HMDB0012458	431.316446	11.1996	pos
7beta,12alpha-Dihydroxy-3-oxo-5beta-cholestan-26-	LMST04030049	487.281554	10.22716667	pos
7-chloro-2-(1-hydroxy-3-methoxy-4-oxocyclohexa-2,	HMDB0135841	419.055707	4.211566667	neg
7-Chloro-3,3',4',5,6,8-hexamethoxyflavone	88599	417.073854	5.28485	neg
7-Chloroemodin	41041	305.022606	4.160016667	pos
7-Drimene-11,12,14-triol	91869	299.187119	8.782183333	neg
7-EPITAXOL	84960	834.314059	14.2669	neg
7-Ethoxy-4-methyl-2H-1-benzopyran-2-one	HMDB0035095	222.112925	1.991466667	pos
7-Ethyltridecan-6-one	98246	227.237349	0.002433333	pos
7-F2-dihomo-IsoP	LMFA03110170	383.279895	10.02156667	pos
7-hydroxy-10-heptadecen-8-ynoic acid	LMFA01050260	327.218426	10.29983333	neg
7-Hydroxy-5,4'-dimethoxy-8-methylisoflavone 7-O-rh	47846	915.310437	14.23088333	neg
7-Hydroxy-5-methoxyflavan	LMPK12020258	295.074268	2.35865	pos
7-HYDROXYETHYLTHEOPHYLLINE	44525	247.079467	5.280883333	pos
7-Hydroxymethyl-12-methylbenz[a]anthracene sulfa	HMDB0060420	335.074171	0.712516667	pos
7-hydroxyolanzapine	HMDB0060958	327.127422	12.64993333	neg
7-Hydroxyticlopidine	HMDB0013921	297.08294	0.505683333	pos
7-keto-n-caprylic acid	LMFA01060021	203.092305	4.463766667	neg
7-Methoxy-2-methylisoflavone	47525	311.093298	8.044016667	neg
7-methoxy-dodec-4-enoic acid	96773	246.206912	6.124133333	pos
7-Methyl-3-methylene-1,6,7-octanetriol	LMFA05000635	187.133622	4.6322	neg
7-methyl-4-oxo-octanoic acid	74789	173.11765	1.844116667	pos
7-Methylhypoxanthine	HMDB0003162	151.061965	1.090316667	pos
7-O-Demethylterazosin	2722	745.343704	14.54056667	neg
7-oxo-11E-Tetradecenoic acid	45868	285.171453	8.402566667	neg
7-oxo-11Z-Tetradecenoic acid	45869	285.171446	7.643316667	neg
7-oxo-8-amino-nonanoic acid	LMFA01060168	232.119239	4.211566667	neg
7-Prenyloxy-3',4'-dimethoxyisoflavone	47571	365.140053	11.23385	neg
7S,8S-DiHODE	35692	313.237965	10.22716667	pos
7Z,10Z-Hexadecadienoic acid	HMDB0000477	297.207847	10.594	neg
7Z-nonadecenoic acid	74034	341.270296	14.65188333	neg
7Z-tetradecenoic acid	HMDB0062243	209.190437	11.97545	pos
8(R)-Hydroperoxylinoleic acid	HMDB0004706	313.23802	10.63491667	pos
8,8a-Deoxyoleandolide	LMPK04000030	395.241792	9.91855	pos
8,9-DiHETrE	HMDB0002311	339.25371	10.95795	pos

8-Acetylegelolide	92587	331.115862	9.391516667	pos
8-Acetyl-T2 tetrol	91311	321.133062	7.580016667	neg
8-Azaadenosine	68949	267.085707	7.49725	neg
8E,10E-dodecadienoic acid	34903	214.180583	13.68475	pos
8E-Heptadecenedioic acid	74925	299.222172	9.795466667	pos
8E-heptadecenoic acid	LMFA01030287	269.247901	0.02505	pos
8-HETE	HMDB0004679	321.242366	10.67518333	pos
8-hydroxy-13Z-octadecene-9,11-diynoic acid	74651	335.184979	9.857533333	neg
8-hydroxy-17-octadecene-10,12-diynoic acid	74653	335.185004	9.267316667	neg
8-Hydroxy-4(6)-lactarene-5,14-diol	HMDB0035780	299.18712	8.276	neg
8-Hydroxy-5,6-octadienoic acid	HMDB0031101	201.076642	4.674333333	neg
8-iso-15-keto-PGE2	36383	373.196846	10.30701667	pos
8-Methylnonenoate	HMDB0012183	215.128852	7.244233333	neg
8-Methylthiooctanaldoxime	64518	377.231679	10.9664	neg
8-Nonen-2-one	LMFA12000145	185.117935	8.402566667	neg
8-O-Methyloblongine	90043	309.174757	13.25091667	neg
8-oxo capric acid	LMFA01060032	204.159876	1.802133333	pos
8-oxo-9,11-octadecadiynoic acid	LMFA02000271	335.185114	8.9931	neg
8-Oxodiacetoxyscirpenol	92034	381.152616	8.820583333	pos
8-Pentanoylneosolaniol	93229	505.182129	10.22716667	pos
8-Propanoylneosolaniol	93233	477.150687	9.473266667	pos
8R-HpODE	35388	330.264438	9.652616667	pos
8-Undecynoic acid	74272	165.127715	0.548016667	pos
9-(3,4-Dimethoxyphenyl)-2-methoxy-1H-phenalen-1-	90142	385.08259	9.774883333	pos
9(S)-HpODE	36019	330.264292	10.61448333	pos
9(S)-HpOTrE	36025	333.204048	10.695	pos
9,10,13-TriHOME	HMDB0004710	331.248562	10.22716667	pos
9,10-DHOME	HMDB0004704	359.244861	9.7099	neg
9,10-DiHOME	LMFA02000229	295.228423	11.13138333	neg
9,10-dihydroxy-hexadecanoic acid	LMFA01050191	306.264594	10.4506	pos
9,10-Epoxyoctadecenoic acid	HMDB0004701	314.269474	10.6551	pos
9,12,13,TriHODE	LMFA02000220	373.224021	7.643316667	neg
9,12,13-TriHOME	HMDB0004708	329.234147	7.769866667	neg
9,12,15-Octadecatrien-1-ol	LMFA05000216	247.242438	0.484533333	pos
9,12-dioxo-dodecanoic acid	74730	211.133285	4.055983333	pos
9,12-Octadecadien-1-ol	LMFA05000217	284.295363	14.31686667	pos
9,12-octadecadienal	LMFA06000101	282.279586	12.05626667	pos
9,13-dihydroxy-10-ethoxy-11-octadecenoic acid	LMFA01050121	381.263376	10.53256667	pos
9,13-dihydroxy-12-ethoxy-10-octadecenoic acid	LMFA01050122	341.267131	11.97545	pos
9-Aminocamptothecin	71369	402.085547	5.796033333	pos
9-Bromo-16alpha-methyl-pregn-4-ene-3,11,20-trione	70420	419.122982	9.62565	neg
9-dodecen-1-ol	LMFA05000024	185.190375	0.002433333	pos
9-Fluoro-11beta-hydroxypregna-4,16-diene-3,20-dio	70572	691.381995	13.56321667	neg
9-Fluoro-16alpha-hydroxyandrost-4-ene-3,11,17-trio	70598	379.156148	9.794333333	neg
9-Hexadecenoylcholine	HMDB0013208	363.311039	12.52048333	pos
9-hydroperoxy-12,13-epoxy-10-octadecenoic acid	LMFA02000105	346.259518	8.498583333	pos
9-Hydroxy-10-O-D-glucuronoside-12Z-octadecenoat	HMDB0060121	473.272824	2.585966667	pos
9-hydroxy-hexadecan-1,16-dioic acid	74927	285.206343	9.227683333	pos
9-Keto heptadecylic acid	LMFA01060059	267.232332	10.22716667	pos
9-keto lauric acid	LMFA01060040	259.155684	7.32855	neg
9-octadecenal	LMFA06000099	284.295281	13.96595	pos
9-oxo-2,4,5,7-decatetraenoic acid	LMFA01060086	196.097321	1.0697	pos
9-oxo-2E-decenoic acid	74720	229.108304	7.455083333	neg
9-oxo-2Z-decenoic acid	74721	367.211055	11.31588333	neg
9-Oxo-nonanoic acid	HMDB0094711	217.108159	6.84365	neg

9-OxoODE	HMDB0004669	295.227248	10.63491667	pos
9R,10S-dihydroxy-stearic acid	LMFA02000007	315.25464	10.73845	neg
9R,10S-EpOME	LMFA02000283	341.233961	10.67736667	neg
9R,10S-Epoxy-3Z,6Z-eicosadiene	LMFA12000309	310.310955	14.12413333	pos
9R-hydroxy-2E-decenoic acid	35604	371.244694	9.920666667	neg
9S,10S,11R-trihydroxy-12Z,15Z-octadecadienoic ac	LMFA02000021	329.232859	9.85735	pos
9S,11R,15S-trihydroxy-2,3-dinor-13E-prostaenoic a	36203	327.21843	10.04675	neg
9S-hydroxy-2E-decenoic acid	35605	231.124014	5.874783333	neg
9-tetradecenal	75357	193.195543	0.505683333	pos
9-tridecynoic acid	LMFA01030602	228.1962	13.68475	pos
9-undecynoic acid	35271	183.138378	0.548016667	pos
9Z,12E-Tetradecadienal	46474	191.179775	0.002433333	pos
Abscisic alcohol	64130	251.164555	10.30701667	pos
Acaciabiuronic acid	HMDB0029938	379.084419	3.3304	pos
Acamprosate	HMDB0014797	162.022367	0.925983333	neg
Acetaldehyde butyl phenethyl acetal	HMDB0037820	245.151531	15.92241667	pos
Acetaldehyde hexyl isoamyl acetal	HMDB0032154	199.206105	1.9283	pos
Acetylcarnitine	956	204.123502	1.090316667	pos
Acetylcholine	57	146.117891	0.8261	pos
Acetylintermedine	71310	386.180541	9.225133333	neg
Acetylisoniazid	HMDB0041821	197.103824	15.98305	pos
ACETYL-L-CYSTEINE	784	164.037614	0.905683333	pos
Acetylpterosin C	HMDB0030764	321.134822	7.370716667	neg
Acetylsalvipisone	87994	707.362145	13.76268333	neg
Acetyl-T2 Toxin	HMDB0033164	547.19362	7.003433333	pos
Acifluorfen	HMDB0037112	343.994496	1.108216667	pos
Aconitine	7050	646.323137	5.692966667	pos
Acoric acid	HMDB0038165	269.175423	7.945816667	pos
Acorusdiol	HMDB0030919	235.169758	11.23941667	pos
Acridinium NHS ester	64846	463.129706	7.518333333	neg
Actinonin	43966	384.251332	4.863983333	neg
Adefovir	68958	318.061573	2.134433333	neg
Adenine	85	269.101428	7.81205	neg
Adenophostin A	69662	652.044613	4.219483333	pos
Adenosine monophosphate	HMDB0000045	348.070882	1.090316667	pos
Adenosine phosphosulfate	HMDB0001003	426.012897	0.6532	neg
Adenosine tetraphosphate	6191	625.924748	10.32751667	pos
Adipate semialdehyde	HMDB0012882	259.119266	5.516666667	neg
Adipic acid	LMFA01170048	191.055836	0.843633333	neg
ADMA	6891	203.150728	0.846116667	pos
Adouetine X	HMDB0034216	481.318056	10.80075	neg
Adrenic Acid	LMFA01030178	371.233838	5.878466667	pos
AF-2	73228	229.025144	5.495583333	neg
Agomelatine	HMDB0015636	226.123101	9.491233333	pos
Agrimol C	68479	713.280719	11.37706667	neg
Ajmalicine	64327	703.347385	0.6357	neg
AL-321	71623	336.104826	8.086216667	neg
Alanyl-Asparagine	HMDB0028682	186.087884	15.98305	pos
Alanyl-Histidine	85605	209.103925	15.98305	pos
Alanyl-Proline	85610	209.088891	0.584866667	pos
Alendronic acid	818	229.998445	0.74595	neg
Alginic acid	HMDB0029940	413.004497	10.7592	neg
Allantoic acid	343	157.036018	0.802716667	neg
Allixin	HMDB0040705	207.102541	8.5924	neg
Allocholic acid	HMDB0000505	453.286927	9.351683333	neg

Allodesmosine	HMDB0040704	636.350767	11.96663333	neg
Allopurinol	HMDB0014581	137.046148	1.090316667	pos
all-trans-13,14-dihydroretinol	LMPR01090047	271.243338	0.4423	pos
Alosetron	HMDB0015104	277.144152	0.548016667	pos
alpha,alpha-Dimethylanisalacetone	87272	249.113671	10.1101	neg
alpha-[3-(Nitrosoamino)propyl]-3-pyridinemethanol	73243	389.193082	10.90441667	neg
alpha-Butyl-omega-hydroxypoly(oxyethylene) poly(o	HMDB0032181	249.206691	9.1661	pos
alpha-Carboxy-delta-nonalactone	HMDB0030990	245.103413	5.916916667	neg
alpha-CEHC	44825	301.141586	0.641016667	pos
Alpha-cyclodextrin	85154	953.30348	0.6532	neg
Alpha-D-Glucose	HMDB0003345	225.061592	0.6902	neg
alpha-Eucaine	69845	665.381333	13.74465	neg
alpha-Fluoro-beta-ureidopropionic acid	HMDB0060435	299.081948	7.244233333	neg
alpha-hydroxy myristic acid	35391	245.211598	1.886166667	pos
alpha-Ketoisovaleric acid	HMDB0000019	231.087625	4.9694	neg
Alpha-Lactose	HMDB0000186	341.10958	2.238566667	neg
alpha-Linolenic acid	LMFA01030152	279.232161	11.11933333	pos
Alpha-N-Phenylacetyl-L-glutamine	58397	265.118779	4.779033333	pos
alpha-Thiophenecarboxylic acid	2193	254.979717	0.802716667	neg
Alpha-Tocotrienol	HMDB0006327	423.327878	14.74585	neg
alpha-Zearalenol	70304	338.196594	10.14556667	pos
Alpinumisoflavone dimethyl ether	47847	403.093221	15.47191667	pos
Alprazolam	929	307.074102	0.710516667	neg
ALPRENOLOL	44522	232.170112	7.24955	pos
Alternariol	HMDB0030831	303.050795	2.134433333	neg
AM1220	85062	381.195899	14.8218	neg
Amaranth	70314	626.914147	9.350416667	pos
Amastatin	65611	497.259457	15.98305	pos
Ambrettolic acid	LMFA01050106	315.218273	8.88765	neg
Americanin B	92272	475.141162	8.27325	pos
Ametryn	72478	228.128864	0.786766667	pos
Aminoadipic acid	HMDB0000510	160.060858	0.843633333	neg
Aminoparathion	6284	242.040701	5.916916667	neg
AMIPRILOSE	44328	306.191728	7.270016667	pos
Amisulpride	HMDB0015633	387.206662	4.568983333	pos
Ammodendrine	68134	415.307471	10.29983333	neg
Ammothamnine	68548	303.147621	4.379516667	pos
Amobam	72255	491.01485	11.0901	neg
Amoxapine	982	314.104602	0.622116667	pos
AMPROLIUM	43586	243.159627	6.103533333	pos
Amygdalin	HMDB0035030	438.139073	7.622216667	neg
Amylopectin	3696	873.274492	1.09225	neg
Anandamide (20:2, n-6)	36738	374.303653	12.60105	pos
Anandamide (20:4, n-6)	LMFA08040001	348.288046	12.39953333	pos
Anapheline	64435	225.196544	6.185533333	pos
Anatabine	7061	178.134239	0.002433333	pos
Androsterone sulfate	3559	369.17492	6.717266667	neg
Angelic acid	LMFA01020029	199.097327	7.49725	neg
Angustifoline	68549	257.162863	15.86195	pos
Anhydroicaritin 3-(6'''-acetylgalactosyl)(1->3)-rhamn	LMPK12112024	881.318863	0.658466667	pos
Annonisin	87647	593.443306	14.87983333	pos
Annuionone B	88204	267.124442	9.667733333	neg
Annuionone C	88627	225.148988	9.1045	pos
Anofinic acid	HMDB0033303	222.112923	1.0697	pos
antazoline	3996	266.165745	6.267383333	pos

Antibiotic GR 95647X	HMDB0033034	445.258312	4.779033333	pos
Apicidin	96431	668.365451	15.82238333	neg
Aplidiasphingosine	LMSP01080016	392.314297	12.3592	pos
Aprepitant	HMDB0014811	515.132467	13.15671667	neg
Arachidonic Acid (d8)	3805	351.25364	10.95795	pos
Arachidonoyl dopamine	43429	462.299013	10.99823333	pos
Arachidonoyl-EA(d8)	46565	378.32209	11.71338333	pos
Arachidyl carnitine	58411	909.788051	13.30693333	neg
Arborinine	68458	308.088596	9.652616667	pos
Arbutin	HMDB0029943	253.072246	5.348033333	neg
Argininosuccinic acid	389	291.130498	0.749333333	pos
Arginyl-Glutamate	85623	347.145892	9.77325	neg
Arginyl-Glutamic acid	HMDB0028708	304.161986	0.806416667	pos
Arginyl-Glutamine	85622	341.132606	5.837283333	pos
Arginyl-Tryptophan	85635	361.197118	9.795466667	pos
Armillaripin	HMDB0030404	437.194439	10.22716667	pos
Artelinic acid	1116	293.137938	7.580016667	neg
Artobiloxanthone	LMPK12110939	433.1287	11.68271667	neg
Artocarpetin B	LMPK12110908	421.103855	4.779033333	pos
Artoindonesianin B	LMPK12111537	513.176223	11.80461667	neg
Artomunoxanthentrione	49950	443.114654	10.23661667	neg
Artonin R	HMDB0041125	543.16345	15.35605	neg
AS-604850	45220	329.988322	4.211566667	neg
AS-605240	45073	513.042448	0.67025	neg
Ascorbic acid-2-sulfate	HMDB0060649	254.982116	0.905383333	neg
Ascorbigen	HMDB0029839	323.123313	10.87708333	pos
Ascorbyl palmitate	HMDB0039883	413.255526	9.878583333	neg
Asparaginyl-Gamma-glutamate	85660	519.217575	10.73845	neg
Asparaginyl-Glutamate	85645	241.06978	5.6641	neg
Asparaginyl-Proline	85654	274.105154	0.843633333	neg
Asparaginyl-Threonine	HMDB0028741	214.083383	13.90668333	neg
Aspartame	HMDB0001894	312.155755	3.993383333	pos
Aspartyl-Glutamine	85666	260.089389	0.802716667	neg
Aspartylglycosamine	5476	336.140608	0.712516667	pos
Aspartyl-Isoleucine	HMDB0028756	247.129209	3.972533333	pos
Aspartyl-Leucine	HMDB0028757	229.118755	1.090316667	pos
Aspartyl-Tryptophan	85678	320.122645	5.569266667	pos
Aspirin	HMDB0001879	225.040672	5.095883333	neg
Asp-Phe methyl ester	6377	295.129522	4.484716667	pos
Atenolol	HMDB0001924	249.159605	4.5058	pos
Austalide L	86764	446.254684	10.22716667	pos
Avenanthramide 1s	HMDB0029285	361.139921	5.589866667	pos
Avocadene 2-acetate	HMDB0031044	346.295816	9.491233333	pos
Avocadene 4-acetate	HMDB0031045	373.260375	9.267316667	neg
AVOCADYNE	43517	302.269467	9.186616667	pos
Avocadyne 4-acetate	87373	327.253664	11.23941667	pos
Azaserine	72922	345.080664	2.134433333	neg
Azelaic acid	HMDB0000784	233.103313	5.937966667	neg
Azoxy-2-procarbazine	2107	253.164942	4.59005	pos
Bacampicillin	HMDB0015540	446.140369	10.21556667	neg
Bakankoside	68006	380.132039	1.090316667	pos
Bambuterol	85510	390.199177	1.090316667	pos
BAY-11-7082	45239	188.017288	2.2176	neg
bayogenin 3-O-cellobioside	LMPR0106150009	857.450356	13.09973333	neg
BBT(OH)2	LMFA12000350	213.02274	6.359216667	neg

b-D-Glucopyranosiduronic acid	2024	324.107182	10.0258	neg
Behenic acid	HMDB0000944	358.368679	10.97813333	pos
Behenoyl-EA	3727	366.373822	12.46005	pos
Benomyl	68666	289.130067	5.390233333	neg
benoxinate	3995	309.217989	6.287966667	pos
BENZALKONIUM	44355	304.300552	10.30701667	pos
Benzamide	44739	139.087008	15.92241667	pos
Benzamideoxime	96504	271.119368	7.138766667	neg
Benzo[ghi]perylene	69963	299.0826	2.7309	pos
Benzoyl phosphate	66355	247.001463	7.138766667	neg
Benzoylagmatine	65739	257.136414	6.041716667	pos
Benzyl ethyl ether	HMDB0031312	154.122937	11.73355	pos
Benzyl methyl disulfide	HMDB0040590	339.038	5.7905	neg
Benzyl salicylate	86523	267.041466	0.712516667	pos
Benzyl trans-2-methyl-2-butenoate	HMDB0032177	379.192256	11.47903333	neg
Benzylsuccinate	67953	207.066169	6.675116667	neg
Bergaptol	HMDB0013679	185.023801	0.786766667	pos
beta-Caryophyllene	LMPR0103120001	205.195483	1.865133333	pos
Beta-Cityl-L-glutamic acid	HMDB0013220	322.077533	1.249116667	pos
Beta-Cortol	58325	369.264295	10.53256667	pos
beta-Dolabrin	LMPR0102110001	163.075652	1.991466667	pos
beta-Elemenone	53387	219.174792	0.357716667	pos
Betanin	HMDB0029408	595.143091	2.760483333	neg
beta-Zearalanol	70307	340.212443	0.566216667	pos
Bialaphos	63603	368.122851	7.518333333	neg
Bikhaconitine	67099	718.346198	12.47065	neg
Bilirubin glucuronide	HMDB0010332	741.275884	11.56056667	neg
Biliverdin	HMDB0001008	583.255599	9.3094	pos
Biochanin A	HMDB0002338	283.061949	6.801533333	neg
Biocytin	3509	353.164517	13.7265	neg
Biotin-XX hydrazide	64812	523.246946	12.31893333	pos
Biotinyl-5'-AMP	HMDB0004220	554.124455	1.09225	neg
Biphenyl	66444	172.112384	2.23505	pos
Bis(4-nitrophenyl)phosphate	66043	339.004033	5.13805	neg
Bis(chloromethyl) ether	72897	136.953538	1.048966667	pos
Bisacurone epoxide	HMDB0038503	286.201728	9.61175	pos
Bisdiphosphoinositol tetrakisphosphate	HMDB0006230	802.788677	15.16526667	pos
Bismuth Subsalicylate	HMDB0015408	360.991672	10.38415	neg
Bisphenol A	69602	273.114076	10.53113333	neg
Blighinone	HMDB0030643	311.020475	15.48823333	neg
Blumealactone C	HMDB0035357	369.156175	5.432366667	neg
BMS 204352	69722	358.025079	0.6532	neg
Bn-NCC-2	HMDB0038230	791.322162	0.6357	neg
Botrydial	LMPR0103640001	291.160881	10.88373333	neg
Brassicanal A	68795	172.02225	0.6902	neg
Brassicanal B	93257	214.033305	4.842916667	neg
Brassilexin	HMDB0039638	347.044312	8.14945	neg
Brassitin	HMDB0040977	203.06416	5.857883333	pos
Broussoflavan A	LMPK12020243	444.239097	2.895883333	pos
Buddledin A	67850	277.1804	9.632383333	pos
Bumetanide	1393	409.10896	10.80075	neg
Butabarbital	HMDB0014382	195.113534	4.118683333	pos
Butralin	72384	313.187558	4.179616667	pos
Butyl (S)-3-hydroxybutyrate glucoside	87870	303.145628	9.119666667	neg
Butyl 3-O-beta-D-glucopyranosyl-butanoate	98328	303.14561	5.853733333	neg

Butyl 4'-O-butanoyl-6-O-hexadecanoyl-neohesperid(	LMSL05000005	691.462206	10.24565	pos
Butyl 4'-O-hexadecanoyl-neohesperidoside	LMSL05000004	603.410782	10.34803333	pos
Butyl butyryllactate	LMFA07010792	261.134992	5.516666667	neg
Butyl cinnamate	92525	222.149339	4.484716667	pos
Butyl dodecanoate	88155	274.27459	14.95071667	pos
Butyl ethyl malonate	91378	169.086457	6.1697	neg
Butyl undecylenate	LMFA07010797	258.243339	9.247933333	pos
Butyrylcholine	85236	213.112607	1.028233333	pos
Byrsonic acid	LMFA01050424	457.35096	13.13813333	pos
C.I. Food Red 1	HMDB0033383	481.039662	11.2953	neg
C16 Sphinganine	41556	274.27453	8.742433333	pos
C17 Sphinganine	41558	288.290203	9.45315	pos
C-6 NBD Ceramide	63013	576.373056	10.32751667	pos
C75	44901	255.159566	9.0643	pos
Caffeic Acid	3316	225.040578	5.411316667	neg
Caffeine	1455	195.088122	4.673916667	pos
Caftaric acid	HMDB0013680	293.031083	4.737533333	neg
Cajanol	HMDB0033924	355.058336	0.658466667	pos
Calcimycin	68980	546.258021	10.695	pos
Calcium glycerophosphate	69510	208.953357	1.09225	neg
Calomelanol G	52837	471.085209	9.491233333	pos
Calpeptin	68942	385.210261	11.65356667	pos
Camalexin	93292	199.03337	0.6902	neg
Camptothecin	3491	371.101941	1.886166667	pos
Canavaninosuccinate	62849	293.109661	8.921266667	pos
Capric acid	LMFA01010010	155.143348	1.760266667	pos
Caproic acid	LMFA01010006	134.117903	0.8856	pos
Caproylcholine	85237	241.143833	6.429366667	pos
Caprylic acid	112	189.112882	5.727266667	neg
Capryloylcholine	HMDB0013225	269.175376	8.109516667	pos
Capsianoside IV	HMDB0030736	667.32756	4.199916667	pos
Capsiate	90202	324.217503	0.92605	pos
Capsicum annuum Fluorescent chlorophyll catabolit	90808	667.251932	11.55266667	pos
Carbocysteine sulfoxide	1527	233.982613	0.7678	pos
CARBOFURAN	44573	266.104109	10.34203333	neg
Carboprost Tromethamine	85382	507.366004	11.33671667	pos
Carboxyltolmetin-3-glucuronide	2877	508.110607	7.833116667	neg
Carglumic acid	HMDB0015673	191.066636	0.749333333	pos
Carnosine	HMDB0000033	227.114304	0.658466667	pos
Casbene	LMPR0104290001	273.258563	0.42115	pos
CAY10404	63043	385.082696	15.92241667	pos
CAY10563	45276	294.006217	5.13805	neg
CAY10564	45252	237.970963	0.69355	pos
CDP-4-dehydro-6-deoxy-D-glucose	63237	570.049173	10.12496667	pos
CDP-ethanolamine	3538	445.05502	0.67025	neg
CDP-ribitol	63138	576.03842	10.36856667	pos
CE(15:0)	HMDB0060057	609.558718	15.16576667	neg
Ceanothine D	86202	467.30235	5.7342	pos
cefadroxil	3948	364.097673	4.179616667	pos
Cefuroxime	1628	423.062718	5.348033333	neg
Cefuroxime sodium	66823	447.059089	5.363416667	pos
Cellulose, microcrystalline	HMDB0032197	353.145903	8.048083333	pos
Cepharadione A	89877	286.052076	10.1101	neg
Cerasinone	LMPK12140530	311.093257	8.697816667	neg
Ceriporic acid A	45935	327.253726	10.97813333	pos

Ceriporic acid C	45936	375.251347	12.0361	pos
Cerivastatin	66775	458.234554	13.08056667	neg
Cerotic acid(d3)	45760	438.38008	12.46005	pos
Cerulenin	44856	241.15494	2.11315	pos
Cervonyl carnitine	LMFA07070055	494.325386	10.38908333	pos
CGP71422	HMDB0013864	527.287653	9.473266667	pos
CGS 7181	69725	405.106819	9.16185	neg
Charine	HMDB0039877	297.079393	10.79605	pos
Chenodeoxycholic Acid	LMST04010032	437.291531	10.55208333	neg
Chenodeoxycholic acid 3-glucuronide	57940	569.334349	14.5433	pos
Chenodeoxycholic acid glycine conjugate	HMDB0000637	432.311309	9.473266667	pos
Chenodeoxyglycocholate	43193	448.307937	9.478266667	neg
Chidamide	64835	435.146909	7.601116667	neg
Chloramphenicol alcohol	638	315.084123	2.342766667	neg
Chlormephos	72466	214.953311	14.89833333	neg
Chloropentafluoroethane	HMDB0031333	134.942446	0.74595	neg
Chlorphenesin carbamate	66767	226.02779	5.7905	neg
Cholestane-3,7,12,25-tetrol-3-glucuronide	HMDB0010355	595.385255	10.55303333	pos
Cholesteryl 11-hydroperoxy-eicosatetraenoate	LMST01020029	705.582971	15.49035	pos
Cholic acid	LMST04010001	453.286846	10.53113333	neg
Cholic acid glucuronide	57962	567.31858	10.22716667	pos
Chorismate	HMDB0012199	207.029786	4.758616667	neg
Chrycolide	90481	255.008395	9.878016667	pos
Chymosin preparation, escherichia coli k-12	HMDB0032199	708.108222	4.097783333	pos
Cibaric acid	LMFA02000289	323.18483	9.225133333	neg
Cicerin 7-(6-malonylglucoside)	95084	559.109586	9.62565	neg
Cimetidine	HMDB0014644	297.113853	9.62565	neg
cimicifoetiside B	LMST01100007	685.39709	11.98685	neg
Cinalukast	85405	457.180648	10.92508333	neg
Cincassiol B	91863	423.199807	10.12496667	pos
Cinnamic acid	HMDB0000567	166.086641	2.895883333	pos
Cinnamyl acetate	69422	221.08202	8.276	neg
Cinnamyl alcohol	65764	135.080789	0.002433333	pos
cis- and trans-5-Ethyl-4-methyl-2-(2-butyl)-thiazoline	HMDB0032201	369.240439	7.012216667	neg
cis- and trans-Ethyl 2,4-dimethyl-1,3-dioxolane-2-ac	HMDB0032200	233.103273	4.82185	neg
cis-10-Hydroxylinalyl oxide 7-glucoside	89103	349.184035	15.98305	pos
cis-2,3-Dihydro-2,3-dihydroxybiphenyl	66445	233.082195	7.833116667	neg
cis-3-Hexenyl cis-3-hexenoate	LMFA07010805	197.15409	10.73525	pos
cis-3-Hexenyl crotonate	LMFA07010806	191.104552	15.92241667	pos
cis-3-Hexenyl pentanoate	LMFA07010807	202.180595	6.124133333	pos
cis-3-Hexenyl pyruvate	93001	215.092471	5.095883333	neg
cis-3-Hexenyl tiglate	HMDB0038279	200.164928	12.82123333	pos
cis-4-(1'-Hydroxynaphth-2'-yl)-2-oxobut-3-enoate	69030	241.051039	6.569816667	neg
cis-4-octenedioic acid	45920	343.140596	5.600916667	neg
cis-5-Tetradecenoylcarnitine	6437	370.296032	9.85735	pos
cis-6,7-Epoxy-2-methylheptadecane	LMFA12000279	286.310904	10.57348333	pos
cis-9-palmitoleic acid	LMFA01030056	255.23235	0.002433333	pos
cis-Ethyl 4-heptenoate	HMDB0032265	311.223624	9.81545	neg
cis-gondoic acid	3554	355.286101	12.35023333	neg
Cis-stilbene oxide	HMDB0059631	197.096502	10.67518333	pos
Cis-zeatin	HMDB0012204	237.146697	15.84153333	pos
Citpressine II	HMDB0029324	333.145039	6.20565	pos
Citric acid	124	191.019349	1.112466667	neg
Citronellyl acetate	LMFA07010812	243.160524	9.225133333	neg
Citronellyl formate	41141	229.1447	8.5924	neg

Citronellyl hexanoate	HMDB0038958	237.221668	1.0697	pos
Citronellyl pentanoate	LMFA07010819	258.243419	11.27946667	pos
Citronellyl propionate	92191	195.17484	11.53246667	pos
Citronellyl trans-2-methyl-2-butenoate	HMDB0032209	256.227639	13.63223333	pos
Citroside A	HMDB0030370	367.176917	10.17338333	neg
Citrulline	16	174.087844	0.76405	neg
Clarycet	91207	245.139816	7.32855	neg
Clausarinol	HMDB0041407	432.239004	9.473266667	pos
clavulone I	LMFA03120001	485.192972	9.549883333	pos
Clitidine 5'-phosphate	66968	331.034265	8.5924	neg
Cloethocarb	72711	240.043486	4.211566667	neg
Clomifene	HMDB0015020	388.184236	9.85735	pos
CLOPIDOL	43547	235.988419	15.90565	neg
CLOVANEDIOL DIACETATE	44495	323.221938	10.30701667	pos
CMP-3-deoxy-D-manno-octulosonate	63651	588.110859	2.718633333	neg
CMPF	45041	239.092822	7.896366667	neg
Codeine	498	298.145625	11.74368333	neg
Coenzyme Q4	LMPR02010003	472.341636	14.08883333	pos
Coenzyme Q6	LMPR02010002	613.424753	16.00321667	pos
Colnelenic acid	46569	293.211417	10.30701667	pos
Colupox a	94154	461.2555	11.06958333	neg
Compound II(R/S)	66414	779.337099	14.522	neg
Coniferyl alcohol	HMDB0012915	163.075648	0.548016667	pos
Convolvulinolic acid	35424	259.227413	0.002433333	pos
Corchoionol C 9-glucoside	86479	367.177062	9.457166667	neg
Coriandrone B	92590	293.139	0.566216667	pos
Coronatine	71344	342.168228	4.097783333	pos
corticosterone	LMST02030186	347.220088	11.0386	pos
Cortisol	HMDB0000063	407.208452	6.738333333	neg
Cortisol 21-acetate	41869	449.219097	11.0077	neg
Cortol	HMDB0003180	386.290617	10.53256667	pos
Cortolone	63340	367.247026	10.59398333	pos
Cortolone-3-glucuronide	61643	541.26669	5.980083333	neg
Coumarin-4-carboxylic acid	HMDB0032944	379.0444	7.601116667	neg
CPA(18:0/0:0)	HMDB0007004	438.29893	11.27946667	pos
CPX	44518	322.222993	8.068566667	pos
Creatine	7	132.077146	0.8261	pos
Crucigasterin 277	LMSP01080036	278.248513	11.85445	pos
Cucujolide IV	LMFA07040036	239.129182	10.0258	neg
Cucurbitacin E	LMST01010107	579.293791	1.5754	pos
Cuminaldehyde	44723	193.086646	13.7265	neg
Curcumenone	53443	233.154868	11.23385	neg
Curcumin I	HMDB0039611	377.138111	9.62565	neg
Cuscohygrine	3362	225.196628	11.4121	pos
CV 2961	70961	356.077934	7.290466667	pos
Cyanidin 3-(diferuloylsophoroside) 5-glucoside	92775	273.197855	10.21556667	neg
Cyanidin 3-O-(2''-O-galloyl-6''-O-alpha-rhamnopyran	46874	769.155891	9.774883333	pos
Cyanolide A	65368	831.470681	13.41736667	neg
Cyclamate	73081	401.078583	15.48823333	neg
Cyclandelate	HMDB0015586	315.134579	6.75715	pos
Cycloartomunoxanthone	HMDB0029999	447.144694	13.13778333	neg
Cyclobrassinin	HMDB0033352	217.024841	0.002433333	pos
Cyclobrassinone	89779	213.012868	1.112466667	neg
CYCLOCREATINE	44140	166.058925	15.92241667	pos
Cyclomillinol	48251	651.293948	4.505883333	neg

Cycloneosamadione	263652	286.217143	1.949316667	pos
Cyclopassifloic acid A	91129	537.3809	11.21971667	pos
Cyclopassifloside III	93097	843.470734	13.96011667	neg
Cypendazole	72678	657.290802	10.73845	neg
Cyprodinil	HMDB0034853	226.134059	2.012516667	pos
Cystamine	55	197.042787	4.590083333	neg
Cysteineglutathione disulfide	HMDB0000656	427.096198	0.866166667	pos
Cysteinyl-Aspartate	HMDB0028771	217.029708	0.74595	neg
Cysteinyl-Glutamine	85687	288.040247	2.092966667	pos
Cysteinyl-Serine	85698	415.094556	7.096583333	neg
Cytidine	3376	244.093328	1.090316667	pos
Cytidine monophosphate	HMDB0000095	304.034697	0.905383333	neg
Cytidine triphosphate	HMDB0000082	463.966303	4.232083333	neg
Cytochalasin Opho	HMDB0035366	450.26392	10.3631	neg
D-1-Piperideine-2-carboxylic acid	5992	145.097521	1.090316667	pos
D-4'-Phosphopantothenate	3427	298.070302	2.155183333	neg
D8'-Merulinic acid A	HMDB0041454	391.284897	0.002433333	pos
D-altro-D-manno-Heptose	HMDB0029952	233.063662	0.749333333	pos
Dapsone N-sulfamate	1932	327.01051	0.710516667	neg
D-Arginine	6924	157.108749	15.92241667	pos
Daucic acid	HMDB0031665	407.045119	4.211566667	neg
Davallioside A	47260	574.130577	2.668766667	pos
Davanone	HMDB0038178	259.167203	15.49035	pos
D-Cathinone	63533	194.081988	10.17338333	neg
d-Dethiobiotin	3351	259.130466	4.295183333	neg
Debrisoquine	58453	193.145213	15.49035	pos
Decarbamoylsaxitoxin	HMDB0038319	237.111075	7.244233333	neg
Deferasirox	HMDB0015547	418.105471	4.590083333	neg
Deferoxamine	1946	561.362909	10.36856667	pos
Dehydroanonaine	86908	246.091177	0.905683333	pos
Dehydroepiandrosterone sulfate	HMDB0001032	367.159201	7.601116667	neg
Dehydrogriseofulvin	72602	331.036394	4.927216667	neg
Dehydrooreadone	HMDB0036046	217.122725	10.26573333	pos
Dehydrorotenone	44627	431.088308	6.185533333	pos
Dehydrovomifoliol	91833	223.133258	0.548016667	pos
Dehydroxyzyleuton	HMDB0013970	203.064166	6.226183333	pos
Dehydrozingerone	71922	237.077148	6.275	neg
Demethylbatatasin IV	53260	229.087269	9.9837	neg
Demethylcalabaxanthone	87123	417.108924	7.270016667	pos
Demethylcitalopram	1782	619.290101	11.49943333	neg
Demethylphosphinothricin	63600	167.034209	0.357716667	pos
Deoxycholic acid	HMDB0000626	437.291763	10.6976	neg
Deoxycholic acid 3-glucuronide	HMDB0002596	569.334387	14.8265	pos
Dermorphin	73515	801.35629	13.54503333	neg
Derricin	LMPK12120009	367.155787	10.1945	neg
D-Erythritol 4-phosphate	65986	247.022817	5.36915	neg
D-erythro-D-galacto-octitol	86611	241.09205	0.67025	neg
D-Erythrose 4-phosphate	355	199.001018	0.946716667	neg
Desethyl-N-acetylprocainamide	2103	497.289945	11.19298333	neg
Desmethylergometrine	2564	329.196245	8.41665	pos
Desoximetasone	HMDB0014687	421.203039	12.78818333	neg
Dexmethylphenidate	HMDB0015647	251.175796	3.972533333	pos
Dextrin	HMDB0006857	549.168405	0.884666667	neg
Dezocine	66779	263.212424	5.857883333	pos
D-Fructose	68675	225.061578	5.032616667	neg

D-Fructose 1-phosphate	378	261.037603	0.712516667	pos
DG(12:0/8:0/0:0)	HMDB0093019	423.308082	15.90231667	pos
DG(19:0/22:0/0:0)[iso2]	LMGL02010190	677.641605	14.36916667	pos
DG(20:2(11Z,14Z)/22:3(10Z,13Z,16Z)/0:0)[iso2]	4582	737.550701	13.9131	pos
DG(21:0/22:0/0:0)[iso2]	LMGL02010251	705.672819	15.11163333	pos
DG(22:0/22:0/0:0)	LMGL02010258	719.688549	15.11163333	pos
DG(8:0/10:0/0:0)	HMDB0092901	390.322139	9.529233333	pos
DG(i-21:0/22:0/0:0)	HMDB0094387	705.672761	14.3866	pos
DG(i-24:0/0:0/a-21:0)	HMDB0094571	733.703753	15.12948333	pos
D-Galactosamine	63181	160.060865	1.1528	neg
DGCC(20:5/20:5)	LMGL00000133	802.564082	15.3814	pos
D-Glutamine	HMDB0003423	129.066277	0.806416667	pos
D-Glyceraldehyde 3-phosphate	3294	152.995256	0.566216667	pos
D-Glyceric acid	65498	230.981732	1.029866667	neg
D-glycero-L-galacto-Octulose	HMDB0029954	263.074311	0.749333333	pos
DHAP(10:0)	HMDB0011675	342.168303	15.92241667	pos
Di-2-propenyl tetrasulfide	HMDB0033202	190.948239	7.854216667	neg
Di-2-thienyl disulfide	HMDB0037173	210.916364	15.96818333	neg
Diammonium hydrogen phosphate ((NH4)2HPO4)	90213	263.051845	5.263766667	neg
Diatretin 2	HMDB0031004	163.050476	1.886166667	pos
Dibenzofuran	66731	213.055733	5.980083333	neg
Dibenzo-p-dioxin	66733	229.050881	6.780483333	neg
Dibutyl disulfide	HMDB0029569	179.093266	15.94263333	pos
Dibutyl phthalate	HMDB0033244	301.14168	15.92241667	pos
Dichloroethanol	2923	136.953518	1.907233333	pos
didesmethyl tocotrienol	53842	367.264889	13.49036667	neg
Didodecyl thiobispropanoate	HMDB0040172	553.366597	8.820583333	pos
Diethyl Oxalpropionate	44744	247.082766	5.57985	neg
Diethyl tartrate	89393	229.068685	8.820583333	pos
Diethylphosphate	HMDB0012209	199.037599	0.864066667	neg
Diethylpropion (metabolite XI Glucuronide)	2025	416.157231	7.622216667	neg
Diethylpropion(metabolite V-glucuronide)	2021	378.15409	9.62565	neg
Difloxacin	68932	422.129264	5.424933333	pos
Dihydro-2,4,6-tris(2-methylpropyl)-4h-1,3,5-dithiazin	88271	290.196592	6.75715	pos
Dihydro-2,4-dimethyl-6-(2-methylpropyl)-4H-1,3,5-di	HMDB0040334	188.093513	15.98305	pos
Dihydro-6-isopropyl-2,4-dimethyl-4H-1,3,5-dithiazine	HMDB0040332	192.088464	15.98305	pos
Dihydroamorphigenin	48026	457.149985	12.49058333	neg
Dihydrocapsiate	HMDB0034781	291.195709	10.95795	pos
Dihydroceramide	58540	330.300974	10.08378333	pos
Dihydrocortisol	HMDB0003259	363.216168	10.67736667	neg
Dihydroformononetin	47952	269.082673	7.32855	neg
Dihydrofukinolide	90117	410.25462	2.052666667	pos
Dihydrojasmone	HMDB0031565	211.133943	9.794333333	neg
DIHYDROJASMONIC ACID, METHYL ESTER	43916	271.155896	8.402566667	neg
Dihydromyoporone	HMDB0035729	253.180239	8.519066667	pos
Dihydropanaxacol	HMDB0032675	325.202859	9.43605	neg
Dihydrosanguinarine	64356	314.083658	11.0283	neg
Dihydroxyacetone phosphate	HMDB0001473	152.995247	3.8686	pos
Dihydroxyacetone Phosphate Acyl Ester	HMDB0011750	394.976671	8.550233333	neg
Diloxanide	2265	231.99395	5.116983333	neg
Dimethicone	HMDB0033532	145.086257	1.886166667	pos
Dimethyl 3-methoxy-4-oxo-5-(8,11,14-pentadecatrie	HMDB0032099	421.257066	10.49155	pos
Dimethyl trisulfide	66929	143.997152	0.786766667	pos
Dimethylenetriurea	66392	407.188343	15.35605	neg
dIMP	HMDB0006555	350.085114	0.712516667	pos

Dinorcapsaicin	HMDB0036325	258.150441	11.90591667	neg
Diosbulbin A	90447	421.14906	7.622216667	neg
Dioscoretine	93255	264.157708	6.654433333	pos
Diphenoxylate	2327	435.241018	4.139566667	pos
Diphosphoinositol tetrakisphosphate	HMDB0060271	640.840762	9.899633333	neg
DIPROTIN A	44309	681.452774	14.35758333	neg
Disopyramide	HMDB0014425	357.264204	10.02156667	pos
Disperse Yellow 3	73115	287.149132	6.185533333	pos
Distemonanthin	LMPK12113389	357.023857	4.927216667	neg
Distichonic acid A	93404	277.10373	0.905683333	pos
Dityrosine	58352	359.125817	5.0748	neg
DL-Acetylcarnitine	LMFA07070060	202.10832	5.263766667	neg
D-Lactic acid	63094	135.030158	2.155183333	neg
DL-Dopa	64960	178.050532	6.359216667	neg
D-Linalool 3-glucoside	HMDB0030418	299.186154	7.638666667	pos
DMABA-d6 NHS ester	96492	307.09688	8.4576	pos
D-Mannitol	142	183.086671	0.749333333	pos
Docosanamide	5565	340.358096	12.1168	pos
dodecanamide	LMFA08010001	182.190785	5.424933333	pos
Dodecanedioic acid	HMDB0000623	229.144692	8.086216667	neg
Dodecanoic acid	HMDB0000638	183.174798	11.89476667	pos
Dodecyl acetate	46261	229.216629	0.02505	pos
Dodecyl butyrate	HMDB0032249	274.274658	11.43183333	pos
Dodecyl propionate	LMFA07010843	260.259011	7.9663	pos
Dolineone	LMPK12060008	317.046511	14.91738333	neg
DOPA sulfate	HMDB0002028	276.018204	2.03025	neg
D-Pantetheine 4'-phosphate	398	357.088831	5.7905	neg
D-Phenyllactic acid	HMDB0000563	167.070672	10.38908333	pos
D-Ribitol 5-phosphate	63137	277.033134	0.76405	neg
D-Ribose	HMDB0000283	195.050755	0.76405	neg
D-Ribose 5-phosphate	HMDB0001548	229.012018	0.74595	neg
Droperidol	HMDB0014593	757.328593	0.6532	neg
Droxidopa	85558	231.098065	1.090316667	pos
D-Tagatose 6-phosphate	HMDB0006873	283.01942	0.712516667	pos
dTDP	HMDB0001274	385.021107	1.127133333	pos
dTDP-D-glucuronate	66343	559.037626	5.36915	neg
dTDP-L-olivose	63721	550.119513	2.173566667	pos
Dyspropterin	HMDB0001195	220.082042	0.7678	pos
E-64	44981	358.20787	4.7158	pos
Ecabet	HMDB0015613	379.157924	13.15671667	neg
Ectoine	43384	125.0714	15.80033333	pos
Edulan I	HMDB0034959	175.148508	8.662066667	pos
Edulane	LMPK12070102	349.145268	11.23385	neg
Eicosanedioic acid	35987	387.276176	10.44721667	neg
Elaeocarpidine	68411	312.172804	10.0047	neg
Elaeokanine C	68413	256.15598	5.537733333	neg
Elaidic carnitine	58415	426.358625	10.83661667	pos
Elemicin	68334	209.117622	0.548016667	pos
Eletriptan N-oxide	HMDB0060898	443.164813	10.57303333	neg
ELV-N34	LMFA01050554	567.38204	9.733716667	pos
Enigmol	LMSP01080058	284.295306	10.81631667	pos
Enprofylline	HMDB0014962	177.077645	15.98305	pos
ent-16b,19-Kauranediol 19-acetate	91816	393.263546	10.594	neg
Ephedrine	2541	166.123043	4.160016667	pos
Epimelibiose	3476	365.106133	0.806416667	pos

Eplerenone	HMDB0014838	453.168448	10.22716667	pos
Eprosartan	66654	407.142576	9.632383333	pos
Eremopetasinorol	86412	226.180611	13.50863333	pos
Eremopetasitenin A2	HMDB0030900	377.143046	12.5306	neg
Ergocornine	67476	606.293464	10.73845	neg
Ergothioneine	53	230.096275	0.905683333	pos
Erinapyrone B	95824	125.060163	1.823116667	pos
Eriojaposide B	HMDB0038029	561.257197	10.6567	neg
erythro-6,8-Pentacosanediol	HMDB0041075	767.788426	14.7269	neg
Erythronolide B	63708	385.259266	10.61448333	pos
Erythroskyrin	HMDB0030464	473.265765	10.22716667	pos
Eseramine	67484	301.164724	6.859766667	pos
Estradiol-17-phenylpropionate	70228	449.234372	11.90591667	neg
Estramustine	HMDB0015327	877.331407	13.45395	neg
Estrone	HMDB0000145	315.160725	11.43826667	neg
Eszopiclone	HMDB0014546	371.10198	1.048966667	pos
Ethanesulfonic acid, 2-(1-(difluoro-((trifluoroethenyl)[illegible]	88302	582.888091	9.350416667	pos
Ethephon	72208	162.007712	1.007533333	pos
Ethiolate	72837	321.169027	9.246216667	neg
Ethoate-methyl	72742	226.012535	11.77376667	pos
Ethyl (2E,6Z)-dodecadienoate	HMDB0031015	247.167194	14.91533333	pos
Ethyl (S)-3-hydroxybutyrate glucoside	87869	312.165767	5.363416667	pos
Ethyl 10-undecenoate	HMDB0034286	230.2119	7.782066667	pos
Ethyl 3-(N-butylacetamido)propionate	72595	216.159925	7.515733333	pos
Ethyl 3,3-diiodoacrylate	LMFA01090139	350.838949	0.593983333	neg
Ethyl 3-cyclohexylpropionate	87658	167.143425	1.802133333	pos
Ethyl 3-O-beta-D-glucopyranosyl-butanoate	LMFA13010042	275.114376	5.01155	neg
Ethyl 4-methylphenoxyacetate	89776	195.102071	10.38908333	pos
Ethyl 4Z-octenoate	LMFA07010872	215.12887	8.086216667	neg
Ethyl 9-hexadecenoate	HMDB0059871	283.263598	2.875483333	pos
Ethyl glucuronide	HMDB0010325	205.070085	15.98305	pos
Ethyl menthane carboxamide	92642	256.192292	10.55208333	neg
Ethyl methyl-p-tolylglycidate	HMDB0037492	238.144223	3.847833333	pos
Ethyl octynecarboxylate	91363	165.127788	9.7543	pos
Ethyl pentanoate	94821	259.19213	8.001833333	neg
Ethyl tiglate	HMDB0038962	255.160669	9.267316667	neg
Ethyl undecanoate	HMDB0029552	197.190448	1.739483333	pos
Ethyladipic acid	45928	175.096903	1.048966667	pos
Ethylendiamine dihydroiodide	72193	358.875102	4.211566667	neg
Ethylene brassylate	94961	271.190675	8.3757	pos
etizolam	96166	387.068227	8.001833333	neg
Etoxazole	72301	342.168167	0.641016667	pos
eucalyptol	4020	137.132822	4.055983333	pos
Euphorbia factor Ti2	67420	583.292305	11.11075	neg
Exiguaflavanone M	52604	460.233862	1.2291	pos
FAD	HMDB0001248	784.150914	4.295183333	neg
FAPy-adenine	7080	136.062189	15.98305	pos
Farnesal	44756	203.179893	0.336533333	pos
Fauronyl acetate	HMDB0036422	325.202765	9.014183333	neg
FENTHION	44242	259.001706	5.874783333	neg
Fenugreekine	93995	662.103696	1.09225	neg
Fenuron	68925	327.182079	7.434	neg
Fertaric acid	HMDB0029199	325.057228	5.053716667	neg
ferulic acid	4156	177.055024	0.548016667	pos
Filipin III	63055	672.428715	10.28636667	pos

Flonicamid	72269	247.079455	5.589866667	pos
Floxuridine	HMDB0014467	491.122154	9.62565	neg
Fludrocortisone	2715	398.232959	12.76165	pos
Flumazenil	HMDB0015336	286.097406	1.090316667	pos
Fluoren-9-one	66498	181.065081	0.002433333	pos
Fluorene	66720	331.150228	9.62565	neg
Fluorescein	2755	377.067413	0.67025	neg
Fluorodifen	68807	327.024267	1.112466667	neg
Flupenthixol	HMDB0015013	475.141476	16.00321667	pos
Flutamide	HMDB0014642	299.06257	1.9283	pos
Fluvoxamine acid	2951	336.153379	9.774883333	pos
Fluxofenim	72510	308.03154	5.916916667	neg
Foetidin	HMDB0033271	365.211352	4.014216667	pos
Forasartan	85498	397.227334	14.54056667	neg
Formononetin	HMDB0005808	313.072687	9.267316667	neg
Formylfusarochromanone	93578	365.136062	2.718633333	neg
Fosfomycin	HMDB0014966	156.04247	0.658466667	pos
Fosfomycin calcium	69748	193.989556	0.566216667	pos
Frovatriptan	HMDB0015133	266.125289	1.090316667	pos
Frutinone A	67347	247.038158	0.622116667	pos
Fumonisin AK1	89237	621.394818	10.02156667	pos
Furfural diethyl acetal	69937	339.181161	10.17338333	neg
Furfuryl isovalerate	HMDB0039874	183.101963	0.548016667	pos
Furfuryl octanoate	92553	269.139679	6.338166667	neg
Furfuryl pentanoate	LMFA07010892	227.09259	5.7694	neg
Gabapentin	2989	154.122913	12.54061667	pos
Galactaric acid	HMDB0000639	191.019369	0.74595	neg
Galactinol dihydrate	62462	423.134768	9.62565	neg
Gallopamil	2999	507.28367	4.65325	pos
GalNAcalpha(1-3)[Fucalpha(1-2)]Galbeta(1-4)GlcN/	3656	846.309941	0.6532	neg
gamma glutamyl ornithine	6571	284.122116	6.021116667	pos
gamma-Butyrobetaine Ethyl Ester Chloride	73569	213.112729	5.342783333	pos
Gamma-Caprolactone	HMDB0003843	227.129069	7.769866667	neg
gamma-Carotene	LMPR01070260	575.402039	11.33671667	pos
gamma-Glutamylarginine	HMDB0029143	302.147526	0.823166667	neg
gamma-Glutamylisoleucine	HMDB0011170	261.145045	4.297333333	pos
Gamma-Glutamyltyrosine	62464	311.124416	3.247416667	pos
gamma-L-Glutamyl-gamma-L-glutamyl-L-methionine	93329	425.169116	7.4543	pos
gamma-L-Glutamyl-L-pipecolic acid	93275	276.156038	0.806416667	pos
Ganoderic acid Md	90631	631.387455	13.56321667	neg
Gefitinib	45475	485.113703	12.72153333	pos
Gemcitabine	565	525.137298	12.49058333	neg
Gentamicin C2	3054	446.297122	5.301516667	pos
Gentisate aldehyde	HMDB0004062	139.039338	1.028233333	pos
Gentisic acid	618	153.018648	4.526916667	neg
Geraniol	41060	137.132796	0.39995	pos
Geranyl 2-methylbutyrate	HMDB0032291	261.182948	15.98305	pos
Geranyl acetoacetate	LMFA07010896	239.164541	7.863983333	pos
Geranyl diphosphate	400	359.067791	7.601116667	neg
Geranyl tiglate	HMDB0032292	259.167245	15.98305	pos
Geranyl valerate	HMDB0040281	261.181881	10.14556667	pos
Geranylgeraniol	53578	308.295434	13.3276	pos
Geranyl-hydroxybenzoate	64005	319.155726	8.44475	neg
Gestrinone	3087	353.176981	10.8423	neg
Gibberellin A12	41226	377.195629	9.81545	neg

gibberellin A19	LMPR0104170039	723.33517	0.6532	neg
Gibberellin A20	41213	377.159108	9.857533333	neg
Gibberellin A3	HMDB0003559	345.132722	10.88373333	neg
Gibberellin A36	41231	723.339597	13.96011667	neg
Gibberellin A51	LMPR0104170022	377.158986	9.583833333	neg
Gibberellin Ga115	HMDB0032820	361.167343	14.43023333	neg
Gingerenone B	HMDB0035405	387.180892	10.22716667	pos
Ginsenoyne C	HMDB0038994	275.165995	10.7181	neg
Ginsenoyne K	HMDB0040375	315.134624	7.5362	pos
Glabrin D	HMDB0033613	784.351343	14.43023333	neg
Glucobrassicanapin	HMDB0038417	405.099282	6.08295	pos
Glucoerucin	HMDB0038403	420.047195	4.127283333	neg
Gluconapoleiferin	66957	384.042921	4.927216667	neg
Glucoraphanin	HMDB0038404	418.030616	1.235383333	neg
Glucosamine 6-phosphate	HMDB0001254	258.038821	0.710516667	neg
Glucosyl (2E,6E,10x)-10,11-dihydroxy-2,6-farnesadi	HMDB0037823	433.242561	9.85735	pos
Glucosylgalactosyl hydroxylysine	HMDB0000585	467.190055	13.49036667	neg
Glu-P-2	72966	202.108931	0.002433333	pos
Glutamylarginine	HMDB0028813	304.161973	0.69355	pos
Glutamyl-Asparagine	85728	305.085523	11.66235	neg
Glutamylglutamic acid	HMDB0028818	277.103796	1.090316667	pos
Glutaric acid	HMDB0000661	131.035521	15.54703333	neg
Glutathione	44	306.077147	1.071433333	neg
Gluten exorphin C	HMDB0059796	650.39599	6.30855	pos
Glycerol 2-phosphate	44760	171.005826	0.74595	neg
Glycerol 3-phosphate	HMDB0000126	217.012066	1.09225	neg
Glycerol tributanoate	HMDB0031094	303.18098	8.048083333	pos
Glycerol trihexanoate	87428	369.264297	10.30701667	pos
Glycerol tripropanoate	HMDB0032857	241.108494	6.422383333	neg
Glycerophosphocholine	370	258.11065	0.7301	pos
Glyceryl 5-hydroxydecanoate	HMDB0032297	301.141581	3.785533333	pos
Glyceryl lactooleate	LMFA07010900	411.308868	13.96595	pos
Glycidyl oleate	73127	361.271563	4.65325	pos
Glycine xylidide	1001	179.118297	4.297333333	pos
Glycineamideribotide	HMDB0002022	267.038864	5.7905	neg
Glycinexylidide	HMDB0060679	355.21285	9.75215	neg
Glycochenodeoxycholate-3-sulfate	HMDB0002497	528.264877	7.896366667	neg
Glycocholic Acid	LMST05030001	464.30267	8.065116667	neg
Glycogen	160	684.255597	0.905683333	pos
Glycoursodeoxycholic acid	57909	450.3201	12.58093333	pos
Glycyrin	LMPK12160017	421.106661	16.00321667	pos
Glyodin	72700	309.32708	11.17941667	pos
Glyphosine	72874	264.004404	0.69355	pos
Gomphrenin II	HMDB0039898	677.159108	9.667733333	neg
Gomphrenol	LMPK12112878	359.039425	4.927216667	neg
Gossypetin 8-glucuronide 3-sulfate	HMDB0037752	557.025695	10.14556667	pos
Grevilline B	HMDB0033240	321.041784	5.13805	neg
guaifenesin	4057	395.171767	10.17338333	neg
Guanine	315	152.056981	1.090316667	pos
Guanosine	87	284.09934	2.11315	pos
Guanosine 3',5'-bis(diphosphate)	HMDB0059638	601.952144	4.211566667	neg
Guanosine 3'-phosphate	63402	362.051443	1.112466667	neg
Halopemide	45490	415.133709	9.7099	neg
Harmalan	86538	367.19208	11.84515	neg
Harman	HMDB0035196	363.161094	10.94575	neg

Harmine	43924	423.182306	10.78	neg
Heliangin	HMDB0036692	361.164162	9.62565	neg
Heliannuol A	94199	251.164561	1.907233333	pos
Helinorbisabone	88199	273.110333	15.94263333	pos
Hellicoside	68344	701.192845	0.905383333	neg
Hepoxilin A3	LMFA03090005	381.228875	10.29983333	neg
Hepoxilin B3	HMDB0004690	381.228932	10.594	neg
Heptadecanal	HMDB0031039	272.295299	10.14556667	pos
Heptadecanedioic acid	LMFA01170028	301.238072	10.9175	pos
Heptadecanoyl carnitine	58363	414.358633	11.59303333	pos
Heptaethylene glycol	HMDB0061835	344.228487	4.673916667	pos
Heptanoylcholine	85240	255.159555	7.577283333	pos
Heptyl heptanoate	HMDB0034461	211.206012	0.002433333	pos
Heptylmalonic acid	HMDB0059719	201.113026	7.81205	neg
Herbacetin 8-acetate	LMPK12113165	345.061013	5.589866667	pos
Herculin	HMDB0030275	234.221987	7.24955	pos
Hericenone E	HMDB0039140	612.426116	2.895883333	pos
Heteroflavanone B	HMDB0040836	409.166584	11.11075	neg
Heteropyrithiamine	63859	218.140404	15.43553333	pos
Hexadecanedioic acid	5642	285.207771	10.57303333	neg
hexadecanedioic acid mono-L-carnitine ester	75447	430.317426	10.63491667	pos
Hexaethylene glycol	HMDB0061822	300.202203	4.463633333	pos
Hexahydro-6,7-dihydroxy-5-(hydroxymethyl)-3-(2-hy	92246	593.161779	11.39748333	neg
Hexamethylphosphoramide	72970	357.229165	9.457166667	neg
Hexanal dihexyl acetal	HMDB0032316	269.284731	0.39995	pos
Hexanoylcarnitine	HMDB0000705	258.171631	5.8116	neg
Hexyl decanoate	88363	274.274774	16.00321667	pos
Hexyl dodecanoate	HMDB0061946	329.270377	10.86301667	neg
hexyl octanoate	LMFA07010448	273.207873	8.718916667	neg
Hildgardtene	LMPK12020282	365.139973	10.63578333	neg
Hippuric acid	HMDB0000714	180.065993	2.092966667	pos
Histidine	65529	309.132825	7.2653	neg
Histidinol phosphate	363	239.089548	15.86195	pos
Histidinyl-Hydroxyproline	85792	307.079836	2.56525	pos
Homocarnosine	270	241.129873	0.7678	pos
Homocysteine thiolactone	6594	155.987509	3.972533333	pos
Homofukinolide	HMDB0034659	859.465437	13.65406667	neg
Homogentisic acid	331	169.049853	6.551633333	pos
Homomethionine	64484	208.06472	0.864066667	neg
Homovanillin	HMDB0005175	149.059983	0.526866667	pos
Hordatine A glucoside	86994	711.350281	14.37586667	neg
Hordatine B glucoside	HMDB0030460	741.356043	13.63593333	neg
Hordenine	7055	148.112373	5.713583333	pos
HYCANTHONE	43996	339.153073	10.20713333	pos
Hydantoin-5-propionic acid	6084	155.045383	4.219483333	pos
Hydralazine	3163	161.082476	0.002433333	pos
Hydralazine acetone hydrazone	HMDB0060606	218.140433	11.85445	pos
hydrocinnamic acid	4153	151.075659	8.109516667	pos
Hydroxyhexamide	HMDB0060610	309.127122	5.569266667	pos
Hydroxyhydroquinone	506	251.056586	5.853733333	neg
Hydroxymethylphosphonate	63590	222.978111	4.758616667	neg
Hydroxynicorandil	1522	272.051613	4.253166667	neg
Hydroxyphenylacetylglycine	HMDB0000735	208.061451	5.179716667	neg
Hydroxyprolyl-Alanine	85765	247.093943	1.050633333	neg
Hydroxystenozole	70527	349.224697	5.3837	pos

hydroxyzine	4069	355.158884	13.7265	neg
Hypogeic acid	45810	237.221687	0.69355	pos
Hypoglycin B	66881	288.154928	7.515733333	pos
Hypoxanthine	83	137.046125	2.133433333	pos
Ichangin 4-glucoside	93922	633.255751	9.125	pos
ICI 164384	70312	564.383053	10.57348333	pos
Icilin	64735	292.072829	0.7282	neg
Idebenone Metabolite (1,4-Benzenediol, 2-(10-hydrc	763	341.232896	10.30701667	pos
Idebenone Metabolite (Benzenedecanoic acid, 2-hy	767	435.168278	7.904866667	pos
Idebenone Metabolite (Benzenehexanoic acid, 2,5-c	772	321.131599	14.17651667	pos
IdoA2S-beta-1,4-GlcNAc(3S)-alpha-1,4-GlcA-beta-1	HMDB0062475	217.99893	1.167366667	pos
Ile Glu Leu Asn Glu	265031	655.267006	8.96225	pos
Imetit	71953	339.117955	5.832683333	neg
Imidacloprid	68851	294.015003	0.7678	pos
Imidazoleacetic acid riboside	HMDB0002331	276.119647	0.846116667	pos
Imidiocarb	96330	329.153292	9.62565	neg
Iminoaspartic acid	HMDB0001131	261.036184	5.4745	neg
Imiquimod	85419	285.135049	5.57985	neg
Immepip	71954	188.115887	2.194133333	pos
Indole-3-carboxilic acid-O-sulphate	HMDB0060002	263.993264	0.866166667	pos
Indole-3-ethanol	6932	144.081052	4.160016667	pos
Indoleacetaldehyde	6068	204.066496	5.495583333	neg
Indoxacarb	72372	566.034791	10.57348333	pos
Indoxyl	HMDB0004094	178.05053	6.232883333	neg
Indoxyl sulfate	HMDB0000682	212.002262	4.6322	neg
Inosine	84	269.088562	2.133433333	pos
Inosine 2',3'-cyclic phosphate	HMDB0011680	313.034847	4.9249	pos
Inosinic acid	HMDB0000175	393.044905	5.053716667	neg
Iodate	HMDB0001061	195.946145	0.712516667	pos
Ipomeamaronol	92889	289.141624	10.79605	pos
Ipomeatetrahydrofuran	HMDB0040904	301.202726	9.225133333	neg
Iridotrial	41149	165.091372	0.002433333	pos
Isazofos	72752	294.023898	5.13805	neg
Isoamyl 2-furonpropionate	92558	211.133331	0.966566667	pos
Isoangustone A	47751	843.33739	13.49036667	neg
Isobutyl 10-undecenoate	HMDB0032347	285.207894	9.18295	neg
Isobutyl 2-furanpropionate	92557	179.106962	0.002433333	pos
Isobutyl cinnamate	HMDB0037703	222.14936	4.884483333	pos
Isochamaejasmin	47514	525.117991	8.293766667	pos
Isocrotonic acid	LMFA01030194	171.065773	5.4745	neg
iso-Debromo-laurinterol	53401	234.185734	0.463416667	pos
Isoderricin A	LMPK12140014	367.155825	10.0047	neg
Isoelemicin	71476	209.117635	5.81665	pos
Isoeugenyl acetate	HMDB0032348	224.128531	2.012516667	pos
Isogemichalcone B	51869	971.325153	13.59966667	neg
Isogingerenone B	90696	404.207659	9.85735	pos
Isokanugin	LMPK12112733	401.088795	7.7066	neg
Isoleucylproline	HMDB0011174	251.137065	4.568983333	pos
Isolinderanolide	92862	337.274431	11.23941667	pos
Isomethiozin	72849	269.142131	5.65175	pos
Isomucronulator 7-O-glucoside	LMPK12080004	509.165098	9.457166667	neg
Isomugineic acid	89365	319.114475	8.086216667	neg
Isoniazid	HMDB0015086	138.066614	15.94263333	pos
Isoniazid pyruvate	71268	413.120363	10.88373333	neg
Isonicotinamide, 2-propyl-	2182	327.182018	9.14075	neg

Isonicotinylglycine	HMDB0041912	163.050502	1.718916667	pos
Isopalmitic acid	HMDB0031068	274.274634	12.94041667	pos
Isopentenyladenine	6612	405.22683	10.38415	neg
Isopentenyladenine-9-N-glucoside	HMDB0012240	725.375886	13.54503333	neg
Isophosphamide mustard	HMDB0060691	264.992271	8.044016667	neg
Isopropyl beta-D-glucoside	88639	240.144694	4.44255	pos
Isopropyl beta-D-ThiogalactoPyranoside	44762	221.085316	0.002433333	pos
Isopropyl citrate	88388	273.09498	15.43553333	pos
Isopropyl tetradecanoate	LMFA07010677	288.290219	9.023766667	pos
Isoproterenol	HMDB0015197	229.155145	1.090316667	pos
Isoprothiolane	68852	335.061752	2.134433333	neg
Isoricinoleic Acid	74507	343.249743	9.878583333	neg
Isouron	72239	421.258578	10.6567	neg
Isouvaretin	52270	417.109089	6.880283333	pos
Isovalerylalanine	5715	172.097445	4.569016667	neg
Isovalerylglutamic acid	HMDB0000726	232.117358	16.00321667	pos
Isoxaben	72310	663.339895	14.30311667	neg
Isoxathion	70175	358.052865	8.5924	neg
ISOXICAM	44108	336.065314	5.363416667	pos
Istamycin C1	71992	414.270411	0.641016667	pos
I-Urobilin	58222	571.292091	11.06958333	neg
Jaceidin 4'-glucuronide	LMPK12112931	517.099775	7.096583333	neg
Jadomycin B	63768	567.231979	9.878016667	pos
Jasmolone	86681	163.112033	0.002433333	pos
Jasmonic acid	HMDB0032797	228.159814	5.959266667	pos
Jesaconitine	67126	676.335196	4.160016667	pos
JP83	45144	439.200512	10.22716667	pos
Jubanine A	HMDB0030205	694.360556	14.28501667	neg
Juvenile hormone III	41205	267.196072	9.227683333	pos
JWH 018 N-pentanoic acid metabolite-d4	96671	393.210482	16.00321667	pos
JWH 200 4-hydroxyindole metabolite	96658	383.175161	3.9309	pos
JWH022	85139	384.16194	13.7265	neg
Kaempferide 3-rhamnoside	LMPK12112037	445.115127	7.53855	neg
Kaempferol	HMDB0005801	285.041214	5.432366667	neg
Kaempferol 3,7,4'-tri-O-sulfate	LMPK12112000	543.950736	4.6111	pos
Kamahine C	93546	251.128368	8.3757	pos
Kanzonol E	48470	371.163803	14.3866	pos
Ketamine	881	473.175672	9.81545	neg
Ketoleucine	121	259.119297	5.8116	neg
Kifunensine	45180	277.067443	5.7694	neg
Kiwiionoside	93346	407.227303	4.160016667	pos
Kynuramine	43923	327.182063	8.42365	neg
L-2-Amino-4-methylenepentanedioic acid	HMDB0029433	317.098594	12.92578333	neg
Labetalol	912	655.351169	12.47065	neg
Lactapiperanol C	89430	305.172832	9.959816667	pos
Lactosamine	HMDB0006591	359.164659	0.622116667	pos
Lactosylceramide (d18:1/12:0)	7123	788.548818	15.3633	pos
LAGOCHILIN	44390	401.255308	10.27876667	neg
L-alpha-Aspartyl-L-hydroxyproline	62009	291.083302	6.44345	neg
Lankamycin	71014	831.470684	13.65406667	neg
L-Arginine	13	175.119335	0.6755	pos
L-Asparagine	14	133.061161	0.69355	pos
L-Aspartic Acid	15	134.045151	0.712516667	pos
Latanoprost ethyl amide-d4	96571	460.276158	5.322166667	pos
Lauric acid	LMFA01010012	183.174726	0.002433333	pos

Lauroyl diethanolamide	HMDB0032358	288.253922	10.28636667	pos
L-beta-aspartyl-L-aspartic acid	HMDB0011163	249.072304	0.7678	pos
L-beta-aspartyl-L-glutamic acid	62013	307.078331	6.864716667	neg
L-beta-aspartyl-L-leucine	62015	247.129304	2.710183333	pos
L-Carnitine	34532	162.112768	0.749333333	pos
L-Citronellol glucoside	86545	341.194493	7.863983333	pos
L-Cysteinylglycine disulfide	5677	298.053178	0.69355	pos
L-Cystine	17	241.031536	0.69355	pos
L-Dopa	42	178.050501	5.032616667	neg
Leflunomide	HMDB0015229	288.095771	1.090316667	pos
Lenticin	HMDB0061115	247.144689	4.484716667	pos
Leptomycin B	44891	585.344461	12.18908333	neg
Lesquerolic acid	35494	371.281125	11.2542	neg
Letrozole	43225	308.091214	1.090316667	pos
LEUCOPTERIN	43937	178.035858	6.226183333	pos
Leucyl-leucine	68997	227.175838	4.160016667	pos
Leucyl-leucyl-norleucine	68993	340.259972	5.445566667	pos
Leucylproline	HMDB0011175	251.137052	4.055983333	pos
Leukotriene A4	3449	363.216306	10.29983333	neg
Leukotriene D4	3583	514.292411	10.22716667	pos
Leukotriene E4-d5	96401	887.535025	14.63321667	neg
Levan	HMDB0003539	522.204104	0.8856	pos
Levobunolol sulfate	935	352.122466	0.710516667	neg
L-Fucose	63169	145.049865	4.1062	neg
L-Fuculose	HMDB0060267	145.051272	15.54703333	neg
L-Glutamate	19	148.060742	0.749333333	pos
L-Glutamic acid dibutyl ester	3545	258.171665	6.61195	neg
L-Glutamine	18	129.066295	15.98305	pos
L-Hexahydro-3-imino-1,2,4-oxadiazepine-3-carboxyl	HMDB0030402	142.061466	15.98305	pos
L-Hexanoylcarnitine	3548	258.171613	5.516666667	neg
L-Histidine	21	156.07712	0.658466667	pos
L-Histidine trimethylbetaine	HMDB0029422	393.22733	9.9417	neg
L-Histidinol	HMDB0003431	142.097819	13.73791667	pos
L-Homocysteic acid	6545	221.982619	1.802133333	pos
Licochalcone A	LMPK12120424	377.113926	5.219016667	pos
Licoricone	47589	421.10384	4.526866667	pos
Lidocaine	995	235.180777	4.942016667	pos
Ligustilide	71484	208.133636	2.751633333	pos
Linoleamide	43435	297.290625	12.82123333	pos
Linoleic acid	HMDB0000673	281.24788	10.73525	pos
Linoleic Acid-biotin	45286	503.341302	11.6334	pos
Linoleoyl Ethanolamide	3718	346.272191	11.79395	pos
Linoleyl hydroxamic acid	LMFA08020211	318.241031	11.45178333	pos
Liothyronine	HMDB0000265	695.786175	15.22285	neg
Lisdexamfetamine	HMDB0015385	244.182881	7.518333333	neg
L-Isoleucine	23	132.102299	1.090316667	pos
Lithocholate 3-O-glucuronide	HMDB0002513	570.361577	9.71345	pos
Lithocholic acid	LMST04010003	394.330077	11.25935	pos
L-Leucine	24	132.102288	2.052666667	pos
L-Lysine	25	147.113119	0.622116667	pos
L-Menthyl acetoacetate	88396	285.171447	8.88765	neg
L-Methionine S-oxide	63430	148.042977	1.090316667	pos
Lodoxamide	1011	355.99417	5.158833333	neg
Longifolonine	67811	593.19549	12.08798333	neg
Lophirone J	52539	383.128464	11.29956667	pos

L-Ornithine	45121	133.097545	0.622116667	pos
Lotaustralin	HMDB0033865	262.129187	4.779033333	pos
LPA(0:0/16:0)	59309	391.226477	11.54023333	neg
LPA(0:0/18:2(9Z,12Z))	59311	457.234231	9.491233333	pos
L-Phenylalanine	HMDB0000159	210.077134	9.836516667	neg
L-Pipecolic acid	63100	130.086674	0.622116667	pos
L-Rhamnulose	HMDB0010207	209.066547	0.864066667	neg
L-Targinine	HMDB0029416	189.134991	0.712516667	pos
L-Tryptophan	33	203.082427	4.169433333	neg
Lubiminol	HMDB0029604	299.187106	9.246216667	neg
Lucidenic acid A	92454	439.24996	11.56056667	neg
Lucidenic acid D1	92933	515.229349	10.0047	neg
Lucidenic acid J	HMDB0035700	473.252455	16.00321667	pos
Lucidenic acid K	91094	473.252047	15.3814	pos
Lupinine	43880	214.144836	6.064333333	neg
Luteolin	3409	285.041199	5.68515	neg
L-Xylonate	HMDB0060256	147.02913	1.112466667	neg
LY171883	43454	317.161236	5.643033333	neg
LY255283	63069	359.208516	7.4129	neg
Lycomarasmine B	67021	322.0892	7.244233333	neg
Lycopene	LMPR01070257	575.398719	13.38231667	pos
Lysergic acid	66676	313.120444	6.338166667	neg
Lysergic acid diethylamide	66677	341.232851	9.391516667	pos
Lys-Gln-Ala-Gly-Asp-Val	85172	599.312593	4.779033333	pos
LysoPC(15:0)	HMDB0010381	480.310521	11.90591667	neg
LysoPC(16:0)	HMDB0010382	496.340942	11.07896667	pos
LysoPC(16:1(9Z)/0:0)	HMDB0010383	538.316202	10.38415	neg
LysoPC(17:0)	62785	554.347375	11.49943333	neg
LysoPC(18:0)	61694	524.37246	11.95526667	pos
LysoPC(18:1(11Z))	HMDB0010385	566.347407	11.15198333	neg
LysoPC(18:3(6Z,9Z,12Z))	HMDB0010387	540.307957	10.22716667	pos
LysoPC(18:3(9Z,12Z,15Z))	61698	556.280409	4.337966667	pos
LysoPC(20:3(5Z,8Z,11Z))	61703	546.356803	10.97813333	pos
LysoPC(20:4(5Z,8Z,11Z,14Z))	HMDB0010395	544.341152	10.71513333	pos
LysoPC(P-16:0)	61716	524.336803	11.31588333	neg
LysoPC(P-18:1(9Z))	HMDB0010408	506.361284	11.53246667	pos
LysoPE(0:0/18:2(9Z,12Z))	HMDB0011477	478.293959	10.6551	pos
LysoPE(0:0/18:3(9Z,12Z,15Z))	62267	520.267803	11.04893333	neg
LysoPE(0:0/20:3(11Z,14Z,17Z))	62272	504.309696	10.93775	pos
LysoPE(0:0/20:3(5Z,8Z,11Z))	62273	504.307321	11.89476667	pos
LysoPE(0:0/20:4(5Z,8Z,11Z,14Z))	HMDB0011487	546.282629	11.13138333	neg
LysoPE(0:0/20:4(8Z,11Z,14Z,17Z))	HMDB0011488	502.293925	10.695	pos
LysoPE(0:0/20:5(5Z,8Z,11Z,14Z,17Z))	HMDB0011489	544.266983	10.6567	neg
LysoPE(0:0/22:4(7Z,10Z,13Z,16Z))	HMDB0011493	530.325439	11.23941667	pos
LysoPE(0:0/22:5(4Z,7Z,10Z,13Z,16Z))	62282	526.294852	10.86301667	neg
LysoPE(0:0/22:5(7Z,10Z,13Z,16Z,19Z))	HMDB0011495	528.309888	10.85683333	pos
LysoPE(0:0/22:6(4Z,7Z,10Z,13Z,16Z,19Z))	HMDB0011496	526.294417	10.67518333	pos
LysoPE(18:2(9Z,12Z)/0:0)	HMDB0011507	476.279254	10.6567	neg
LysoPE(20:3(5Z,8Z,11Z)/0:0)	62300	502.295104	10.94575	neg
LysoPE(22:5(4Z,7Z,10Z,13Z,16Z)/0:0)	62308	528.30847	10.22716667	pos
LysoSM(d18:0)	HMDB0012082	484.385601	11.37315	pos
LysoSM(d18:1)	HMDB0006482	488.336332	12.09661667	pos
Lysyl-Asparagine	HMDB0028946	261.156158	0.622116667	pos
Lysyl-Glutamine	85851	292.198474	0.6755	pos
Lysyl-Histidine	85855	284.171014	4.75795	pos

MAHMA NONOate	62981	227.14694	9.125	pos
Malaoxon	1060	295.039984	4.1062	neg
Malonic acid	3237	207.014502	0.823166667	neg
Maltotriose	HMDB0001262	527.159692	0.8856	pos
Malvidin 3-(6-coumaroylglucoside)	HMDB0038012	657.203808	9.672883333	pos
Malvidin 3,7-di-(6-malonylglucoside)	LMPK12010406	810.181672	9.939183333	pos
Mandelonitrile rutinoside	88725	486.162791	10.17338333	neg
Mangalkanyl glucoside	91194	369.261914	12.82123333	pos
Mannan	HMDB0029931	711.2217	0.905383333	neg
Mannitol	HMDB0000765	205.068795	0.749333333	pos
Margaric acid	LMFA01010017	271.263684	0.002433333	pos
Matricin	41193	611.28723	11.49943333	neg
Maximaisoflavone C	47588	419.088292	5.6311	pos
m-Chlorohippuric acid	HMDB0001309	236.009528	0.786766667	pos
Medicagol	HMDB0033831	341.030292	1.112466667	neg
Megastigmatrienone	263585	191.143421	13.68475	pos
Melatonin glucuronide	HMDB0060830	431.141767	2.173566667	pos
Melithiazol A	71030	445.120933	0.484533333	pos
Melleolide	HMDB0035689	418.223189	9.473266667	pos
Menthol	HMDB0003352	174.185631	5.796033333	pos
Menthol propylene glycol carbonate	94334	539.357894	11.49206667	pos
Menthyl pyrrolidone carboxylate	HMDB0032368	301.201246	8.4576	pos
Mepenzolate	HMDB0015591	321.174939	9.9417	neg
Mepivacaine	43244	245.165789	8.950933333	neg
Merodesmosine	86951	385.245381	4.118683333	pos
Mesaconic acid	4130	131.034262	0.7301	pos
Mescaline	3346	229.155088	4.199916667	pos
Mesuagin	HMDB0035876	389.138133	7.622216667	neg
Metanephrine	65	180.102315	4.257983333	pos
METHACHOLINE	43545	160.133529	1.090316667	pos
Methamphetamine	2404	167.154669	15.98305	pos
Methanophenazine	63906	556.42492	15.49035	pos
Methantheline	85445	321.174681	12.49058333	neg
Methaqualone	66682	295.109622	9.520516667	neg
Methenamine	43250	141.113742	14.49081667	pos
Methicillin sodium	69751	419.089504	4.526916667	neg
Methionyl-Valine	HMDB0028986	266.152235	8.662066667	pos
Methohexital	HMDB0014617	263.138703	4.632166667	pos
Methoprene acid	43446	313.202934	8.972016667	neg
Methotrimeprazine	1189	327.154399	7.518333333	neg
Methoxamine	1198	229.155082	2.032866667	pos
methyl (+)-7-isojasmonate	LMFA02020016	269.139694	8.001833333	neg
Methyl (2E,6Z)-dodecadienoate	HMDB0031014	211.169675	0.98685	pos
Methyl (E)-2-dodecenoate	LMFA07010931	235.169753	0.002433333	pos
methyl (E)-2-methyl-4-((3aS)-1-methyl-5-oxo-2,3,5,8	264020	599.28741	11.37706667	neg
Methyl (R)-3-methyl-2-oxopentanoate	HMDB0037114	287.150716	7.2653	neg
Methyl (Z)-3-hexenoate	HMDB0031504	146.117897	1.090316667	pos
methyl 10,13-dihydroxy-9-oxo-11-octadecenoate	74528	365.230742	10.30701667	pos
methyl 13,15-epidioxy-12-hydroperoxy-9Z,16E-octa	LMFA01040043	339.215985	9.795466667	pos
methyl 13,15-epidioxy-16-hydroperoxy-9,11-octadec	LMFA01040041	357.227734	9.795466667	pos
methyl 13-sophorosyloxydocosanoate	LMFA13020002	677.446486	9.693166667	pos
Methyl 15-cyanopentadecanoate	HMDB0032791	282.243312	10.28636667	pos
methyl 15-hydroperoxy-9Z,12Z,16E-octadecatrienoa	74464	342.264538	10.32751667	pos
Methyl 2,6-dihydroxy-4-quinolinecarboxylate	93963	419.089466	4.758616667	neg
Methyl 2E-hexenoate	HMDB0031500	255.160653	6.864716667	neg

Methyl 3-(2,3-dihydroxy-3-methylbutyl)-4-hydroxyber	HMDB0032796	277.105379	15.2193	pos
Methyl 3-methylbutanoate	HMDB0030027	231.160422	6.949	neg
Methyl 4-chloro-1H-indole-3-acetate	HMDB0032937	246.030113	0.622116667	pos
Methyl 4-phenylbutanoate	HMDB0036385	177.09161	9.035283333	neg
methyl 8-[2-(2-formyl-vinyl)-3-hydroxy-5-oxo-cyclope	74529	311.185766	9.836716667	pos
methyl 8-[3,5-epidioxy-2-(3-hydroperoxy-1-pentenyl)	74479	441.250479	9.899633333	neg
methyl 9,12-dihydroxy-13-oxo-10-octadecenoate	74527	325.237798	10.695	pos
methyl 9-hydroperoxy-10,12-epidioxy-13,15-octadec	LMFA01040040	357.227645	9.3094	pos
Methyl acrylate-divinylbenzene, completely hydrolyz	HMDB0032389	397.108896	10.21556667	neg
Methyl bixin/ (Bixin dimethyl ester)	41462	447.194194	13.04023333	pos
Methyl caprylate	87561	159.138276	0.463416667	pos
Methyl DL-Leucate	LMFA07010959	129.091405	0.98685	pos
Methyl geranate	265098	165.127778	4.484716667	pos
Methyl helianthenoate F glucoside	95262	335.112041	9.667733333	neg
Methyl jasmonate	LMFA02020010	447.275785	10.61491667	neg
Methyl tetradecanoate	LMFA07010467	243.232203	0.002433333	pos
Methyl trans-p-methoxycinnamate	HMDB0030752	237.077111	5.727266667	neg
Methyl viologen	65511	511.101964	10.21556667	neg
methyl-10-hydroperoxy-8E,12Z,15Z-octadecatrienoa	74463	325.237815	10.4711	pos
Methylenediurea	66388	155.054197	14.738	pos
Methylgallic acid-O-sulphate	HMDB0060005	265.001326	0.866166667	pos
Methylimidazole acetaldehyde	58224	142.097862	15.98305	pos
Methylphenidate	1227	251.175843	6.000516667	pos
Methylscopolamine	HMDB0014605	363.169928	9.62565	neg
Methylthiobenzoylglycine	2540	421.052732	4.211566667	neg
Methysergide	HMDB0014392	336.208593	3.827083333	pos
Metrizamide	HMDB0015518	787.847231	13.65406667	neg
Metrizoic acid	69844	672.782715	14.74585	neg
Metronidazole	573	154.061497	15.94263333	pos
MG(0:0/14:0/0:0)	62314	325.236633	10.99823333	pos
MG(0:0/18:1(9Z)/0:0)	62321	374.327186	10.20713333	pos
MG(0:0/18:3(6Z,9Z,12Z)/0:0)	62323	397.260223	10.51023333	neg
MG(14:0/0:0/0:0)	62344	285.24288	0.002433333	pos
MG(16:0/0:0/0:0)	3855	353.267009	11.8746	pos
MG(16:1(9Z)/0:0/0:0)	62347	373.260376	10.27876667	neg
MG(22:2(13Z,16Z)/0:0/0:0)	62365	428.374396	11.13923333	pos
MGDG(18:3(9Z,12Z,15Z)/18:3(9Z,12Z,15Z))	75584	792.565698	11.27946667	pos
MGDG(20:5(5Z,8Z,11Z,14Z,17Z)/18:3(9Z,12Z,15Z))	46649	781.528526	12.19763333	pos
m-Hydroxycarvedilol sulfate	1569	501.13553	10.73845	neg
Mianserin 2-oxide	1273	303.147634	0.641016667	pos
Mifepristone	HMDB0014972	474.263556	10.23661667	neg
Minoxidil	1315	210.134017	0.7301	pos
Mitomycin	590	333.119845	5.116983333	neg
Mizolastine	HMDB0240233	433.215916	8.762433333	pos
Mollicellin D	89190	403.094543	7.7066	neg
Momordol	HMDB0029804	458.385118	11.21971667	pos
Monoethylglycinexylidide	HMDB0060656	411.276107	10.63578333	neg
Monoethylglycylxylidide (MEGX)	996	207.149599	4.65325	pos
Monomenthyl succinate	91299	301.166348	8.044016667	neg
m-Trifluoromethylhippuric acid	2682	246.038809	5.053716667	neg
Mulberrofuran T	91126	699.292954	9.391516667	pos
Muricatacin	LMFA05000682	329.234168	10.21556667	neg
Murrayacinine	HMDB0030224	689.34097	0.6532	neg
Mycalamide A	71498	521.306269	4.821233333	pos
Mycinamicin VI	71017	648.376018	13.25091667	neg

Mycinamicin VIII	71019	506.348744	0.846116667	pos
myo-Inositol	144	179.055619	0.76405	neg
Myricolal	89121	479.329852	9.7543	pos
Myrigalon B	52293	318.170872	0.02505	pos
Myriocin	LMSP01080020	402.285857	10.14556667	pos
Myristic acid	LMFA01010014	246.243212	7.208483333	pos
Myrtine	86923	185.165271	7.003433333	pos
N-(1-Deoxy-1-fructosyl)leucine	HMDB0037840	294.155299	2.15345	pos
N-(1-Deoxy-1-fructosyl)methionine	HMDB0037841	350.06754	7.044466667	pos
N-(1-Deoxy-1-fructosyl)valine	HMDB0037844	262.129129	1.090316667	pos
N-(2'-(4-benzenesulfonamide)-ethyl) arachidonoyl a	36721	485.283731	10.0258	neg
N-(3S-hydroxydecanoyl)-L-serine	45741	276.181125	6.20565	pos
N-(6-aminohexanoyl)-6-aminohexanoic acid	45748	227.175828	4.5058	pos
N-(9,12-octadecadienoyl)-glutamic acid	LMFA08020217	427.315383	11.27946667	pos
N-(p-Hydroxyphenethyl)actinidine	86906	249.152718	10.34203333	neg
N,2,3-Trimethyl-2-(1-methylethyl)butanamide	91344	172.169899	7.7206	pos
N,N-bis(4-acetamidobutyl)-15-methylhexadec-6E-er	LMFA08020234	532.385658	11.47181667	pos
N,N'-Bis(gamma-glutamyl)cystine	93146	499.116234	10.22716667	pos
N,N'-Diacetylbenzidine	72940	313.120595	6.064333333	neg
N,N'-Diacetylhydrazine	HMDB0060496	139.048338	15.90231667	pos
N,N'-Dicyclohexylurea	34503	225.196557	9.0643	pos
N,N-dimethylhistidine	44783	365.195382	10.594	neg
N,N-dimethyl-Safingol	53956	330.337255	10.49155	pos
N,N-Dimethylsphingosine	34487	310.311086	12.88041667	pos
N,O-Didesmethylvenlafaxine	3007	232.170154	6.021116667	pos
N-[(3a,5b,7b)-7-hydroxy-24-oxo-3-(sulfooxy)cholan-2	6670	510.254184	8.128366667	neg
N-[(diphenylmethoxy)acetyl]-Glutamine	2322	415.152001	6.654066667	neg
N1-Acetylspermine	3369	283.190635	9.959816667	pos
N1-Methyl-2-pyridone-5-carboxamide	HMDB0004193	153.066183	2.194133333	pos
N1-Methyl-4-pyridone-3-carboxamide	HMDB0004194	170.092987	15.98305	pos
N2-(ADP-D-Ribosyl)-L-arginine	6115	716.178499	4.7158	pos
N2-(D-1-Carboxyethyl)-L-lysine	63467	219.134455	1.090316667	pos
N2-Acetyl-L-aminoadipyl-δ-phosphate	63445	264.028603	1.297583333	neg
N2-Acetyl-L-ornithine	3303	175.108242	0.905683333	pos
N2-Succinyl-L-glutamic acid 5-semialdehyde	HMDB0001180	249.10853	0.905683333	pos
N6-Acetyl-L-lysine	HMDB0000206	187.108462	1.09225	neg
N6-Carbamoyl-L-threonyladenosine	95993	411.127963	11.80461667	neg
N-Acetoxy-4-aminobiphenyl	66010	245.128926	5.115883333	pos
N-Acetyl-7-O-acetylneuraminic acid	5751	396.115396	1.029866667	neg
N-Acetyl-a-neuraminic acid	HMDB0000773	290.088865	1.09225	neg
N-Acetylaspartylglutamic acid	HMDB0001067	327.078774	11.05878333	pos
N-Acetyldemethylphosphinothricin	63587	415.068123	7.896366667	neg
N-Acetyl-D-glucosamine	HMDB0000215	244.079518	0.846116667	pos
N-Acetyl-D-glucosaminyldiphosphodolichol	6251	632.225718	4.653283333	neg
N-Acetyldjenkolic acid	HMDB0029421	277.033543	5.13805	neg
N-Acetyl-DL-methionine	62466	190.054029	4.3584	neg
N-Acetyl-D-mannosamine 6-phosphate	HMDB0001121	300.049509	0.74595	neg
N-Acetylhistamine	HMDB0013253	154.097847	15.98305	pos
N-Acetyl-leu-leu-tyr-amide	68969	471.257433	10.89728333	pos
N-Acetyl-L-glutamate 5-semialdehyde	3298	156.065792	0.548016667	pos
N-Acetyl-L-methionine	HMDB0011745	214.051631	0.866166667	pos
N-Acetylmannosamine	HMDB0001129	204.087134	0.846116667	pos
N-Acetylmuramic acid 6-phosphate	63225	745.148099	9.646666667	neg
N-Acetylmuramoyl-Ala	65878	363.141158	7.875283333	neg
N-Acetylneuraminic Acid	24101	310.113801	0.8261	pos

N-Acetylneuraminic acid 9-phosphate	HMDB0004381	372.068165	0.806416667	pos
N-Acetylprocainamide	HMDB0041944	295.211952	5.672333333	pos
N-Acetylserotonin	366	263.104314	4.779683333	neg
NAD	HMDB0000902	709.113788	1.09225	neg
Nandrolone phenpropionate	HMDB0015119	424.283311	13.34593333	pos
Naphazoline hydrochloride	66760	228.080575	1.090316667	pos
Naphthalene	HMDB0029751	146.096756	9.878016667	pos
N-Arachidonoyl Dopamine-d8	96395	430.353785	10.83661667	pos
N-arachidonoyl tyrosine	LMFA08020076	512.300356	10.1101	neg
Narcotoline	67832	417.166514	6.654433333	pos
N-benzyl-1-methyl-1H-pyrazolo[3,4-d]pyrimidin-4-an	96288	240.124785	4.800116667	pos
Nb-Lignoceroyltryptamine	HMDB0040819	549.417428	14.96886667	pos
Nb-Palmitoyltryptamine	95277	421.318199	10.04253333	pos
N-butanoyl-lhomoserine lactone	LMFA08030002	189.12379	1.090316667	pos
N-Butyl-beta-carboline-3-carboxylate	69712	313.120281	7.53855	neg
N-Butylscopolamine metabolite	2399	256.155981	5.7905	neg
n-butyrl n-valeric acid	LMFA01060025	217.108184	6.4856	neg
N-Caffeoyltryptophan	86535	389.112488	1.844116667	pos
N-Cyclohexanecarbonylpentadecylamine	45078	360.324277	15.80033333	pos
N-Cyclopropylammelide	69836	151.061774	15.98305	pos
N-Cyclopropyl-trans-2-cis-6-nonadienamide	88262	211.180881	8.3962	pos
N-Decanoylglycine	HMDB0013267	230.17556	6.75715	pos
N-Deisopropyl-fluvastatin	HMDB0014038	350.120433	8.972016667	neg
N-Desalkyl flurazepam	HMDB0061161	327.008754	9.878016667	pos
N-Desethylquinagolide sulfate	2214	446.141835	9.62565	neg
N-Desmethylcitalopram	HMDB0014021	619.290094	11.8654	neg
N-Desmethylritonavir	HMDB0060896	687.277577	11.88563333	neg
N-Despropyl-rotigotine	HMDB0060841	254.101518	5.0748	neg
N-docosahexaenoyl GABA	75487	414.300871	9.473266667	pos
N-dodecanoyl-L-Homoserine lactone	64717	328.213675	8.022933333	neg
Nebivolol	85541	809.343617	13.69026667	neg
Necatorine	93641	309.05193	8.5924	neg
Neobetanin	HMDB0029405	593.124969	12.49058333	neg
Neoilludin A	73450	321.131669	14.66688333	pos
Neopellitorine A	HMDB0037770	252.136525	7.700116667	pos
Neosaxitoxin	71608	314.122927	8.1073	neg
Neostigmine	1490	443.266127	10.27876667	neg
Nepetariaside	HMDB0039014	329.16132	5.916916667	neg
Nerolidol	LMPR0103010005	205.195471	0.002433333	pos
Neryl glucoside	HMDB0029346	317.196381	11.33671667	pos
Neryl propionate	LMFA07010976	211.16967	1.823116667	pos
Netilmicin	HMDB0015090	476.30767	4.995966667	pos
Neurosporene	HMDB0003114	577.414445	11.45178333	pos
N-Formyl-4-amino-5-aminomethyl-2-methylpyrimidir	73404	167.093145	15.94263333	pos
N-Formyl-L-glutamic acid	HMDB0003470	156.030843	14.3939	neg
N-gamma-L-Glutamyl-D-alanine	HMDB0036301	219.097891	1.090316667	pos
n-heptanoyl acetic acid	35717	343.213382	8.824366667	neg
N-Hexadecanoylpyrrolidine	HMDB0032740	327.337681	10.89728333	pos
N-Hydroxy-4-aminobiphenyl	66011	369.159832	12.51056667	neg
N-Hydroxyriluzole	2333	232.998645	0.806416667	pos
Niacinamide	HMDB0001406	243.087764	5.221616667	neg
Niazicin A	HMDB0038952	424.08238	0.566216667	pos
Niazimicin A	HMDB0031944	396.08742	0.566216667	pos
Niazirinin	88730	339.154858	9.632383333	pos
Nicotine-1'-N-oxide	HMDB0001497	355.213481	8.950933333	neg

Nicotinuric acid	1499	163.050506	0.566216667	pos
Nicotyrine	68165	176.118615	15.82111667	pos
Nifuradene	72990	242.087513	0.905683333	pos
Nilotinib	45263	528.178727	10.594	neg
N-isobutyl-2E,4Z-octadecadienoyl amine	LMFA08020207	336.326602	13.3276	pos
Nitarsone	HMDB0031822	264.979134	15.90231667	pos
NITRENDIPINE	44354	359.125734	5.57985	neg
Nitroglycerin	HMDB0014865	452.998949	10.34203333	neg
Nitroglycerine	1578	272.000713	0.884666667	neg
Nitroxoline	HMDB0015491	208.069804	0.548016667	pos
N-Jasmonoylisoleucine	86237	324.21759	0.566216667	pos
N-Lauroylglycine	HMDB0013272	258.206836	8.334716667	pos
N-linolenoyl dopamine	LMFA08020271	431.325156	10.4506	pos
N-Methoxyspirobrassinol methyl ether	HMDB0041059	297.071636	8.5806	pos
N-Methyl-2,3,7,8-tetramethoxy-5,6-dihydrobenzophe	69374	468.168099	14.8218	neg
N-Methylaspidospermatidine	86911	303.183943	4.097783333	pos
N-Methyl-N,4-dinitrosoaniline	73146	183.088076	15.98305	pos
n-methylnicotinamide	3770	271.119419	5.348033333	neg
N-methylphenylalanine	86103	180.102323	5.301516667	pos
N-Methyltyramine	58167	134.096785	5.404333333	pos
N-Nitroso-N-morpholinoaminoacetonitrile	1338	153.07736	0.045716667	pos
N-Nitrosoproline	HMDB0041946	167.043126	15.90231667	pos
N-Nitrosothiazolidine-4-carboxylic acid	94727	200.972763	1.823116667	pos
N-Nitrosotomatidine	89718	445.340832	10.73525	pos
N-Nonanoylglycine	HMDB0013279	216.159921	6.08295	pos
Nogalamycin	72425	770.298008	9.672883333	pos
N-oleoyl tyrosine	LMFA08020099	468.309586	10.28636667	pos
NONOXYNOL-9	43278	634.452932	11.97545	pos
Nonyl octanoate	HMDB0034131	271.263623	1.9283	pos
Norgestimate	1606	392.218719	0.786766667	pos
Norharman	4172	335.129526	10.94575	neg
Normeperidine	1132	251.175763	4.179616667	pos
Norophthalmic acid	58301	258.108987	1.090316667	pos
Novobiocin	HMDB0015185	613.239406	10.12496667	pos
N-palmitoyl alanine	75505	350.267193	12.05626667	pos
N-palmitoyl glutamic acid	75469	408.270544	5.979883333	pos
N-palmitoyl leucine	LMFA08020115	392.314005	11.8343	pos
N-palmitoyl phenylalanine	75473	448.307904	9.7099	neg
N-palmitoyl threonine	LMFA08020107	380.27782	12.82123333	pos
N-palmitoyl valine	75502	378.298633	12.94041667	pos
N-pentacosanoyl taurine	LMFA08020253	977.76793	15.48823333	neg
N-Phenylacetyl pyroglutamic acid	HMDB0059782	290.103487	0.710516667	neg
N-Phosphohypotaurocyamine	66013	214.004058	0.92605	pos
N-physeteroyl lysine	LMFA08020263	393.250796	5.528016667	pos
N-Salicyloylaspartic acid	94075	271.092924	2.3174	pos
N-stearoyl glutamic acid	75472	414.322226	10.95795	pos
N-stearoyl proline	LMFA08020119	404.31435	13.11826667	pos
N-stearoyl valine	LMFA08020122	406.329925	13.82548333	pos
N-Trimethyl-2-aminoethylphosphonate	63594	333.136067	11.56056667	neg
N-Undecanoylglycine	HMDB0013286	244.191225	7.515733333	pos
N-Undecylbenzenesulfonic acid	88521	311.169306	13.08056667	neg
n-valeryl acetic acid	LMFA01060012	287.150694	7.938566667	neg
NVP-AEW541	45579	478.199936	4.219483333	pos
O-(17-carboxyheptadecanoyl)carnitine	LMFA07070085	458.348624	10.9175	pos
O-butanoyl-carnitine	LMFA07070003	232.154801	3.8063	pos

Octadecanedioic acid	LMFA01170029	297.24295	10.32751667	pos
Octadecyl fumarate	92842	386.327207	10.99823333	pos
Octaethylene glycol	HMDB0094680	388.254863	4.800116667	pos
Octafluorocyclobutane	HMDB0031292	244.985832	5.643033333	neg
Octahydro-6-isopropyl-2(1H)-naphthalenone	91204	212.201318	5.589866667	pos
Octyl phenylacetate	HMDB0037713	249.18533	0.002433333	pos
Octyl propanoate	LMFA07010983	169.159065	0.946233333	pos
O-decanoyl-R-carnitine	36669	316.248807	8.007216667	pos
O-Desacetylcephalothin	1639	372.066733	9.878016667	pos
O-Desmethylquinine glucuronide	2238	531.199455	7.790966667	neg
O-Desmethylvenlafaxine glucuronide	HMDB0061172	429.221145	10.73525	pos
O-hexanoyl-R-carnitine	LMFA07070001	258.171658	6.14865	neg
OHHdiA-PE	LMGP20020043	634.3354	11.15198333	neg
OHODA-PA	LMGP20070035	669.371428	0.641016667	pos
Okanin 3,4,3'-trimethyl ether 4'-glucoside	LMPK12120174	537.160166	10.17338333	neg
OKHdiA-PE	LMGP20020041	632.319505	10.67736667	neg
OKODA-PI	LMGP20050033	787.4078	12.59033333	neg
OKOOA-PS	LMGP20040029	656.319864	10.7181	neg
Oleamide	HMDB0002117	299.306218	10.34803333	pos
OLEANANOIC ACID ACETATE	44453	481.366218	11.04893333	neg
Oleandolide	40995	385.223958	8.170533333	neg
oleanolic acid	LMPR0106150004	437.343593	15.05126667	neg
Oleic Acid	190	327.254804	11.56056667	neg
Oleyl alcohol	HMDB0029632	269.284733	0.002433333	pos
Oleyl Anilide	44935	396.265375	7.31095	pos
Oltipraz	HMDB0041967	224.963047	15.8641	neg
omega-hydroxy myristic acid	35422	262.238295	7.372416667	pos
ONO-8711	96519	462.147591	0.8856	pos
OOB-PE	LMGP20020034	608.319527	11.0901	neg
O-octanoyl-R-carnitine	36665	288.217525	6.490233333	pos
Ophiopogonin C'	HMDB0029312	761.383895	4.4215	pos
O-Phosphoethanolamine	HMDB0000224	142.026745	0.69355	pos
O-Phosphohomoserine	HMDB0003484	397.041871	4.88505	neg
O-Phosphothreonine	HMDB0011185	244.02295	0.74595	neg
Ophthalmic acid	58300	290.135111	1.090316667	pos
Orotidine	5754	333.05853	1.1528	neg
Ortho-Hydroxyphenylacetic acid	HMDB0000669	135.044434	0.92605	pos
Oryzalide A	92438	321.205981	9.45315	pos
O-sebacoylcarnitine	LMFA07070100	346.222926	7.904866667	pos
OSU03012	96406	459.143866	10.17338333	neg
O-Succinyl-L-homoserine	362	200.056196	1.112466667	neg
OTK	64851	357.236387	5.404333333	pos
Ouabain	HMDB0015224	585.288979	4.055983333	pos
O-Ureidohomoserine	62904	353.14334	12.86691667	neg
Ovalifolin	48495	385.082552	8.762433333	pos
Ovalitenone	LMPK12120372	356.114387	7.24955	pos
Oxalic acid	HMDB0002329	178.982711	1.98845	neg
Oxaloglutarate	3339	249.022391	0.710516667	neg
Oxalosuccinic acid	HMDB0003974	235.008763	1.09225	neg
Oxidized glutathione	HMDB0003337	611.145874	1.09225	neg
Oxoadipic acid	HMDB0000225	143.034281	0.92605	pos
Oxomefruside	1108	395.015977	4.927216667	neg
OXONITINE	43770	690.278217	11.56056667	neg
Oxotolrestat	2881	380.052071	0.69355	pos
Oxydisulfoton	72760	271.004558	0.710516667	neg

Oxypinnatanine	HMDB0029403	305.098939	7.244233333	neg
PA(12:0/15:0)	LMGP10010045	623.394259	13.5268	neg
PA(13:0/18:3(6Z,9Z,12Z))	LMGP10010077	611.407836	9.71345	pos
PA(13:0/18:4(6Z,9Z,12Z,15Z))	81245	627.404137	13.15791667	pos
PA(14:0/0:0)	40945	365.208226	3.661516667	pos
PA(14:0/13:0)	81257	623.394437	15.82238333	neg
PA(14:0/18:3(6Z,9Z,12Z))	81263	625.423388	10.32751667	pos
PA(14:1(9Z)/0:0)	LMGP10050038	398.227663	11.0386	pos
PA(14:1(9Z)/16:1(9Z))	LMGP10010113	597.391609	0.6532	neg
PA(14:1(9Z)/18:4(6Z,9Z,12Z,15Z))	81288	677.358012	0.641016667	pos
PA(14:1(9Z)/20:5(5Z,8Z,11Z,14Z,17Z))	LMGP10010130	665.420572	9.980383333	pos
PA(15:0/18:4(6Z,9Z,12Z,15Z))	81312	655.434034	9.693166667	pos
PA(15:0/22:4(7Z,10Z,13Z,16Z))	HMDB0114827	709.484151	15.1467	neg
PA(16:0/16:0)	HMDB0000674	687.433311	9.980383333	pos
PA(16:1(9Z)/18:3(6Z,9Z,12Z))	81376	669.449525	10.28636667	pos
PA(18:1(9Z)/20:5(5Z,8Z,11Z,14Z,17Z))	82044	759.435161	15.90231667	pos
PA(18:1(9Z)/22:2(13Z,16Z))	LMGP10010336	737.550152	14.2296	pos
PA(18:2(9Z,12Z)/14:0)	LMGP10010340	627.440294	16.00321667	pos
PA(20:4(5Z,8Z,11Z,14Z)/0:0)	46749	459.250167	9.473266667	pos
PA(21:4(6Z,9Z,12Z,15Z)/0:0)	40941	512.276392	9.473266667	pos
PA(22:0/0:0)	LMGP10050031	495.344399	12.54061667	pos
PA(22:6(4Z,7Z,10Z,13Z,16Z,19Z)/0:0)	82338	500.278138	10.30701667	pos
PA(P-20:0/0:0)	LMGP10070001	451.317962	12.60105	pos
p-Acetamidophenol (Acetaminophen, Tylenol)	487	152.070922	3.8686	pos
Palmitaldehyde	6317	258.279781	9.939183333	pos
Palmitic acid	187	257.247924	0.002433333	pos
Palmitic amide	HMDB0012273	256.263944	12.82123333	pos
Palmitoleamide	LMFA08010010	298.239459	10.27876667	neg
Palmitoleic acid	HMDB0003229	255.23239	1.970383333	pos
Palmitoleoyl-EA	46564	298.274742	11.57286667	pos
Palmitoyl Serinol	HMDB0013654	330.301001	10.24565	pos
Palmitoylcarnitine	LMFA07070004	400.342924	10.71513333	pos
Palmitoylethanolamide	HMDB0002100	322.272314	11.1996	pos
Pamidronate	HMDB0014427	233.993427	0.6902	neg
Panaquinquecol 2	HMDB0038938	277.180455	10.71513333	pos
Panaquinquecol 7	LMFA05000688	277.180487	8.150466667	pos
Pandamarilactam 3x	89412	280.119631	6.696183333	neg
Pantetheine	HMDB0003426	317.093923	9.672883333	pos
Pantetheine 4'-phosphate	HMDB0001416	357.088816	6.085383333	neg
Pantothenic acid	HMDB0000210	218.103419	3.663416667	neg
Pantothenol	HMDB0004231	228.121055	6.226183333	pos
Paraoxon	HMDB0013035	314.017729	0.622116667	pos
Paraxanthine	HMDB0001860	181.072355	3.827083333	pos
Patellamide A	71065	787.324304	14.28501667	neg
PC(0:0/5:0)[U]	40358	342.168262	4.4215	pos
PC(13:0/0:0)	40267	454.293753	10.89728333	pos
PC(14:0/14:1(9Z))	59317	698.4699	10.51208333	pos
PC(15:0/15:0)	59384	744.491513	13.75545	pos
PC(16:0/0:0)[rac]	LMGP01050113	496.340931	10.93775	pos
PC(16:0/0:0)[U]	182	540.331863	10.92508333	neg
PC(16:0/14:0)	59415	706.537943	13.25368333	pos
PC(16:1(9Z)/14:1(9Z))	59447	740.463424	5.466183333	pos
PC(17:2(9Z,12Z)/0:0)	76569	504.310812	11.3363	neg
PC(18:1(9Z)/0:0)[U]	184	566.34752	11.2953	neg
PC(18:1(9Z)/4:0)	LMGP01010916	574.386732	10.34803333	pos

PC(18:1(9Z)/P-16:0)	59572	788.580421	12.90616667	neg
PC(18:2(2E,4E)/0:0)	LMGP01050034	564.331895	10.67736667	neg
PC(18:2(9Z,12Z)/15:0)	59578	766.536166	11.91493333	pos
PC(20:1(11Z)/P-18:1(9Z))	59773	836.592461	11.27946667	pos
PC(21:4(6Z,9Z,12Z,15Z)/0:0)	40270	575.379212	8.80145	pos
PC(22:4(7Z,10Z,13Z,16Z)/0:0)	LMGP01050124	589.395183	9.71345	pos
PC(24:0/25:0)[U]	39902	970.81847	13.43565	neg
PC(24:2(5Z,9Z)/24:2(5Z,9Z))[U]	39908	932.749444	14.5433	pos
PC(4:0/4:0)	39956	793.363631	13.49036667	neg
PC(8:2(2E,4E)/8:2(2E,4E))	LMGP01011254	502.255824	9.473266667	pos
PC(9:0/0:0)	LMGP01050068	420.213724	7.495216667	pos
PC(O-10:1(9E)/0:0)	LMGP01060027	396.251874	12.82123333	pos
PC(O-11:1(10E)/2:0)	4078	452.278177	10.36856667	pos
PC(O-15:0/0:0)	40383	506.300429	5.23965	pos
PC(O-16:0/2:0)	40075	562.326562	12.56076667	pos
PC(O-5:0/0:0)[R]	40394	345.213766	3.993383333	pos
PC(P-15:0/0:0)	40399	466.330037	12.19763333	pos
p-CHLOROPHENYLALANINE	44308	200.04771	4.219483333	pos
p-Cresol sulfate	HMDB0011635	187.006667	5.179716667	neg
p-Cymene	HMDB0005805	179.107292	8.5924	neg
PE(13:0/13:0)	40640	646.386847	4.219483333	pos
PE(13:0/18:4(6Z,9Z,12Z,15Z))	76629	708.400898	6.449583333	pos
PE(14:0/18:3(6Z,9Z,12Z))	60274	724.432115	5.857883333	pos
PE(14:1(9Z)/15:0)	60300	670.438726	9.350416667	pos
PE(14:1(9Z)/16:0)	60301	684.454419	9.980383333	pos
PE(16:0/0:0)	40776	454.293846	11.05878333	pos
PE(17:2(9Z,12Z)/0:0)	77682	486.260909	9.491233333	pos
PE(18:0/0:0)	LMGP02050001	482.32521	11.91493333	pos
PE(18:1(9Z)/0:0)	40778	478.294917	11.13138333	neg
PE(18:1(9Z)/18:4(6Z,9Z,12Z,15Z))	60476	782.497157	13.15671667	neg
PE(18:2(9Z,12Z)/0:0)	77677	522.284611	11.6013	neg
PE(20:1(11Z)/14:1(9Z))	60647	716.522416	13.6498	pos
PE(20:2(11Z,14Z)/14:0)	60679	714.509419	13.5268	neg
PE(20:4(5Z,8Z,11Z,14Z)/0:0)	LMGP02050009	500.279496	10.67736667	neg
PE(P-16:0/0:0)	46719	436.284229	11.27475	neg
Pectachol	93670	460.270139	9.491233333	pos
Pefloxacin N-oxide	HMDB0060613	350.150947	9.391516667	pos
Pelargonidin	LMPK12010003	270.054505	4.253166667	neg
Pelargonidin 3,5-di-(6-acetylglucoside)	LMPK12010045	660.169182	9.646666667	neg
Pelargonidin 3-sophoroside	HMDB0033679	616.177204	9.672883333	pos
Pemirolast	85441	273.075165	8.5924	neg
Penciclovir	2651	254.125286	1.090316667	pos
Penicillenic acid	69194	283.013986	2.895883333	pos
Penicillin G	1735	379.097104	4.948316667	neg
Penicillin V	1739	373.081655	0.02505	pos
Penicilloic acid of 5-OH	2706	486.056698	5.7905	neg
Penicilloic G acid	1737	353.117191	5.569266667	pos
pentadecanal	75359	244.264003	9.0643	pos
Pentadecanedioic acid	LMFA01170021	295.191116	10.20713333	pos
Pentadecanoylcarnitine	HMDB0062517	408.30918	13.50863333	pos
Pentadecylic acid(d3)	45753	284.207372	10.57348333	pos
Pentasine	HMDB0029803	634.335262	11.2953	neg
Pentigetide	92950	589.260238	3.993383333	pos
Pentosidine	58186	423.198833	9.75215	neg
Pentyl acetate	HMDB0039095	131.107064	0.4423	pos

Pentyl butanoate	89748	157.122753	7.833116667	neg
Pentyl octanoate	LMFA07010992	197.19043	12.54061667	pos
Peonidin acetyl 3,5-diglucoside	LMPK12010248	650.182694	6.6133	pos
Pepsinostreptin	69131	654.444061	10.57348333	pos
Perfluorooctanesulfonic acid	HMDB0059586	498.93006	11.76396667	neg
Perfluorooctanoic acid	96224	412.967469	9.77325	neg
Perilla sugar	90918	148.112402	5.055316667	pos
Perillic acid	HMDB0004586	167.106991	0.548016667	pos
Perilloside C	HMDB0040563	299.183876	8.211883333	pos
Perillyl aldehyde	LMPR0102090010	168.138677	5.404333333	pos
Perindoprilat glucuronide	1797	515.222501	10.92508333	neg
Persenone A	HMDB0036568	361.274312	10.695	pos
Persin	36469	425.29179	11.47903333	neg
p-FLUOROPHENYLALANINE	44126	184.077134	3.599566667	pos
PG(12:0/13:0)	3874	663.365326	4.8634	pos
PG(13:0/20:3(8Z,11Z,14Z))	78910	713.475074	10.22716667	pos
PG(13:0/20:5(5Z,8Z,11Z,14Z,17Z))	78912	709.446389	9.959816667	pos
PG(14:0/18:3(9Z,12Z,15Z))	79752	699.459465	9.693166667	pos
PG(16:1(9Z)/18:2(9Z,12Z))	LMGP04010901	727.490411	10.75555	pos
PG(16:1(9Z)/18:3(9Z,12Z,15Z))	61867	743.485582	9.672883333	pos
PG(17:2(9Z,12Z)/18:2(9Z,12Z))	79115	757.500648	10.18661667	pos
PG(21:0/22:0)	LMGP04010698	894.719269	4.7158	pos
PG(22:1(11Z)/0:0)	LMGP04050026	584.390015	10.12496667	pos
PG(O-16:0/21:0)	LMGP04020014	817.575137	13.9131	pos
PG(O-18:0/0:0)	LMGP04060002	516.367356	10.04253333	pos
PG(O-18:0/19:0)	LMGP04020032	817.574765	13.72018333	pos
PG(P-20:0/22:6(4Z,7Z,10Z,13Z,16Z,19Z))	LMGP04030093	817.574997	15.3814	pos
PGA2	LMFA03010035	379.211244	9.478266667	neg
PGDM-d4	96496	377.176421	11.66235	neg
PGE1 Alcohol	36124	358.295857	10.40958333	pos
PGF1a alcohol	LMFA03010173	387.276115	9.77325	neg
PGF1alpha	3503	357.264309	10.59398333	pos
PGF1alpha Alcohol	36220	387.276205	10.2577	neg
PGF1beta	LMFA03010069	355.249908	10.594	neg
PGF2alpha	LMFA03010002	337.237152	10.63491667	pos
PGF2alpha alcohol	LMFA03010113	341.269332	11.57286667	pos
PGF2alpha-11-acetate methyl ester	LMFA03010097	411.274059	10.4506	pos
PGG2	LMFA03010009	349.200516	10.06786667	neg
PGH2	LMFA03010010	397.222638	7.769866667	neg
PGP(i-12:0/i-12:0)	HMDB0116536	689.345698	14.1947	neg
PG-PI	LMGP20050015	723.31405	12.72153333	pos
Phaclofen	374	294.030344	2.885916667	neg
Phalluside-1	LMSP05010039	764.508204	10.43013333	pos
Phaseolic acid	HMDB0031897	243.124124	5.8116	neg
Phenanthrene-3,4-diol	65920	255.066854	7.370716667	neg
Phencyclidine	66688	282.161346	5.424933333	pos
Phenformin	HMDB0015050	206.140543	15.98305	pos
Phenicarbazide	73190	169.1088	15.98305	pos
Phenol sulphate	HMDB0060015	172.990899	4.2741	neg
Phenolic phosphate	65822	154.989836	0.573266667	neg
Phentolamine	1839	299.186124	9.044266667	pos
Phenyl glucuronide	1825	539.139501	0.884666667	neg
Phenylacetic acid	129	154.086578	4.358683333	pos
Phenylacetylglycine	HMDB0000821	238.072413	9.267316667	neg
Phenylamil	69683	304.071494	0.7282	neg

Phenylethanolaminium	HMDB0062626	177.055011	1.970383333	pos
Phenylglyoxylic acid	58059	168.06586	0.548016667	pos
Phenylpropanolamine	1846	169.133866	0.002433333	pos
Philanthotoxin 343	69713	418.317204	9.91855	pos
Phloionolic acid	35480	355.246184	12.74158333	pos
Phomopsin B	HMDB0038884	799.315169	13.83476667	neg
Phosphatidyl glycerol	186	245.043546	0.76405	neg
Phosphine-biotin	64823	791.326889	14.14051667	neg
Phosphocholine	3318	184.073715	0.712516667	pos
Phosphocreatine	HMDB0001511	212.043521	0.786766667	pos
Phosphodimethylethanolamine	HMDB0060244	168.042503	0.710516667	neg
Phosphonoacetate	HMDB0004110	184.985257	0.905383333	neg
Phosphoramide mustard	HMDB0060689	264.991848	9.75215	neg
Phosphorylcholine	HMDB0001565	202.107798	1.007533333	pos
p-hydroxyfosinoprilat	2977	541.303284	9.85735	pos
p-Hydroxyphenylacetic acid	130	135.044428	1.802133333	pos
p-Hydroxyphenylbutazone glucuronide	1845	545.178961	7.012216667	neg
p-HydroxyPiroxicam glucuronide	2034	541.122129	15.86195	pos
Phytosphingosine	7066	318.3009	8.859766667	pos
Phytosulfokine b	HMDB0029810	763.183848	0.905383333	neg
PI(13:0/12:0)	LMGP06010036	751.381159	13.77301667	pos
PI(15:0/0:0)	81195	581.270923	4.160016667	pos
PI(17:2(9Z,12Z)/0:0)	81180	621.242318	10.53256667	pos
PI(18:3(6Z,9Z,12Z)/18:2(9Z,12Z))	LMGP06010347	895.470891	4.7158	pos
PI(19:0/0:0)	LMGP06050027	653.307319	4.5058	pos
PI(20:1(11Z)/0:0)	81184	609.340184	11.95526667	pos
PI(20:4(5Z,8Z,11Z,14Z)/0:0)	46748	603.293625	11.85445	pos
PI(22:1(11Z)/0:0)	LMGP06050022	699.374951	13.59966667	neg
PI3-Kinase alpha Inhibitor 2	45221	294.070016	0.7282	neg
Picolinic acid	HMDB0002243	141.066182	0.905683333	pos
Picrocrocin	58320	348.202379	9.391516667	pos
Pikromycin	LMPK04000038	526.335921	9.7543	pos
Pilocarpine	HMDB0015217	415.234705	9.562733333	neg
Piloty's Acid	44964	174.022271	4.59005	pos
Pimelic acid	HMDB0000857	159.065624	4.737533333	neg
Pimozide	1938	444.224946	2.585966667	pos
Pinacidil	1941	246.171938	15.94263333	pos
Pindolol	1943	231.149681	5.157166667	pos
Pinnasterol	LMST01010283	485.265835	10.22716667	pos
Pioglitazone	HMDB0015264	401.117641	8.972016667	neg
PIP(18:2(9Z,12Z)/16:0)	61364	937.485172	6.144733333	pos
Piperalol	HMDB0035767	253.180274	8.982766667	pos
Piperettine	HMDB0034371	312.159829	10.49155	pos
Pipericine	HMDB0031678	336.326589	14.35173333	pos
Piperine	43568	286.144349	9.836716667	pos
Piperonyl sulfoxide	72888	369.175022	8.950933333	neg
Pirbuterol	66744	241.154964	4.076883333	pos
piritramide	96198	431.280637	12.64121667	pos
PISCIDIC ACID	43637	274.09271	0.905683333	pos
Pivaloylcarnitine	HMDB0041993	246.170503	4.59005	pos
PKHdiA-PE	LMGP20020016	590.311754	4.097783333	pos
PKODA-PS	LMGP20040002	686.367564	13.45395	neg
PKODiA-PA	LMGP20070008	579.293759	3.640883333	pos
PKOOA-PI	LMGP20050006	769.343521	12.47065	neg
Plakortic acid	71590	299.222201	10.57348333	pos

Plantagonine	89303	178.08663	5.015533333	pos
Plerixafor	HMDB0015681	503.455848	13.72018333	pos
p-Menth-3-ene-3-thiol	92928	385.223972	9.351683333	neg
p-Nitrophenyl-O-ethyl ethylphosphonate	69527	240.04359	5.916916667	neg
Polidocanol	69582	600.469125	12.29868333	pos
Poloxalene	43312	145.08625	6.04325	neg
Polyethylene, oxidized	88485	243.124135	6.106483333	neg
Polysorbate 60	HMDB0037183	457.277963	11.33671667	pos
Porphobilinogen	76	227.103013	1.090316667	pos
Porrigenin A	41795	449.327898	11.25935	pos
Porson	HMDB0030810	425.136946	9.473266667	pos
Pralidoxime	HMDB0014871	273.135143	6.548766667	neg
Pramanicin	53948	414.211971	10.21556667	neg
Pranlukast	HMDB0015481	520.140486	1.9283	pos
Pravastatin	HMDB0005022	405.226716	10.15231667	neg
Precocene II	67355	221.117676	0.548016667	pos
Pregabalin	HMDB0014375	198.089429	0.786766667	pos
Pregnanediol	63335	365.270508	11.7843	neg
Prenyl benzoate	HMDB0032488	379.192279	10.90441667	neg
Prenyl-L-cysteine	66519	377.158947	9.393866667	neg
Pretetramid	72131	374.063811	9.878016667	pos
Prilocaine	2073	221.1653	4.779033333	pos
Pristanal	HMDB0001958	283.299971	0.14905	pos
Pristanic acid	HMDB0000795	299.29482	0.002433333	pos
Procarbazine	2105	220.146611	9.62565	neg
Prolyl-Glutamine	85913	288.120876	1.09225	neg
Prolyl-Tryptophan	HMDB0029028	300.135497	7.518333333	neg
Promazine N-oxide sulfoxide	2134	631.242351	9.14075	neg
Propanoylagmatine	65794	169.145099	14.43878333	pos
Propantheline	HMDB0014920	386.257312	5.918766667	pos
Propiconazole	68862	386.067879	8.2549	neg
Propofol glucuronide	HMDB0060933	353.161255	7.622216667	neg
Propyl 1-(propylthio)propyl disulfide	HMDB0033040	447.137437	11.17246667	neg
Propyl 2,4-decadienoate	HMDB0037307	228.196195	13.52618333	pos
Propyl 2-furanacrylate	92556	181.086264	0.548016667	pos
Propyleine	263995	209.201643	6.124133333	pos
Propylene glycol stearate	86471	365.302972	9.0643	pos
Prosulfuron	68715	464.084469	7.7066	neg
Protionamide sulfoxide	2180	179.064144	5.589866667	pos
PS(12:0/13:0)	3870	618.37974	13.3807	neg
PS(13:0/18:4(6Z,9Z,12Z,15Z))	77745	731.459721	9.959816667	pos
PS(14:0/13:0)	77757	648.425709	9.370933333	pos
PS(14:0/14:0)	40813	662.4418	10.00103333	pos
PS(14:0/15:0)	HMDB0112273	676.456729	10.55303333	pos
PS(14:1(9Z)/15:0)	77776	692.451367	9.3299	pos
PS(16:0/14:1(9Z))	77857	706.467341	9.959816667	pos
PS(16:1(9Z)/15:0)	77869	720.482842	10.49155	pos
PS(16:1(9Z)/18:3(9Z,12Z,15Z))	HMDB0012370	738.467322	10.20713333	pos
PS(17:2(9Z,12Z)/22:6(4Z,7Z,10Z,13Z,16Z,19Z))	LMGP03010307	835.523984	15.39966667	pos
PS(18:1(9Z)/0:0)	40829	504.272122	11.27475	neg
PS(18:1(9Z)/0:0)[U]	40832	524.299673	11.59303333	pos
PS(20:2(11Z,14Z)/18:3(9Z,12Z,15Z))	78237	790.502688	13.90668333	neg
PS(20:3(8Z,11Z,14Z)/0:0)	78849	530.287364	10.22716667	pos
PS(22:6(4Z,7Z,10Z,13Z,16Z,19Z)/0:0)	78840	568.266955	10.67736667	neg
PS(22:6(4Z,7Z,10Z,13Z,16Z,19Z)/15:0)	78485	838.486042	13.70833333	neg

PS(O-16:0/0:0)	78863	522.258059	10.6551	pos
PS(O-16:0/16:1(9Z))	LMGP03020007	742.496084	10.4711	pos
PS(O-20:0/0:0)	78861	540.364296	10.16615	pos
PS(P-18:0/13:0)	78769	728.480401	9.959816667	pos
Pseudoephedrine	HMDB0001943	166.122987	0.002433333	pos
Pseudooxynicotine	HMDB0001240	355.213315	9.457166667	neg
Pseudouridine	5734	262.103926	0.806416667	pos
Pseudouridine 5'-phosphate	HMDB0001271	305.019528	1.09225	neg
PSOROMIC ACID	43642	357.062518	7.601116667	neg
Pteroic acid	66690	357.094149	8.465833333	neg
Pteroside P	HMDB0036608	441.17779	9.457166667	neg
Pterosin N	92947	252.159786	3.972533333	pos
Purine	6193	138.077824	15.98305	pos
Pyranocyanin A	90710	632.17407	9.646666667	neg
Pyrazinamide	2196	141.077511	15.92241667	pos
Pyridafenthion	72534	339.057591	4.927216667	neg
Pyridine N-oxide glucuronide	HMDB0061177	273.084898	1.090316667	pos
Pyridoxal	HMDB0001545	166.050349	8.508033333	neg
Pyridoxal Phosphate	235	285.988893	0.6755	pos
Pyridoxamine	238	151.086869	2.032866667	pos
Pyrimethanil	HMDB0033135	238.074084	8.27325	pos
Pyrocatechol	282	219.066329	6.359216667	neg
Pyroglutamic acid	3251	130.050311	1.090316667	pos
Pyruvic acid	LMFA01060077	175.024261	1.09225	neg
PYRVINIUM	44259	382.229131	5.466183333	pos
Quercetin 3-(6''-acetylglucoside)	LMPK12112138	551.103418	14.8218	neg
Quercetin 3,7,3',4'-tetra-O-sulfate	LMPK12112313	602.850619	12.00708333	neg
Quercetin 3-glucosyl-(1->2)-galactoside	50462	607.12782	0.884666667	neg
Questiomycin A	HMDB0030483	257.056708	7.49725	neg
Quetiapine	58287	422.129221	6.062316667	pos
Quinalphos	68785	299.062283	0.002433333	pos
Quindoxin	88831	207.040926	5.348033333	neg
Quinoline-3-carboxamides	69160	404.105359	0.823166667	neg
Quinone	6905	215.034978	6.064333333	neg
Quinoxaline	72378	148.087414	15.94263333	pos
QUIPAZINE	44221	236.114781	15.92241667	pos
Radicicol	45457	345.05509	5.7905	neg
Rasagiline	85505	172.112406	4.65325	pos
Resolvin D2	LMFA04030001	421.223923	10.53113333	neg
Resveratrol	LMPK13090005	246.111388	11.33671667	pos
Retigabine	69720	321.171333	5.3837	pos
Rhazidigenine Nb-oxide	86843	295.182609	9.62565	neg
Rhodinyl acetate	HMDB0037186	243.160464	8.318183333	neg
Rhodinyl isovalerate	92157	241.21672	0.002433333	pos
Rhodinyl propionate	92154	195.174805	11.11933333	pos
Riboflavin	HMDB0000244	377.146427	4.779033333	pos
Ribose-1-arsenate	HMDB0012285	272.958435	1.112466667	neg
Riccionidin A	LMPK12010445	266.020818	4.211566667	neg
Ricinoleic acid	35485	343.249677	10.594	neg
Riesling acetal	92419	271.155875	7.159866667	neg
Rilmenidine	68861	359.244844	9.372783333	neg
Riluzolamide	2332	553.021189	5.348033333	neg
Riluzole	HMDB0014878	272.970285	15.07588333	pos
Risedronate	HMDB0015022	281.994712	4.211566667	neg
Robustic acid	LMPK12160025	419.08833	5.280883333	pos

Ropinirole	2361	305.187871	11.19298333	neg
Rotigotine	85553	296.148594	6.759416667	neg
Rotundine A	90578	276.161062	11.88563333	neg
Ruscopine	67031	329.194455	6.389116667	pos
S-(2-Furanylmethyl) methanethioate	HMDB0037731	283.010927	4.211566667	neg
S-(4,5-Dihydro-2-methyl-3-furanyl) ethanethioate	92601	176.074354	4.055983333	pos
Sabinene hydrate	41075	172.169905	9.529233333	pos
S-Acetylthioethanolamine	65997	237.074704	5.116983333	neg
S-Adenosyl-4-methylthio-2-oxobutanoate	3518	442.105338	5.20055	neg
S-Adenosylhomocysteine	296	385.129515	1.090316667	pos
S-Adenosylmethioninamine	3501	353.140228	10.46825	neg
Sakacin P	HMDB0038239	217.118764	1.090316667	pos
Salicin	6948	267.088015	9.119666667	neg
Salicylamide	43329	138.055351	5.177783333	pos
Salicylic acid	616	183.029498	5.2427	neg
Salithion	72740	260.999182	0.6902	neg
Sambutoxin	HMDB0041085	476.278091	10.24565	pos
S-aminomethyldihydrolipoamide	LMFA08010025	471.196296	11.2542	neg
Sanguisorbic acid dilactone	86146	453.007055	4.219483333	pos
Sarpagine	64351	355.167212	7.622216667	neg
Sativol	48350	343.046639	5.57985	neg
Saxitoxin	69686	344.133373	7.32855	neg
SB 939	96423	341.232932	10.06316667	pos
Schradan	72766	267.114707	9.77325	neg
Scillirosidin	LMST01130002	460.270393	10.22716667	pos
S-cucujolide V	LMFA07040049	205.159047	0.02505	pos
SE 175	62987	693.087112	11.39748333	neg
Sebacic acid	LMFA01170006	201.113012	6.464533333	neg
Secoeremopetasitolide A	95769	731.327408	14.28501667	neg
Sedoheptulose 7-phosphate	3418	289.03362	0.7282	neg
Semilepidinoside A	88998	317.114311	7.32855	neg
Septentrionine	67140	759.371151	4.421633333	neg
Serinyl-Gamma-glutamate	85946	214.083255	13.7265	neg
Serratamic acid	LMFA00000002	276.181176	6.409216667	pos
Serylcysteine	HMDB0029036	415.094575	7.455083333	neg
Sesamex	72887	321.131641	15.98305	pos
Sevoflurane	HMDB0015366	180.988772	5.643033333	neg
S-Glutathionyl-L-cysteine	63433	444.120271	15.49035	pos
Shikonin	64009	269.082629	8.191633333	neg
Shyobunyl acetate	HMDB0036153	287.1986	15.47191667	pos
Sialyl Lea tetra	HMDB0006633	963.331995	13.54503333	neg
Sinalbin A	91694	279.02741	0.710516667	neg
Sinapyl alcohol	HMDB0013070	255.087944	5.432366667	neg
Siphonaxanthin ester/ Siphonaxanthin dodecenoate	LMPR01070159	803.559435	15.3814	pos
SITS	53243	498.995946	4.211566667	neg
S-Japonin	HMDB0035802	381.174588	13.90668333	neg
S-Lactoylglutathione	HMDB0001066	362.100544	4.179616667	pos
SM(d18:0/0:0)	41562	449.349724	10.04253333	pos
SM(d18:2/22:1)	LMSP03010070	821.594298	15.30908333	pos
sn-glycero-3-phosphoethanolamine	96256	216.06364	0.712516667	pos
Sodium (±)-2-(4-methoxyphenoxy)propionate	94786	217.048113	0.74595	neg
Sodium citrate	69553	274.976832	9.372783333	neg
Sodium oleate	72401	349.2369	11.58091667	neg
Sodium tetradecyl sulfate	2469	293.179969	14.43023333	neg
Sonchuionoside C	HMDB0035212	431.193261	13.56321667	neg

Sophoracoumestan A	48327	379.083295	0.823166667	neg
Sorbaldehyde	36528	191.107434	9.119666667	neg
Sorbitan laurate	HMDB0029885	369.225504	11.43183333	pos
Sorbitan oleate	86565	411.31123	10.83661667	pos
Sorbitan palmitate	HMDB0029887	385.293204	12.46005	pos
Sorbitan stearate	HMDB0029888	411.312571	11.94636667	neg
Sorbitol-6-phosphate	58327	261.038651	5.874783333	neg
Sparfloxacin	66707	437.162662	8.212733333	neg
Spectinomycin adenylate	2477	684.202765	16.00321667	pos
Spenolimycin	71966	391.172419	9.225133333	neg
S-Phenylmercapturic acid	HMDB0042011	477.117983	5.348033333	neg
Sphinganine	395	302.30601	9.878016667	pos
Sphinganine-phosphate	3512	404.255173	1.865133333	pos
Sphingofungin C	LMSP01080063	454.27853	12.2583	pos
Sphingosine	392	300.290219	10.12496667	pos
Sphingosine 1-phosphate (d19:1-P)	HMDB0060062	434.245072	9.473266667	pos
Sphingosine-1-phosphocholine	LMSP01060001	487.326968	8.72235	pos
Spinatoside	LMPK12112918	521.094618	6.633	neg
Spirolide F	87020	750.492952	9.939183333	pos
S-Prenyl-L-cysteine	HMDB0012286	377.156714	8.52915	neg
Squalamine	LMST05050024	666.427271	5.7342	pos
SR95531	69708	332.126084	4.758616667	neg
Stearaldehyde	LMFA06000098	286.310918	9.878016667	pos
Stearoyllactic acid	HMDB0033372	339.289862	11.11933333	pos
Stearyl citrate	HMDB0032521	427.304123	13.17741667	pos
Stigmast-4-ene-3,6-dione	92834	465.314014	8.72235	pos
Stoloniferone G	LMST01031094	499.281472	10.22716667	pos
Streptidine	65537	263.146815	0.8261	pos
Streptidine 6-phosphate	64559	387.102336	8.086216667	neg
Streptoval C	85109	812.315947	0.6532	neg
Styrene cis-glycol	66572	139.075678	1.048966667	pos
SU 11652	45563	827.357526	13.67216667	neg
Suberic acid	4243	173.081447	5.2427	neg
Succinic acid	HMDB0000254	163.024265	1.071433333	neg
Succinic acid semialdehyde	275	203.055916	3.979733333	neg
Succinylproline	69170	429.149855	10.21556667	neg
Sucrose	HMDB0000258	387.115305	0.905383333	neg
Sudan Brown RR	73207	523.238482	12.5505	neg
Sugeonyl acetate	53457	315.134567	6.062316667	pos
Suillin	89868	458.325673	13.07931667	pos
Sulbactam sodium	69757	238.013866	0.806416667	pos
Sulfapyridine	HMDB0015028	230.039611	0.6902	neg
Sulfate	HMDB0001448	194.927308	0.905383333	neg
Sulfobacin SL8	LMSP00000023	628.403588	10.32751667	pos
Sulfoglycolithocholate	LMST05030004	512.269794	9.18295	neg
Sunitinib	45411	397.206282	7.95965	neg
Suxibuzone	HMDB0042019	483.178339	9.541616667	neg
Symmetric Dimethylarginine	44873	201.135365	0.864066667	neg
Tafluprost	44903	451.230067	10.73845	neg
Talaromycin A	86753	253.141777	15.92241667	pos
Talinumoside I	HMDB0040957	861.426093	5.672333333	pos
Tamoxifen-N-glucuronide	HMDB0060616	549.273102	16.00321667	pos
Tanacetol A	HMDB0035722	293.176609	9.62565	neg
Tanacetol B	HMDB0035075	341.197613	8.191633333	neg
Taraxinic acid glucosyl ester	90559	469.17258	10.17338333	neg

Taurine	31	126.022407	0.712516667	pos
Taurochenodeoxycholic acid	57991	498.29095	8.42365	neg
Taxa-4(20),11(12)-dien-5alpha-acetoxy-10beta-ol	LMPR0104390006	391.247649	10.86301667	neg
Tecostanine	68068	228.160674	6.759416667	neg
Temozolomide	85179	239.054024	5.4745	neg
Temurin	HMDB0004328	247.079514	5.857883333	pos
Tephrowatsin B	LMPK12020283	335.166187	11.47903333	neg
TEPP	72768	271.050792	5.7694	neg
Terbumeton	72539	226.165476	13.73791667	pos
Terbutaline	2762	243.170699	4.547916667	pos
Terephthalic acid	HMDB0002428	165.018719	4.716466667	neg
Terfenadine	2765	472.321986	9.3094	pos
Testosterone enanthate	2785	381.280611	13.74465	neg
Testosterone phenylpropionate	70245	459.229509	4.842316667	pos
Tetracaine	2807	303.147617	4.118683333	pos
Tetracaine N-oxide	2808	261.160916	7.32855	neg
Tetracenomycin B1	63753	368.114218	7.495216667	pos
Tetracosanedioic acid	35991	399.347772	0.02505	pos
Tetradecanedioic acid	LMFA01170018	257.176384	9.77325	neg
Tetradecanoylcarnitine	HMDB0005066	372.311438	10.20713333	pos
Tetraethylene glycol	HMDB0094708	217.10515	15.98305	pos
Tetrahydroaldosterone-3-glucuronide	LMST05010067	585.257497	10.67736667	neg
Tetrahydrobiopterin	HMDB0000027	259.152219	5.115883333	pos
Tetrahydrocorticosterone	HMDB0000268	368.280068	9.412066667	pos
Tetrahydrocortisol	LMST02030143	365.231707	10.594	neg
Tetrahydrodeoxycorticosterone	5840	379.247519	10.92508333	neg
Tetrahydrodeoxycortisol	57990	395.244355	10.29983333	neg
Tetrahydrodipicolinate	62916	152.034575	7.3918	neg
Tetrahydropentoxyline	86646	367.150625	2.7309	pos
Tetrahydropersin	91488	429.323093	10.78	neg
Tetranor-8-NO2-CLA	LMFA01120009	250.145227	11.80461667	neg
Tetranor-PGF1alpha	74978	283.190762	9.247933333	pos
TG(12:0/18:3(9Z,12Z,15Z)/18:4(6Z,9Z,12Z,15Z))[iso	LMGL03013519	831.590699	15.3633	pos
TG(14:0/19:0/22:0)[iso6]	LMGL03014460	943.805669	4.097783333	pos
TG(14:1(9Z)/20:0/21:0)[iso6]	LMGL03014892	883.814178	15.98875	neg
TG(15:0/14:0/o-18:0)	HMDB0042988	796.775138	14.66688333	pos
TG(15:0/18:4(6Z,9Z,12Z,15Z)/o-18:0)	HMDB0043684	844.778988	0.641016667	pos
TG(15:0/22:2(13Z,16Z)/22:6(4Z,7Z,10Z,13Z,16Z,19Z	LMGL03015349	925.766307	14.9938	neg
TG(15:0/22:4(7Z,10Z,13Z,16Z)/o-18:0)	HMDB0043597	927.802114	15.45051667	neg
TG(16:0/18:0/16:0) (d5)	4710	884.800899	15.18495	neg
TG(18:0/18:0/18:0)	4701	871.811345	0.6532	neg
TG(19:0/20:1(11Z)/20:4(5Z,8Z,11Z,14Z))[iso6]	37740	931.80957	13.49036667	neg
TG(20:0/20:1(11Z)/20:4(5Z,8Z,11Z,14Z))	HMDB0005419	945.826488	14.32125	neg
TG(22:5(4Z,7Z,10Z,13Z,16Z)/22:6(4Z,7Z,10Z,13Z,1	HMDB0055140	947.784115	0.6532	neg
TG(8:0/8:0/8:0)	HMDB0011187	493.349461	11.33671667	pos
Theaspirone A	90542	209.154156	9.733716667	pos
Thebaine	64353	334.140259	4.75795	pos
Thelephoric acid	87056	351.013074	4.927216667	neg
Thiabendazole	2823	200.029515	4.211566667	neg
Thiacloprid	72318	233.006984	4.758616667	neg
Thiamethoxam	72319	581.030197	0.6532	neg
Thiamine	229	265.11228	0.866166667	pos
Thidiazuron	72579	439.076685	0.823166667	neg
Thiodiacetic acid	HMDB0042032	151.005529	0.584866667	pos
Thiolutin	HMDB0034228	208.984764	0.6902	neg

Thiometon	72450	226.978414	4.948316667	neg
Thiomorpholine 3-carboxylate	66076	192.033301	1.09225	neg
Thioridazine	2835	351.135174	9.62565	neg
Thonningianin B	LMPK12120466	767.143893	9.77325	neg
Thonzylamine	HMDB0240222	269.175383	8.41665	pos
Threonic acid	HMDB0000943	135.030406	1.09225	neg
Threoninyl-Asparagine	HMDB0029056	214.083395	15.90565	neg
Threoninyl-Aspartate	85950	235.092979	0.786766667	pos
Thromboxane B2	HMDB0003252	388.269718	9.980383333	pos
THTC	68817	150.05865	1.090316667	pos
Thymidine glycol	HMDB0042036	321.093791	6.885783333	neg
Thyrotropin releasing hormone	HMDB0060080	361.162703	10.21556667	neg
Tiaprofenic acid	2844	305.047841	2.155183333	neg
Tiaprost	96518	378.152262	10.1945	neg
Ticarcillin disodium	69762	473.005079	10.21556667	neg
Tiletamine	43352	206.100831	4.160016667	pos
Tiludronate	HMDB0015265	316.921725	4.948316667	neg
Tingenone	67088	421.274511	10.99823333	pos
Tocainide	2864	383.244702	10.38415	neg
Tocopheronic acid	HMDB0030555	317.136545	9.878016667	pos
Tolazoline	2867	178.134394	15.94263333	pos
Toxin T2 tetrol	91310	299.149459	5.610483333	pos
trans, trans-3,5-Octadien-2-one	88467	247.170785	11.2542	neg
trans-2-trans-4-Nonadiene	88512	184.20641	5.796033333	pos
trans-3-Chloroacrylic acid	HMDB0060515	128.97151	0.584866667	pos
trans-3-Hydroxycotinine glucuronide	6080	369.129242	15.86195	pos
trans-9-palmitoleic acid	LMFA01030057	299.22339	10.90441667	neg
trans-Cinnamic acid	63104	193.050315	5.853733333	neg
trans-Ferulic acid	HMDB0000954	177.055012	9.549883333	pos
trans-Hexadec-2-enoyl carnitine	LMFA07070109	420.30928	11.97545	pos
trans-S-(1-Propenyl)-L-cysteine	86269	162.058489	2.296783333	pos
trans-Tephrostachin	LMPK12110168	365.140818	11.92615	neg
trans-trans-Muconic acid	HMDB0002349	180.99018	3.371866667	pos
trans-Zeatin	45500	202.109252	15.98305	pos
Traumatic acid	35963	229.143949	0.526866667	pos
Traumatin	HMDB0037326	257.139954	6.801533333	neg
Treosulfan	HMDB0042046	316.975435	4.238933333	pos
TrHA	74389	343.26396	13.02021667	pos
Triacanthine	66973	405.226885	10.61491667	neg
Triamcinolone	2902	393.172778	14.46681667	neg
Triamcinolone acetonide glucuronide	2906	611.25028	10.16615	pos
Tributyl phosphate	70062	265.158128	9.920666667	neg
Tributyrin	69736	301.166347	6.864716667	neg
Tricosanoylglycine	HMDB0013314	429.4061	13.80811667	pos
Tricrozarin A	68310	332.999791	9.878016667	pos
Tridecanal	HMDB0030928	199.206056	0.002433333	pos
Tridecanamide	97432	196.206464	5.507416667	pos
Tridecanoylglycine	HMDB0013317	272.222613	9.1661	pos
Tridecyl phloretate	HMDB0035477	393.263331	11.2542	neg
Tridihexethyl	HMDB0014648	341.269316	10.26573333	pos
Triethanolamine	HMDB0032538	194.103117	0.6902	neg
Triethyl citrate	89812	299.110736	7.59775	pos
Triethylene glycol diglycidyl ether	73214	243.124139	7.6855	neg
Trifluoroacetic acid	HMDB0014118	226.978456	4.211566667	neg
Trifluoromethyl-bismethyl ketone	HMDB0061928	185.042713	4.042966667	neg

Trifoliol	48344	343.046627	5.36915	neg
Trigoforin	86305	233.082294	6.991116667	neg
Trigonelline	273	138.05528	0.8261	pos
Trimethylpyrazine	87965	140.118643	15.98305	pos
Trinitrotoluene	71179	207.99915	0.6902	neg
Trioxilin A3	HMDB0001977	355.248564	9.939183333	pos
Tripamide	HMDB0042054	350.072511	2.134433333	neg
Triphenylphosphine oxide	64889	279.094125	8.762433333	pos
Triphenylpropargylphosphonium	85162	346.112641	4.211566667	neg
Triphenylsilanol	70148	275.09093	8.5924	neg
Tris(butoxyethyl)phosphate	70068	399.251509	11.47181667	pos
Troglitazone	2970	881.316979	14.412	neg
Tryptamine	325	178.134384	15.63241667	pos
Tryptophanol	HMDB0003447	200.047613	5.610483333	pos
Tryptophyl-Tryptophan	HMDB0029094	389.162302	12.49058333	neg
Tsuzuic acid	LMFA01030048	271.192205	10.21556667	neg
Tuberonic acid	36075	451.234825	10.9664	neg
Tuftsin	58305	518.341137	15.92241667	pos
Tumonoic Acid I	65437	515.367751	11.21971667	pos
TXB1	36262	373.258129	10.00103333	pos
TXB2	LMFA03030002	371.243569	10.24565	pos
TXB2-d4	36264	419.260032	11.0077	neg
Tyraminium	HMDB0062629	177.05502	5.857883333	pos
Ubiquinone-1	45118	251.128339	10.36856667	pos
UDP-N-acetylmuramoyl-L-alanine	63631	795.138883	9.75215	neg
UNC0321	96743	516.367676	12.88041667	pos
UNC0638	96443	527.40476	15.0222	pos
Undecanal	HMDB0030941	193.159109	0.002433333	pos
Undecanedioic acid	5846	199.133319	1.028233333	pos
Unoprostone	45686	400.306495	10.87708333	pos
Unoprostone isopropyl ester	45687	469.31804	10.57303333	neg
Unshuoside A	86491	331.177004	5.959016667	neg
Uplandicine	68316	356.1723	9.62565	neg
Uracil	HMDB0000300	130.06155	15.92241667	pos
Urdamycin F	63805	861.351187	13.36233333	neg
Uric acid	88	167.020468	1.09225	neg
Uridine	90	245.077256	1.108216667	pos
Urocortisol	41873	367.246222	11.8746	pos
Ursodeoxycholic acid	2982	437.291684	11.45865	neg
Uvaretin	LMPK12120469	417.108966	7.08555	pos
Valaciclovir	HMDB0014716	325.161094	0.622116667	pos
Valdiate	95421	309.171457	9.583833333	neg
Valerenal	53363	219.174807	11.49206667	pos
Valiolone	69312	237.061872	4.948316667	neg
Valproic acid	HMDB0001877	287.223513	9.75215	neg
Val-Val-Val	HMDB0094676	338.205731	4.358683333	pos
Valyl-Methionine	86025	266.152313	11.53246667	pos
Vanillin	62927	170.081502	0.002433333	pos
Vanillylamine	HMDB0012309	136.076032	1.2291	pos
Vanylglycol	HMDB0001490	229.071936	4.82185	neg
Ventosic acid	LMFA01050536	422.348374	13.15791667	pos
Vicenin 2	LMPK12110218	593.152322	5.20055	neg
Vildagliptin	HMDB0015596	605.380843	13.81676667	neg
Vitamin K3	3297	171.044643	5.853733333	neg
Vitisidin A	95973	380.052407	2.134433333	neg

Voglibose	HMDB0015598	268.139753	5.157166667	pos
Wharangin	51672	345.061017	5.363416667	pos
Withanolide A	89919	488.301611	10.22716667	pos
Wuhanic acid	LMFA01050559	441.323143	10.92508333	neg
Xanthine	82	151.025422	1.09225	neg
Xanthoangelol C	LMPK12120056	365.140863	11.66235	neg
Xanthoxin	64102	233.154	0.02505	pos
Xestoaminol C	53933	230.248283	8.820583333	pos
xi-4-Hydroxy-4-methyl-2-cyclohexen-1-one	HMDB0033629	144.102209	0.905683333	pos
xI-Dihydro-2-methyl-3(2H)-thiophenone	HMDB0038556	154.990572	0.584866667	pos
Y-27632 (hydrochloride)	44907	265.202841	4.65325	pos
Zaragozic acid C	6081	799.314955	14.30311667	neg
Zidovudine	HMDB0014638	250.093921	2.916283333	pos
Zingerone	71737	195.102055	8.68215	pos
ZK 159222	42547	530.382166	10.04253333	pos
Zonisamide	HMDB0015045	250.988524	3.910083333	pos
Zopiclone	HMDB0015329	371.101871	0.39995	pos
δ-CEHC	44826	251.128363	10.81631667	pos

Table S3 Detailed characteristics of TAO patients included in this study

No	Age (years)	Sex	Thyroid disease	Duration of TAO (months)	CAS	Phase of TAO	TAO treatment	Proptosis of the operated eye (mm)
1	38	Female	Graves' disease	35	2	inactive	GC (3.25g)	19.0
2	65	Male	Graves' disease	14	5	active	None	25.0
3	30	Female	Graves' disease	11	2	inactive	None	22.0
4	63	Female	Graves' disease	12	2	inactive	GC (6g)	24.0
5	62	Female	Graves' disease	15	2	inactive	None	20.0
6	51	Male	Graves' disease	22	0	inactive	None	20.0
7	70	Female	Graves' disease	7	3	inactive	None	18.0
8	67	Female	Graves' disease	8	4	active	GC (3g)	21.0
9	32	Male	Graves' disease	49	0	inactive	None	19.0
10	59	Female	Graves' disease	33	3	inactive	GC (4.5g)	20.0
11	67	Female	Graves' disease	26	4	active	None	24.0
12	22	Female	Graves' disease	60	0	inactive	None	22.0
13	31	Female	Graves' disease	120	0	inactive	None	20.5
14	32	Female	Graves' disease	19	0	inactive	GC (1.5g)	16.5
15	23	Female	Graves' disease	48	0	inactive	None	20.0

GC glucocorticoids, CAS clinical activity score
